# Correction: Access to primary healthcare during lockdown measures for COVID-19 in rural South Africa: an interrupted time series analysis

**DOI:** 10.1136/bmjopen-2020-043763corr1

**Published:** 2020-11-24

Siedner MJ, Kraemer JD, Meyer MJ, *et al*. Access to primary healthcare during lockdown measures for COVID-19 in rural South Africa: an interrupted time series analysis. *BMJ Open* 2020;10:e043763. doi: 10.1136/bmjopen-2020-043763

This article was previously published with an error in the x-axis of Figure 1. The correct figure is below:

**Figure:**
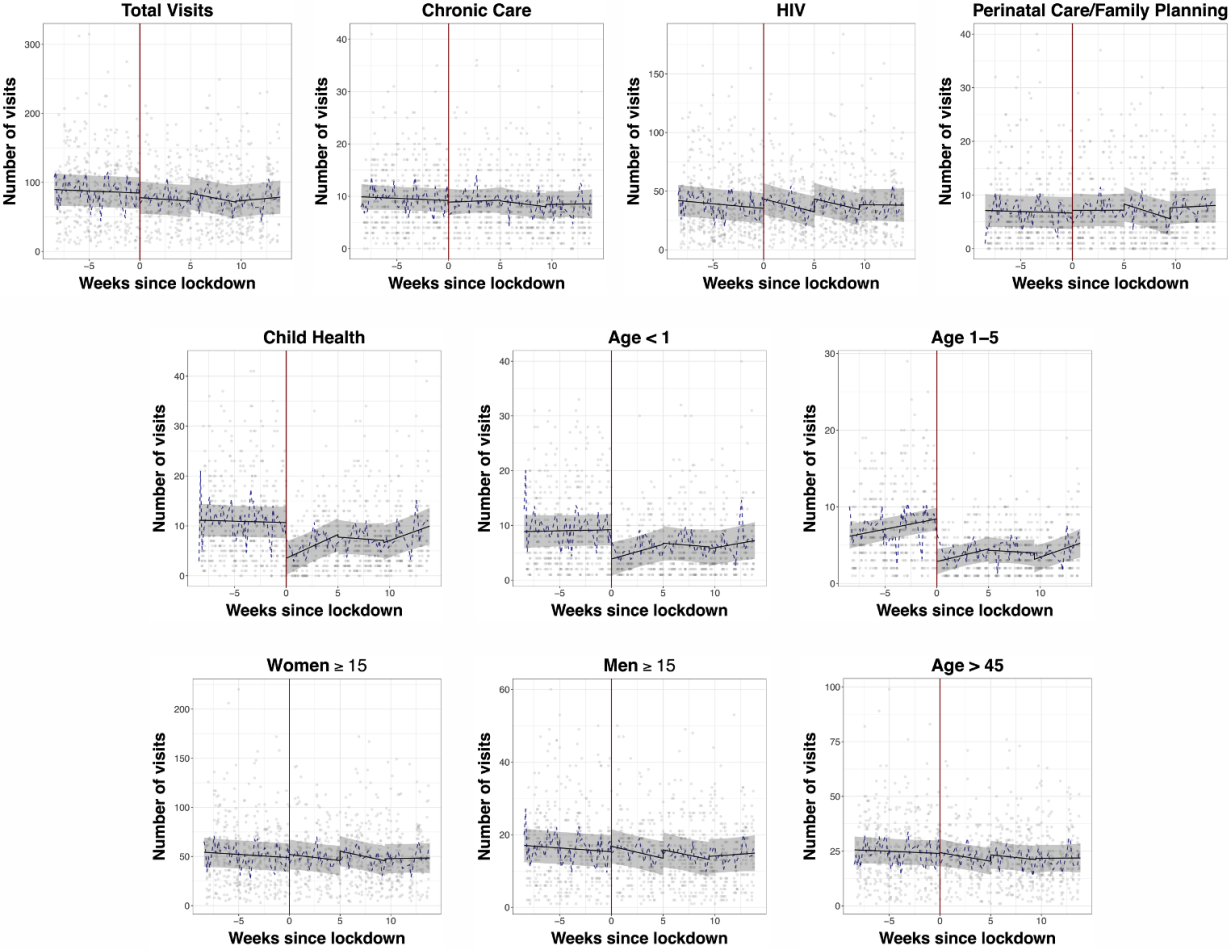


**Figure 1** Ambulatory clinic visitation before and after the nationwide lockdown in South Africa at 11 outpatient clinics in rural uMkhanyakude District, KwaZulu-Natal South Africa. Scatter plots represent mean clinic visitation at each clinic on weekdays during the observation period. The black fit line represents the mean visitation across all clinics estimated by postregression margins from a linear regression model, with a regression discontinuity coefficient at the date of the lockdown (27 March 2020, red line). Grey bars represent 95% CIs. The dotted blue line represents the geometic mean of the number of visits across all clinics on each day.

