# Supplementary material for: Access to primary healthcare during lockdown measures for COVID-19 in rural South Africa: an interrupted time series analysis
Source: BMJ Open. 2020 Oct 5;10(10):e043763. doi: 10.1136/bmjopen-2020-043763 (PMC7536636; doi:10.1136/bmjopen-2020-043763)

Supplemental Table 1. Mixed effects regression model results demonstrating changes in mean clinic visits by week, by visit type and demographic strata, in the pre- and post-lockdown period in uMkhanyakude District, KwaZulu-Natal South Africa.

Model	Weekly change in clinic visits/week during pre-lockdown period	P-value	Change in weekly change in clinic visits/week during level 5 lockdown period	*P*-value	Change in weekly change in clinic visits/week during level 4 lockdown period	*P*-value	Change in weekly change in clinic visits/week during level 3 lockdown period)	*P*-value
Total visits	-0.6 (-1.8, 0.6)	0.33	-0.4 (-3.3, 2.5)	0.80	-1.9 (-6.2, 2.4)	0.39	4.2 (-0.3, 8.7)	0.07
Child health[a]	-0.1 (-0.3, 0.2)	0.64	1.0 (0.5, 1.5)	<0.001	-1.1 (-1.9, -0.3)	0.01	0.9 (0.1, 1.7)	0.04
PNC and FP[b]	-0.1 (-0.2, 0.1)	0.54	0.1 (-0.4, 0.5)	0.78	-0.6 (-1.3, 0.1)	0.07	0.7 (0.0, 1.4)	0.05
HIV visits[c]	-0.8 (-1.6, 0.0)	0.04	-1.4 (-3.1, 0.3)	0.11	0.2 (-2.4, 2.7)	0.90	1.9 (-0.7, 4.6)	0.15
Chronic care[d]	-0.1 (-0.3, 0.1)	0.44	0.2 (-0.3, 0.6)	0.52	-0.3 (-1.0, 0.4)	0.37	0.3 (-0.5, 1.0)	0.47
Men ≥ 15	-0.2 (-0.5, 0.1)	0.15	-0.5 (-1.1, 0.2)	0.16	0.1 (-0.9, 1.0)	0.89	0.8 (-0.2, 1.8)	0.12
Women ≥ 15	-0.6 (-1.4, 0.1)	0.10	-0.6 (-2.5, 1.3)	0.55	-1.0 (-3.9, 1.9)	0.50	2.4 (-0.6, 5.4)	0.12
Age <1	0.0 (-0.2, 0.2)	0.71	0.5 (0.0, 1.0)	0.04	-0.7 (-1.4, 0.0)	0.06	0.5 (-0.2, 1.2)	0.19
Age 1-5	0.3 (0.1, 0.4)	<0.001	0.0 (-0.3, 0.4)	0.72	-0.4 (-0.9, 0.1)	0.11	0.5 (0.0, 1.1)	0.04
Age 6-19	0.1 (-0.1, 0.2)	0.27	-0.2 (-0.6, 0.1)	0.23	-0.2 (-0.8, 0.3)	0.45	0.7 (0.1, 1.2)	0.03
Age 20-45	-0.7 (-1.3, -0.1)	0.03	-0.4 (-1.9, 1.1)	0.59	-0.7 (-3.0, 1.5)	0.52	2.0 (-0.4, 4.4)	0.10
Age >45	-0.2 (-0.6, 0.2)	0.36	-0.6 (-1.6, 0.4)	0.25	0.3 (-1.2, 1.8)	0.70	0.5 (-1.0, 2.1)	0.50

[a]Child health: visits for immunizations and growth monitoring
[b]PNC and FP: perinatal care and family planning; visits for, antenatal care, prenatal care, and/or family planning
[c]HIV visits: visits for HIV testing, antiretroviral therapy initiation, antiretroviral therapy continuation, or pharmacy pick-up
[d]Chronic care: clinical visits for hypertension and/or diabetes

Supplemental Figure 1. Plots of residuals around estimated mean from linear mixed effects regression models of clinic visitation for total visits (A), child health visits (B), and HIV visits (C)

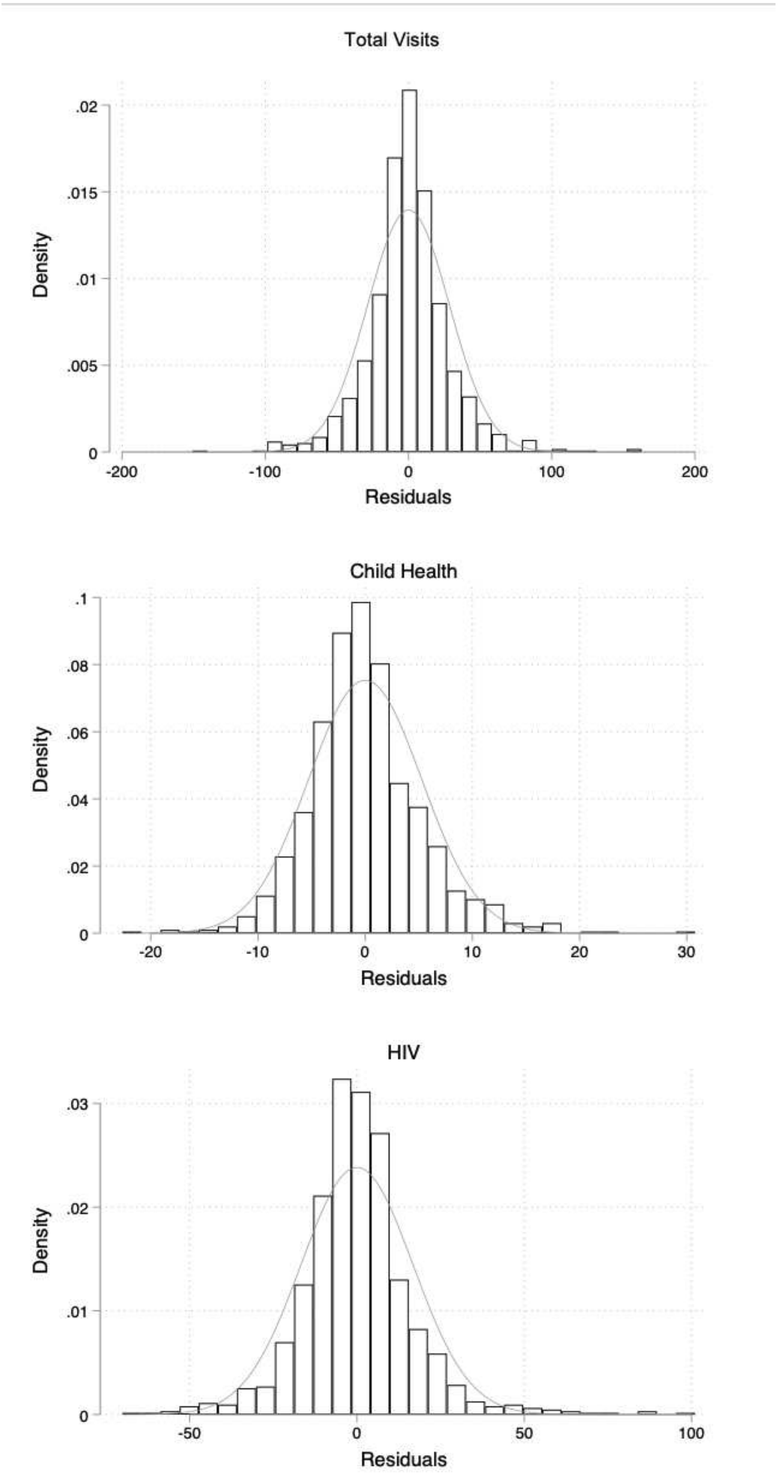

Supplemental Figure 2. Scatter and LOWESS plots with smoothed regression functions demonstrating clinic visits per day in the study observation period during 2019 and 2020 for all visits (A), child health visits (B), and HIV adults (C).

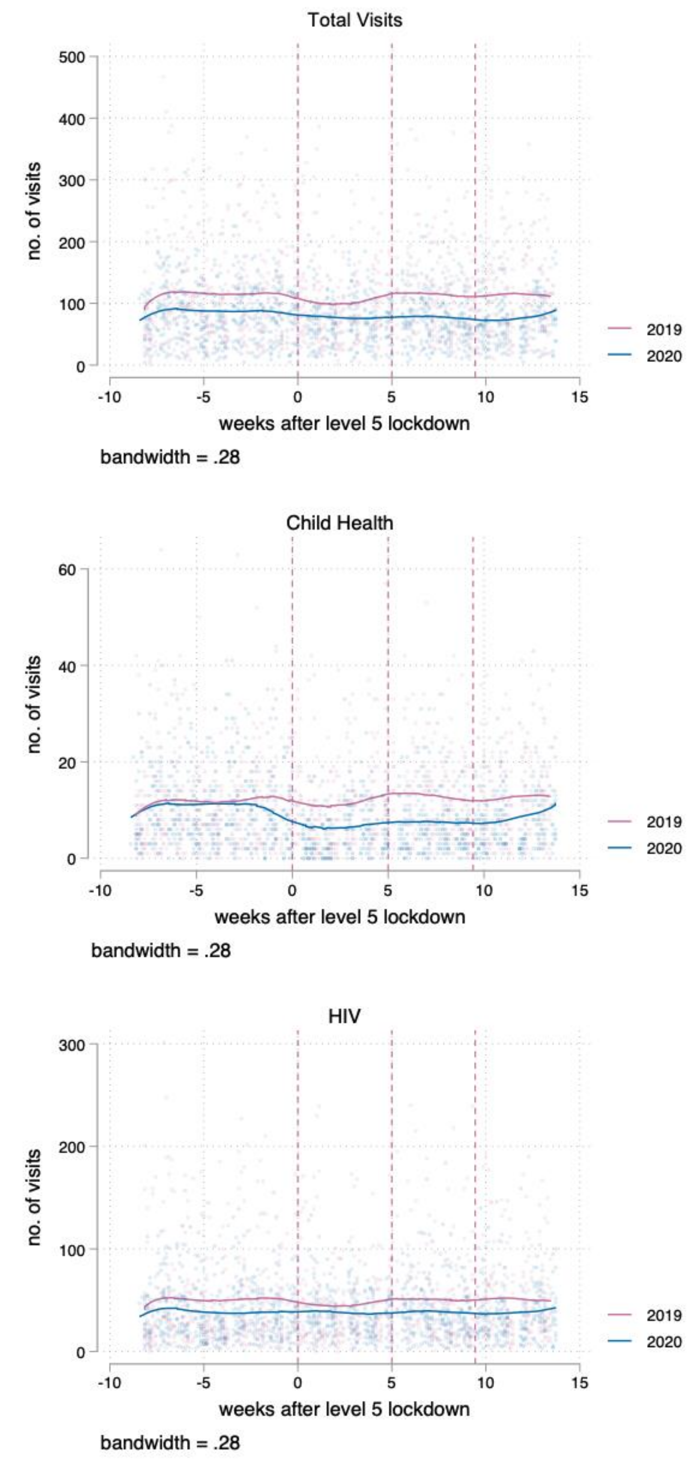

Supplement: Supplementary data [file bmjopen-2020-043763supp001.pdf]
