# Supplementary material for: Access to primary healthcare during lockdown measures for COVID-19 in rural South Africa: an interrupted time series analysis
Source: BMJ Open. 2020 Oct 5;10(10):e043763. doi: 10.1136/bmjopen-2020-043763 (PMC7536636; doi:10.1136/bmjopen-2020-043763)
Supplement: Author's manuscript [file bmjopen-2020-043763.draft_revisions.pdf]

BMJ Open

BMJ Open is committed to open peer review. As part of this commitment we make the peer review history of every article we publish publicly available.

When an article is published we post the peer reviewers' comments and the authors' responses online. We also post the versions of the paper that were used during peer review. These are the versions that the peer review comments apply to.

The versions of the paper that follow are the versions that were submitted during the peer review process. They are not the versions of record or the final published versions. They should not be cited or distributed as the published version of this manuscript.

BMJ Open is an open access journal and the full, final, typeset and author-corrected version of record of the manuscript is available on our site with no access controls, subscription charges or pay-per-view fees (http://bmjopen.bmj.com).

If you have any questions on BMJ Open's open peer review process please email info.bmjopen@bmj.com

BMJ Open

Access to primary healthcare during lockdown measures for COVID-19 in rural South Africa: an interrupted time series analysis

Journal:	*BMJ Open*
Manuscript ID	bmjopen-2020-043763
Article Type:	Original research
Date Submitted by the Author:	13-Aug-2020
Complete List of Authors:	Siedner, Mark; Africa Health Research Institute, Medicine; Harvard Medical School,<br>Kraemer, John; Georgetown University, Department of Health Systems Administratoin<br>Meyer, Mark; Georgetown University, Mathematics and Statistics<br>Harling, Guy; University College London, Institute for Global Health; Harvard University T H Chan School of Public Health, Department of Epidemiology<br>Mngomezulu, Thobeka; Africa Health Research Institute, Population research<br>Gabela, Patrick; Africa Health Research Institute<br>Dlamini, Siphephelo; Africa Health Research Institute<br>Gareta, Dickman; Africa Health Research Institute<br>Majozi, Nomathamsanqa; Africa Health Research Institute, Public Engagement<br>Ngwenya, Nothando; Africa Health Research Institute<br>Seeley, Janet; MRC/UVRI Uganda Research Unit On AIDS, ; London School of Hygiene and Tropical Medicine,<br>Wong, Emily; Africa Health Research Institute<br>Iwuji, Collins<br>Shahmanesh, Maryam; Africa Health Research Institute<br>Hanekom, Willem; Africa Health Research Institute<br>Herbst, Kobus; Africa Health Research Institute
Keywords:	COVID-19, Public health < INFECTIOUS DISEASES, International health services < HEALTH SERVICES ADMINISTRATION & MANAGEMENT

SCHOLARONE™
Manuscripts

I, the Submitting Author has the right to grant and does grant on behalf of all authors of the Work (as defined in the below author licence), an exclusive licence and/or a non-exclusive licence for contributions from authors who are: i) UK Crown employees; ii) where BMJ has agreed a CC-BY licence shall apply, and/or iii) in accordance with the terms applicable for US Federal Government officers or employees acting as part of their official duties; on a worldwide, perpetual, irrevocable, royalty-free basis to BMJ Publishing Group Ltd ("BMJ") its licensees and where the relevant Journal is co-owned by BMJ to the co-owners of the Journal, to publish the Work in this journal and any other BMJ products and to exploit all rights, as set out in our licence.

The Submitting Author accepts and understands that any supply made under these terms is made by BMJ to the Submitting Author unless you are acting as an employee on behalf of your employer or a postgraduate student of an affiliated institution which is paying any applicable article publishing charge ("APC") for Open Access articles. Where the Submitting Author wishes to make the Work available on an Open Access basis (and intends to pay the relevant APC), the terms of reuse of such Open Access shall be governed by a Creative Commons licence – details of these licences and which Creative Commons licence will apply to this Work are set out in our licence referred to above.

Other than as permitted in any relevant BMJ Author's Self Archiving Policies, I confirm this Work has not been accepted for publication elsewhere, is not being considered for publication elsewhere and does not duplicate material already published. I confirm all authors consent to publication of this Work and authorise the granting of this licence.

For peer review only

Access to primary healthcare during lockdown measures for COVID-19 in rural South Africa: an interrupted time series analysis

Authors

Mark J. Siedner*, John D. Kraemer, Mark J. Meyer, Guy Harling, Thobeka Mngomezulu, Patrick Gabela, Siphephelo Dlamini, Dickman Gareta, Nomathamsanqa Majozi, Nothando Ngwenya, Janet Seeley, Emily Wong, Collins Iwuji, Maryam Shahmanesh, Willem Hanekom, Kobus Herbst

***Corresponding Author:**

Mark J. Siedner
Africa Health Research Institute
KwaZulu-Natal, South Africa
mark.siedner@ahri.org

Conflicts of Interest

All authors report no conflicts

For peer review only

Abstract

Objectives

We evaluated whether implementation of lockdown orders in South Africa affected ambulatory clinic visitation in rural Kwa-Zulu Natal (KZN).

Design

Observational cohort

Setting

Data were analyzed from eleven primary healthcare clinics in northern KwaZulu-Natal.

Participants

A total of 46,523 individuals made 89,476 clinic visits during our observation period.

Exposure of Interest

We conducted an interrupted time series analysis to estimate changes in clinic visitation with a focus on transitions from the pre-lockdown to the level 5, 4, and 3 lockdown periods.

Outcome Measures

Daily clinic visitation at ambulatory clinics. In stratified analyses we assessed visitation for the following sub-categories: child health, perinatal care and family planning, HIV services, non-communicable diseases, and by age and sex strata.

Results

We found no change in total clinic visits/clinic/day at the time of implementation of the level 5 lockdown (change from 90.3 to 84.6 mean visits/clinic/day, 95%CI -16.5, 3.1), or at the transitions to less stringent level 4 and 3 lockdown levels. We did detect a greater than 50% reduction in child healthcare visits at the start of the level 5 lockdown from 11.9 to 4.7 visits/day (-7.1 visits/clinic/day, 95%CI -8.9, -5.3), both for children <1 and children 1-5, with a gradual return to pre-lockdown within three

For peer review only - http://bmjopen.bmj.com/site/about/guidelines.xhtml

months after the first lockdown measure. In contrast, we found no drop in clinic visitation in adults at the start of the level 5 lockdown, or related to HIV care (from 37.5 to 45.6, 8.0 visits/clinic/day, 95%CI 2.1, 13.8).

Conclusions

In rural KZN, we identified a significant, albeit temporary, reduction in child healthcare visitation but general resilience of adult ambulatory care provision during the first four months of the lockdown. Future work should explore the impacts of the circulating epidemic on primary care provision and longer-term impacts of reduced child visitation on outcomes in the region.

For peer review only - http://bmjopen.bmj.com/site/about/guidelines.xhtml

Strengths and Limitations of the Study

- We sought to empirically assess the effect of a nationwide lockdown in rural South Africa on access to primary care
- To do so, we leveraged an electronic medical information system that collects clinical data for ambulatory visits in the public health sector in rural KwaZulu-Natal
- We conducted an interrupted time series analysis to estimate changes in outpatient clinic visitation from 60 days before through the first four months of the lockdown period. We also stratified visits by type, age and sex.
- Our study is strengthened by a large number of observations per clinic, collection of data using research staff and methods external to the programmatic healthcare system (which can be interrupted during external shocks), and longitudinal observation allowing comparison of the pre- and post-period
- Limitations to our study include its generalizability to similarly rural, resource-limited settings, observation during the lockdown period but prior to widespread local COVID-19 transmission, the potential, however small, for a simultaneous shock to be responsible for the changes seen, and the quantitative nature of our data, which prevents deeper exploration of the root causes of trends noted

Keywords

COVID-19, South Africa, Primary Care, Health Systems Resilience, Health and Demographic Surveillance System

For peer review only - http://bmjopen.bmj.com/site/about/guidelines.xhtml

Introduction

COVID-19 was declared a global pandemic by the World Health Organization on 11th March 2020, and it has spared no region of the world. Early in the epidemic, the greatest numbers of cases have been reported in Asia, Europe and North America, with more recent dissemination within Latin America and Africa.[1] Although South Africa and a handful of other low-resource settings have reported widespread epidemics, limited testing and surveillance capabilities make it difficult to assess how widely the pandemic has spread in such settings. Such regions are believed to be at particular risk of severe epidemics, due to over-crowding, lower access to clean water and sanitation services, and inherent shortages in health system infrastructure for detection and management of disease.[2-9]

In response, most nations in sub-Saharan Africa have implemented non-pharmacologic interventions to attempt to prevent large scale epidemics. These measures, which include restrictions on large gatherings, work and school attendance, travel, and in their most stringent forms, shelter-in-place orders, are believed to reduce disease transmission.[10-13] However, instituting these measures is

For peer review only - http://bmjopen.bmj.com/site/about/guidelines.xhtml

also associated with deleterious economic and social, impacts, including large projected reductions in manufacturing, access to employment and basic necessities, and educational advancement; and these effects appear to be greatest among those in lower income and vulnerability categories.[14-19] Across the sub-Saharan African region, the Economic Commission for Africa projects an approximate 1.4% contraction in gross domestic product and that 25 million people are susceptible to entering extreme poverty.[20] Some have hypothesized that non-pharmaceutical interventions might be less effective in settings with large informal economies and limited ability to respond to increases in cases of severe disease,[21] and that their risks might outweigh their benefits.[22]

Of particular concern is how social fear and reduced access to basic public health services might impact morbidity and mortality for non-COVID health conditions. Modeling studies have suggested that even modest reductions in child healthcare access could result in 100,000s of additional deaths in low and middle-income countries.[23] Similar concerns have been raised by the Academy of Science of South Africa and others about provision of chronic disease care among adults.[24 25] UNAIDS

For peer review only - http://bmjopen.bmj.com/site/about/guidelines.xhtml

has warned that non-pharmaceutical interventions could challenge manufacturing and supply chains of HIV therapeutics,[26] and modeling estimates suggest that such disruptions could cause as many if not more HIV-related deaths than COVID-19-related deaths.[27] Although empiric data on health outcomes remain sparse, there have been significant reductions in tuberculosis testing in South Africa during the early phases of the lockdown,[28] indicating an interruption in critical services for the most common cause of death in the country.[29] There is also historical precedent from other recent communicable disease outbreaks. Primary healthcare access was significantly impacted during prior infectious disease epidemics, such as Ebola virus disease, resulting in increases in morbidity and mortality.[30 31] Yet, whether and the extent to which similar effects will be seen during the COVID-19 epidemic is not known.

On 27th March, 2020, South Africa instituted a nationwide shelter-in-place order, termed in South Africa as a Level 5 lockdown. The level 5 order included closure of schools and all non-essential business, restrictions on public transport, and restrictions on movement. Restrictions on movement during the level 5 lockdown

For peer review only - http://bmjopen.bmj.com/site/about/guidelines.xhtml

specifically required that individuals remain in their place of residence, with the exceptions of "performing an essential service, obtaining an essential good or service, collecting a social grant, pension, or seeking emergency, life-saving or chronic medical attention." Over the following months, the restrictions gradually eased from level 5 down to 4 at the end of April and level 3 at the end of May, which corresponded with lifting restrictions on intra-province movement, preinitiation of public transportation, and allowed for reopening of schools and many business. [32 33] Because the healthcare sector was deemed an essential service throughout the entire lockdown period, no restrictions were placed on access to or delivery of healthcare services.

We sought to assess the impact of these lockdown orders in response to the COVID-19 epidemic in South Africa on access to basic healthcare services. We analysed data on clinic visitation at 11 ambulatory public health clinics in northern KwaZulu-Natal, collected routinely as part of a demographic health and surveillance system (HDSS) by the Africa Health Research Institute (AHRI). We hypothesized that there would be immediate and substantial reductions in clinic visitation after the

For peer review only - http://bmjopen.bmj.com/site/about/guidelines.xhtml

institution of the lockdown measure, and that this would pertain to routine clinical care such as immunizations, perinatal care, and chronic disease management in adults.

Methods

Study Setting

This analysis was conducted using data collected by the AHRI HDSS in the uMkhanyakude district of the KwaZulu-Natal Province. The HDSS comprises a complete census across a geographic area of approximately 850 km^2; it is a rural region with a single peri-urban centre, KwaMsane, a town of approximately 30,000 residents. The region ranks among the lowest nationwide in terms of health indicators and socioeconomic status.[34] Approximately 1 in 5 adult men and 2 in 5 adult women are living with HIV.[35] Tuberculosis incidence is among the highest in the world, and above the national average of 577 per 100,000 individuals when last measured in 2015.[36]

Data Collection

For peer review only - http://bmjopen.bmj.com/site/about/guidelines.xhtml

Since 2000 AHRI has collected data on births, deaths, migrations through thrice annual data collection encounters across a catchment area of 20,000 households (over 100,000 resident individuals).[34] In 2017, AHRI began placing clinic research assistants at each of the 11 government-run public health clinics in the area. These research staff operate in partnership with the Department of Health, but outside of the standard Health Management Information System (HMIS). For each person who presents to clinic, they collect demographic information and the self-reported reason(s) for the clinical visit. We link data between the HDSS and the clinic medical record system electronically using a unifying identification code for each resident of the catchment area. For this analysis, we included all individuals who presented to ambulatory clinic in the 11 regional clinics in the study catchment area during our observation period. There were no age or sex exclusions. AHRI holds memoranda of understanding with the Provincial and District Department of Health that permit extraction of health record data from primary care and hospital sites for linkage to the household surveillance dataset.

For peer review only

Study Design

We conducted an interrupted time series analysis to estimate changes in clinic visitation in rural KwaZulu-Natal from before to after the national lockdown implementation on 27th March 2020. To do so we fitted linear mixed effects regression models by restricted maximum likelihood with daily clinic visits as the primary outcome of interest. Our primary exposure of interest was time period, divided into four periods: 1) the pre-lockdown period starting 60-days prior to the initial level 5 lockdown until 27th March 2020; 2) the level 5 lockdown period from 28th March through 30th April 2020; 3) the level 4 lockdown level from 1st of May through 31st of May; and 4) the level 3 lockdown level from 1st of June through our data abstraction date (30th of June). For our primary outcome, we estimated the stepwise change in mean visits per clinic on the date of implementation lockdown level.[37] We included a fixed effect for day of the week, a random clinic-specific intercepts, and random clinic-specific slopes on time in our models. We excluded weekends because the study clinics do not provide non-urgent ambulatory care services on weekends. We excluded dates from observation when AHRI staff members who

For peer review only - http://bmjopen.bmj.com/site/about/guidelines.xhtml

perform data capture for the Clinic-link system were not working, including national holidays and staff trainings.

Our primary outcome of interest was the number of clinic visits for any reason per clinic. In secondary analyses, we stratified models by visit type restricted to: 1) child health visits (immunizations and growth monitoring); 2) antenatal care, postnatal care, and family planning; 3) HIV services (including antiretroviral therapy initiation, antiretroviral therapy continuation, and chronic care medical dispensing program visits); and 4) chronic care of non-communicable diseases (hypertension and diabetes). Clinic visits for more than one reason were treated as visits for both conditions. We also conducted stratified analyses by age category (<1, 1-5, 6-19, 20-45, and >45 years old) and by women and men aged 15 years or older.

We performed a number of secondary and sensitivity analyses to assess model validity and robustness of our findings. In addition to estimating stepwise changes after each lockdown order and change, we estimated trends in weekly visits during each period to determine whether immediate changes were sustained over each

For peer review only - http://bmjopen.bmj.com/site/about/guidelines.xhtml

period. We graphically depicted the residuals in the model to assess the normality assumption of our linear model structure. To check the robustness of model assumptions about changes from the pre-lockdown to the level 5 lockdown periods, we conducted multiple sensitivity analyses: 1) we specified a Poisson mixed effects regression model in place of a linear model; 2) we fitted linear and Poisson generalized estimating equation (GEE) models clustered by facility; 3) we specified an auto-regressive covariance structure in place of an exchangeable structure in the GEE models; and 4) to assess for the possibility of seasonal changes over multiple years, we constructed LOWESS plots to visually inspect clinical visitation trends over the same observation periods in 2020 versus 2018 and 2019 and fitted a difference-in-differences model that included year (2019 versus 2020) and time (characterized as pre-lockdown versus level 5 lockdown) to assess whether changes before and after the level 5 lockdown differed by calendar year.

Finally, we conducted an additional sensitivity analysis to assess for the possibility of in-migration into the HDSS catchment area during the lockdown period, which would potentially bias clinic visitation frequency upwards. To do so, we calculated annual

For peer review only

For peer review only - http://bmjopen.bmj.com/site/about/guidelines.xhtml

visitation frequency at the 11 area clinics for each individual in the dataset for the year prior to the lockdown. We then compared the median number of annual visits per individual in the pre- and post-lockdown periods, and the number of individuals with exactly one visit in the past year in the two periods. If significant in-migration did occur during the lockdown period, we would expect that the median number of annual visits per individual would decrease during the lockdown, whereas the number of individuals with one visit in the past 12 months would increase.

Three study investigators designed a statistical plan prior to all analyses (MJS, JDK, MJM). The initial analysis plan included fitting mixed effects models with random effects by clinic and inspecting trends in clinic visitation changes from the pre- to level 5 lockdown period for the overall cohort, and by visit sub-type. We also initially included plans for sensitivity analyses, including fitting of generalized estimating equations and additions of random slopes to our models as robustness checks. In response to reviewer requests and with updates to the lockdown characteristics from levels 5 to 4 to 3 during the review process, we conducted a number of post-hoc analyses, including construction of LOWESS plots and fitting additional models to

For peer review only

For peer review only - http://bmjopen.bmj.com/site/about/guidelines.xhtml

assess for seasonal trends in visitation by year, and fitting mixed effects Poisson models. All statistical analyses were conducted using Stata and R.

Patient and Public Involvement

This protocol was reviewed and approved by the AHRI Community Advisory Board, who contributed to the study design and selection of collection measures. Results of studies from the HDSS project are routinely shared with the community through public communications and road shows conducted by the AHRI Public Engagement Department. Final, all study protocols are reviewed and approved by the District and Provincial Department of Health, and AHRI holds memoranda of understanding with the Provincial and District Departments of Health that outline methods of extraction of health record data from primary care sites for linkage to the household surveillance dataset.

For peer review only

Ethical Approval

The protocol was reviewed and approved by the University of KwaZulu-Natal Biomedical Research Ethics Committee under reference BE290/16 and the KwaZulu Department of Health Research Committee.

Results

A total of 46,523 individuals made 89,476 clinic visits between 27th January – 30th June 2020 at the 11 area clinics (**Table 1**). Women and girls accounted for 67% (n=64,125) of visits. Approximately 9% of visits were made by individuals less than 1 year old (n=4,186), 1-5 year old (n=3,944), and 6-19 years old (n=4,460), respectively; whereas those 20-45 years accounted for 48% (n=22,231) and those over 46 the remaining 25% of visits (n=11,702). The most common reason for a clinic visit was ART follow-up care, comprising 43% of all visits (n=38,142), followed by visits for minor ailments (18%, n=16,204), child health (n=9,672, 11%) and hypertension (n=9,273, n=10%).

For peer review only

For peer review only - http://bmjopen.bmj.com/site/about/guidelines.xhtml

There was an average of 90.3 (95%CI 67.5, 113.2) clinic visits per day per clinic in the pre-lockdown period. We identified a non-significant drop in visits immediately following the start of the level 5 lockdown (-6.7 visits/clinic/day, 95%CI -16.5, 3.1). The small reduction seen after the level 5 period was reversed by a non-significant stepwise increase between the level 5 and level 4 periods (increase of 11.2 visits/clinic/day, 95%CI -0.5, 23.0), and persistent clinic visitation between the end of level 4 and the start of the level 3 period (increase of 1.2 visits/clinic/day, 95%CI -9.7, 12.1) (**Table 2, Figure 1**). There were no significant changes in trends over time in clinic visits/week in any of the pre- or post-implementation periods (**Supplemental Table 1**).

In contrast, child health visits suffered a 60% stepwise drop at the initiation of the level 5 lockdown (from 11.9 to 4.6 visits/day/clinic, mean change of -7.1 visits, 95%CI -9.0, -5.3), but remained steady during the transition between the level 5 and level 4 (-0.5 visits/clinic/day, 95%CI -2.7, 1.7), and again between the level 4 and level 3 lockdowns (-0.4 visits/clinic/day, 95%CI -2.5, 1.6) (**Table 2, Figure 1**). The reduction in child visits at the time of the level 5 lockdown occurred both among

For peer review only - http://bmjopen.bmj.com/site/about/guidelines.xhtml

children under 1 (mean decrease of -5.4 visits, 95%CI -7.0, -3.7) and those 1-5 years old (mean decrease of -5.5 visits, 95%CI -6.7, -4.4). We did detect fluctuating trends in child visitation during the lockdown periods, with increases of approximately 1 visit/clinic/week in levels 5 and 3, resulting in a similar number of mean visits/clinic/day one month into to the level 3 lockdown on 30th June as compared to just prior to the level 5 lockdown (11.2 visits/clinic/day [95%CI 7.4, 14.8] vs 11.9 visits/clinic/day [95%CI 8.6, 15.1], **Supplemental Table 1**).

In contrast to child health visits, HIV-related clinical visits for adults did not decrease between the pre-lockdown and level 5 lockdown period from 37.6 visits/clinic/day to 45.5 visits/clinic/day (mean change of 7.9 visits/clinic/day, 95%CI 2.1, 13.8), between the level 5 lockdown and level 4 lockdown period (increase of 11.1 clinic/visits/day, 95%CI 4.1, 18.0), or between the transition from the level 4 to level 3 (increase of 4.0 visits/clinic/day, 95%CI -2.5, 10.5) (**Table 2, Figure 1**). We similarly identified resilience in family planning visits over the observation period, increasing from 7.3 visits/clinic/day in the pre-implementation period to 7.8 visits/clinic/day after transition to level 5 (+0.5 visits/clinic/day, 95%CI -1.0, 2.0) to 8.9 clinic visits/day

For peer review only - http://bmjopen.bmj.com/site/about/guidelines.xhtml

after transition to level 4 (+1.1 visits/clinic/day, 95%CI -0.7, 3.0) and 11.0 visits/clinic/day after transition to level 3 (+2.0 visits/clinic/day, 95%CI 0.3, 3.7) for a 66% total increase from the pre-period. We did not detect changes in clinic visitation for chronic non-communicable diseases, or more broadly in clinic visitation by men or women 15 years or older.

Graphical depictions of residuals from our linear models demonstrated normally distributed residuals for the total visit, HIV, and child visit models, supporting the specification of a linear model to estimate trends in clinic visitation (**Supplemental Figure 1**). We found no evidence of changes in clinic visitation during this same period in 2019 to suggest seasonal effects, either graphically in LOWESS plots, or in difference-in-differences in models including both 2019 and 2020 with pre and post-lockdown periods (**Table 3**). Results were robust to modelling assumptions in the sensitivity analyses (Supplemental **Table 3**).

Finally, we did not detect evidence of meaningful in-migration during the lockdown period. The median number of visits in the past year per individual attending the

For peer review only - http://bmjopen.bmj.com/site/about/guidelines.xhtml

clinic slightly increased from the pre-lockdown period to the lockdown period (mean 5.8 [SD 0.02] vs. 5.9 [SD 0.03], P<0.001). This pattern was similar among people attending clinic for HIV-specific visits (mean 6.5 [SD 0.02] vs mean 6.6 [SD 0.04], P=0.01). The number of people with exactly one visit in the past year also did not meaningfully increase during the observation period with 1,960 (February), 2,115 (March), 1,573 (April), 1,893 (May), and 1,986 (June) visits made by individuals with exactly one annual clinic visit over the prior 12 months.

Discussion

We found evidence of a significant drop in visits for childcare alongside sustained visitation in HIV and adult ambulatory clinic utilization in a rural area of South Africa during the national lockdown for the COVID-19 epidemic. Notably, visits for chronic disease, such as hypertension and diabetes, perinatal care and family planning remained reasonably constant or modestly increased. However, child health visits for immunizations and growth monitoring dropped immediately by over 50% after the start of the lockdown. With gradual increases over time during level 5 and level 3 lockdown, child visits largely returned to pre-lockdown levels in June, approximately

For peer review only - http://bmjopen.bmj.com/site/about/guidelines.xhtml

3 months after the lockdown began. We noted an estimated 20% increase in clinic visits for HIV immediately after the lockdown and suspect this might have reflected an urgency to collect medications prior to an anticipated interruption in clinic access or medication availability and/or national programmatic efforts to accelerate transitions to a new first-line regimen.[38] These results demonstrate concerning trends about reductions in preventative child care during the lockdown period. However, they also appeared to disprove our hypothesis about clinic visitation in adults, and potentially demonstrate a resilience in the healthcare sector during a period of concern for access to chronic and essential basic health services.

The key demographic population in our study that experienced significant drops in clinic visitation was children. Child health visits appeared to have modestly rebounded during the lockdown, and eventually return to their pre-lockdown state. Although these data do not suggest the cause of reduced visitation in children, multiple possible factors might be considered. We hypothesize that limited options for childcare for families with multiple children might prevent caregivers from being able to bring individual children to clinic. Moreover, in contrast to, for example, HIV

For peer review only - http://bmjopen.bmj.com/site/about/guidelines.xhtml

wellcare visits, well child visits rarely involve medication refills so might be prioritized lower for families. Whatever the cause, our findings are in keeping with data from elsewhere. In Hangzhou, China, pediatric healthcare visits dropped by nearly 75% during the peak of the epidemic and lockdown periods.[39] In the U.S. vaccination rates in children substantially declined after a national emergency was declared in response to the COVID-19 epidemic.[40] Modeling analyses using Lived Saves Tool (LiST) have suggested that a 15% reduction in maternal and child health coverage could result in over 250,000 additional deaths.[23] The World Health Organization has also projected significant increases in deaths due to malaria in children under 5 in endemic regions with disruptions in malaria care and insecticide treated bednet distribution.[41] Although empiric data on healthcare access in South Africa remain scarce, work to date has suggested significant reductions in tuberculosis testing in laboratory databases, and reports of interruptions in care in community-based surveys.[28 42] Previous disease epidemics in sub-Saharan Africa have also been associated with lapses in primary care access, and drops in facility based births and child healthcare access.[30 31 43 44] Consequently, future work should investigate the impacts of even modest drops in vaccination rates and child health outcomes, to

For peer review only - http://bmjopen.bmj.com/site/about/guidelines.xhtml

better assess whether the drop we identified resulted in longer term health effects, and whether catch-up vaccination campaigns might help limit the fallout of such interruptions.[45]

Maintaining healthcare access during the epidemic requires a careful balance of primary healthcare provision and protection of vulnerable populations from COVID-19 infection. In other settings, there have been multiple reports of late and severe presentations to care for non-COVID-19 conditions, putatively due to decreased access to care or fear of nosocomial infection at healthcare facilities.[46-48] At the time of our data abstraction at the end of June during the level 3 lockdown period, fewer than 200 cases of COVID-19 infection had been reported in uMkhanyakude District.[49] Thus, our data largely reflect impacts of lockdown measures prior to an epidemic with significant local transmission. Clinics in this district instituted symptom screening at the entryway to clinics, with referral of individuals meeting criteria for persons under investigation to regional COVID-19 testing centres. Future work should revisit the impacts of the epidemic itself on access to primary healthcare.

For peer review only - http://bmjopen.bmj.com/site/about/guidelines.xhtml

The COVID-19 epidemic has also led to calls for decentralized care to minimize exposure for high-risk populations,[50] including those with chronic non-communicable disease, HIV, a history of tuberculosis-related lung disease, and those of older-ages. The lockdown was instituted rapidly in South Africa, before substantial decentralized care systems could be put in place. However, an important unanswered question is how such programs will affect access to care and epidemic transmission in high-risk populations, including the elderly and those with immunosuppressing conditions.

Our study should be interpreted within the context of the relatively short period (3 months) of the lockdown in South Africa. As a result, we are not yet able to assess longer-term repercussions from disruptions to income or from the epidemic itself, or longer-term effects of lapses in primary care and vaccination on health outcomes, and our results should not be generalized over longer time horizons. It is expected that economic barriers to healthcare utilization will increase as the epidemic's effects persist over time, including secondary effects from non-pharmaceutical interventions. These effects are likely to fall most heavily on those in the informal economy.[51] South Africa has taken steps to increase social support to counteract economic

For peer review only - http://bmjopen.bmj.com/site/about/guidelines.xhtml

disruption from the epidemic and control measures.[52] Mitigating longer-term consequences may require governments and development partners to increase access to employment and other social support services during the epidemic.

Our study had multiple strengths. First, our data collection procedures are led by research staff who remained in place during the lockdown period, so these data are not affected by barriers to data collection (e.g., interruptions in staff transportation or workplace access). This is important, since many routine health information systems could be expected to suffer lapses during external shocks to the healthcare system. Second, our study was able to access data collected across 11 clinical centres within a large HDSS, which provided significant power to detect even small interruptions to health care access. A key potential limitation to our study is that it is predicated on the assumption that there were no other external factors that would have caused interruptions to the health care system on or after 27th March 2020 (e.g., power outage, inclement weather).We are unaware of any such shock and believe this to be a minor risk. We saw no evidence of a seasonal effect after comparing our results to similar time periods in 2019. Our analysis should also be interpreted within the

For peer review only - http://bmjopen.bmj.com/site/about/guidelines.xhtml

context of our study area – one with approximately 200 reported cases of COVID-19 at the time of the analysis, but in a nation with a large epidemic (approximately 200,000 cases as of early July) with established local transmission in other areas.

In summary, we report a reduction in child wellcare visitation but resilience of the adult ambulatory health care system during the early COVID-19 epidemic and lockdown period in rural South Africa. Future work should establish if these trends are maintained, and particularly monitor access to childcare and immunizations as a result of the trends reported in this study. Finally, in rural South Africa and similar areas, efforts to balance ongoing provision of essential preventative and chronic healthcare services might be needed to ensure healthcare access remains intact while preventing nosocomial spread of COVID-19 among high-risk populations.

For peer review only

For peer review only - http://bmjopen.bmj.com/site/about/guidelines.xhtml

Table 1. Ambulatory clinic visits at 11 region clinics in rural KwaZulu Natal during 27 January 2020 – 30 June 2020 by sex and age and clinic visit type

	**Total* n (%)**	**Male n (%)**	**Female n (%)**	**<1 year n (%)**	**1-5 years n (%)**	**6-19 years n (%)**	**20-45 years n (%)**	**>45 years n (%)**
Total visits	89,476 (100%)	25,318 (28.3%)	64,125 (71.7%)	7,734 (8.6%)	5,646 (6.3%)	7,429 (8.3%)	43,116 (48.2%)	25,551 (28.6%)
Child health	9,672 (10.8%)	4,808 (49.7%)	4,846 (50.1%)	6,712 (69.4%)	2,750 (28.4%)			
PNC and FP[a]	7,879 (8.8%)	13 (0.2%)	7,866 (99.8%)	9 (0.1%)	2 (0.0%)	1,266 (16.1%)	6,570 (83.4%)	32 (0.4%)
HIV visit[b]	42,585 (47.6%)	11,152 (26.2%)	31,425 (73.8%)	44 (0.1%)	215 (0.5%)	2,549 (6.0%)	27,218 (63.9%)	12,559 (29.5%)
Chronic care[c]	10,052 (11.2%)	2,143 (21.3%)	7,909 (78.7%)	1 (0.0%)	2 (0.0%)	11 (0.1%)	647 (6.4%)	9,391 (93.4%)
Minor ailment	2,738 (3.1%)	988 (36.1%)	1,750 (63.9%)	133 (4.9%)	471 (17.2%)	384 (14.0%)	1,084 (39.6%)	666 (24.3%)
All other visits	18,043 (20.2%)	6,378 (35.4%)	11,658 (64.6%)	842 (4.7%)	2,222 (12.3%)	3,112 (17.3%)	8,390 (46.5%)	3,477 (19.3%)

*Visit types are not mutually exclusive so column totals may exceed 100%
[a]PNC and FP: Perinatal care and family planning; visits for, antenatal care, prenatal care, and/or family planning
[b]HIV visits: visits for HIV testing, antiretroviral therapy initiation, antiretroviral therapy continuation, or pharmacy pick-up
[c]Chronic care: visits for hypertension and/or diabetes

For peer review only

For peer review only - http://bmjopen.bmj.com/site/about/guidelines.xhtml

Table 2. Mixed effects regression model results demonstrating changes in mean visits/clinic, by visit type and demographic strata, in the pre-lockdown period, and after each transition to level 5, level 4, and level 3 lockdowns in uMkhanyakude District, KwaZulu-Natal South Africa.

**Model**	**Mean daily visits per clinic at time of lockdown period (intercept)**	**Stepwise change in visits/clinic/day at start of level 5 lockdown**	***P*-value**	**Stepwise change in visits/clinic/day at start of level 4 lockdown**	***P*-value**	**Stepwise change in clinic visits/clinic/day at start of level 3 lockdown**	***P*-value**
Total visits	90.3 (67.1, 113.5)	-6.7 (-16.4, 3.0)	0.18	11.3 (-0.3, 22.9)	0.06	1.2 (-9.6, 12.0)	0.83
Child health[a]	11.9 (8.6, 15.1)	-7.1 (-8.9, -5.3)	<0.001	-0.5 (-2.6, 1.6)	0.65	-0.4 (-2.4, 1.6)	0.67
PNC and FP[b]	7.3 (4.2, 10.3)	0.5 (-1.0, 2.0)	0.51	1.1 (-0.7, 2.9)	0.22	2.0 (0.3, 3.7)	0.02
HIV visits[c]	37.5 (24.4, 50.7)	8.0 (2.3, 13.7)	0.01	11.0 (4.2, 17.8)	0.001	4.0 (-2.3, 10.3)	0.22
Chronic care[d]	9.5 (7.1, 11.8)	-0.3 (-1.9, 1.3)	0.70	-0.15 (-2.1, 1.8)	0.88	0.4 (-1.4, 2.2)	0.65
Men ≥ 15	15.4 (10.8, 19.9)	1.5 (-0.7, 3.6)	0.17	2.3 (-0.2, 4.9)	0.07	0.8 (-1.5, 3.2)	0.50
Women ≥ 15	52.9 (38.1, 67.7)	3.2 (-3.3, 9.6)	0.33	9.6 (1.9, 17.3)	0.01	2.1 (-5.1, 9.3)	0.57
Age <1	10.6 (7.6, 13.5)	-5.3 (-6.9, -3.7)	<0.001	0.2 (-1.6, 2.1)	0.83	-0.4 (-2.1, 1.4)	0.67
Age 1-5	8.9 (7.4, 10.5)	-5.6 (-6.7, -4.4)	<0.001	-0.2 (-1.5, 1.1)	0.80	-0.8 (-2.1, 0.4)	0.18
Age 6-19	8.0 (6.2, 9.8)	-0.7 (-1.9, 0.6)	0.29	1.5 (0.0, 3.0)	0.05	-0.1 (-1.5, 1.3)	0.91
Age 20-45	39.3 (26.3, 52.2)	4.4 (-0.7, 9.5)	0.09	7.5 (1.4, 13.6)	0.02	2.2 (-3.5, 7.8)	0.45
Age >45	25.2 (19.2, 31.3)	0.4 (-3.0, 3.7)	0.83	2.9 (-1.1, 6.9)	0.15	0.5 (-3.2, 4.3)	0.78

[a]Child health: visits for immunizations and growth monitoring
[b]PNC and FP: perinatal care and family planning; visits for, antenatal care, prenatal care, and/or family planning
[c]HIV visits: visits for HIV testing, antiretroviral therapy initiation, antiretroviral therapy continuation, or pharmacy pick-up
[d]Chronic care: clinical visits for hypertension and/or diabetes

For peer review only - http://bmjopen.bmj.com/site/about/guidelines.xhtml

Table 3. Sensitivity analyses, demonstrating results of the main regression model and alternate models.

All Visits	Mean daily visits per clinic at time of lockdown period	Stepwise change in clinic visits/day at start of level 5 lockdown	*P*-value
Primary model	90.3 (67.1, 113.5)	-6.7 (-16.4, 3.0)	0.18
Poisson[a] mixed effects model	92.0 (59.3, 124.7)	-6.9 (-11.0, -2.8)	0.001
Linear GEE (exchangeable correlation matrix)	89.2 (67.0, 111.4)	-6.4 (-16.8, 4.08)	0.23
Poisson GEE[a] (exchangeable correlation matrix)	89.2 (84.7, 93.6)	-6.6 (-8.7, -4.5)	<0.001
Linear GEE (autoregressive correlation matrix)	90.2 (73.2, 107.2)	-5.4 (-27.4, 16.6)	0.63
Poisson GEE[a] (autoregressive correlation matrix)	88.4 (85.0, 91.9)	-5.0 (-9.4, -0.6)	0.03
Difference-in-Differences[b]	96.3 (63.6, 129.0)	3.4 (-5.5, 12.4)	0.45
**Childcare Visits**			
Primary model	11.9 (8.6, 15.1)	-7.1 (-8.9, -5.3)	<0.001
Poisson[a] mixed effects model	12.3 (7.1, 17.5)	-7.7 (-11.1, -4.4)	<0.001
Linear GEE (exchangeable correlation matrix)	11.8 (8.6, 15.0)	-7.1 (-9.0, -5.2)	<0.001
Poisson GEE[a] (exchangeable correlation matrix)	12.0 (10.5, 13.5)	-7.5 (-8.4, -6.6)	<0.001
Linear GEE (autoregressive correlation matrix)	11.9 (9.5, 14.4)	-6.4 (-9.9, -2.9)	<0.001
Poisson GEE[a] (autoregressive correlation matrix)	11.9 (10.7, 13.1)	-6.7 (-8.1, -5.4)	<0.001
Difference-in-Differences[b]	11.8 (8.0, 15.7)	-4.0 (-5.5, -2.5)	<0.001
**HIV Visits**			

For peer review only - http://bmjopen.bmj.com/site/about/guidelines.xhtml

Primary model	37.5 (24.4, 50.7)	8.0 (2.3, 13.7)	0.01
Poisson[a] mixed effects model	39.2 (22.7, 55.8)	9.0 (4.5, 13.5)	<0.001
Linear GEE (exchangeable correlation matrix)	37.7 (25.3, 50.1)	8.1 (2.2, 14.0)	0.007
Poisson GEE[a] (exchangeable correlation matrix)	37.7 (34.8, 40.6)	8.7 (7.2, 10.3)	<0.001
Linear GEE (autoregressive correlation matrix)	38.9 (29.3, 48.5)	6.1 (-6.2, 18.5)	0.33
Poisson GEE[a] (autoregressive correlation matrix)	37.9 (35.7, 40.1)	5.7 (2.6, 8.8)	0.002
Difference-in-Differences[b]	43.6 (25.1, 62.1)	4.8 (-0.5, 10.1)	0.08

GEE: generalized estimating equations

[a]Poisson GEE results are presented as predictive margins and marginal effects so they represent changes on the same additive scale as the linear models.

[b]Difference-in-differences estimates are estimated as the mean of the level 5 lockdown period minus the mean of the pre-lockdown period, comparing 2020 to 2019. Estimates are based on a period-by-year interaction term fit via linear mixed models.

For peer review only

Figure Legends

Figure 1. Ambulatory clinic visitation before and after the nationwide lockdown in South Africa at eleven outpatient clinics in rural uMkhanyakude District, KwaZulu-Natal South Africa. Scatterplots represent mean clinic visitation at each clinic on weekdays during the observation period. The black fit line represents the mean visitation across all clinics estimated by post-regression margins from a linear regression model, with a regression discontinuity coefficient at the date of the lockdown (27th March 2020, red line). Gray bars represent 95% confidence intervals. The dotted blue line represents the geometic mean of the number of visits across all clinics on each day.

Supplemental Figure 1. Plots of residuals around estimated mean from linear mixed effects regression models of clinic visitation for total visits (A), child health visits (B), and HIV visits (C)

Supplemental Figure 2. Scatter and LOWESS plots with smoothed regression functions demonstrating clinic visits per day in the study observation period during 2019 and 2020 for all visits (A), child health visits (B), and HIV adults (C).

For peer review only

Author contributions

Contributor Role	Role Definition	
Conceptualization	Ideas; formulation or evolution of overarching research goals and aims.	MJS
Data Curation	Management activities to annotate (produce metadata), scrub data and maintain research data (including software code, where it is necessary for interpreting the data itself) for initial use and later reuse.	DG, KH
Formal Analysis	Application of statistical, mathematical, computational, or other formal techniques to analyze or synthesize study data.	MJS, MDM, JDK, GH
Funding Acquisition	Acquisition of the financial support for the project leading to this publication.	KH, WH
Investigation	Conducting a research and investigation process, specifically performing the experiments, or data/evidence collection.	KH, MJS, CI MS
Methodology	Development or design of methodology; creation of models.	KH, MJS
Project Administration	Management and coordination responsibility for the research activity planning and execution.	KH, JS, MS, NN, NM
Resources	Provision of study materials, reagents, materials, patients, laboratory samples, animals, instrumentation, computing resources, or other analysis tools.	KH
Software	Programming, software development; designing computer programs; implementation of the computer code and supporting algorithms; testing of existing code components.	KH, DG
Supervision	Oversight and leadership responsibility for the research activity planning and execution, including mentorship external to the core team.	KH, MS, TM, PG, SD

For peer review only - http://bmjopen.bmj.com/site/about/guidelines.xhtml

Contributor Role	Role Definition	
Validation	Verification, whether as a part of the activity or separate, of the overall replication/reproducibility of results/experiments and other research outputs.	KH
Visualization	Preparation, creation and/or presentation of the published work, specifically visualization/data presentation.	KH, MJS
Writing – Original Draft Preparation	Creation and/or presentation of the published work, specifically writing the initial draft (including substantive translation).	MJS, JDK
Writing – Review & Editing	Preparation, creation and/or presentation of the published work by those from the original research group, specifically critical review, commentary or revision – including pre- or post-publication stages.	KH, MJS, MDM, JS, DG, GH, EW, JDK, CI, MS, NN, TM, PG, SD, WH, NM

Transparency declaration

MJS affirms the manuscript is an honest, accurate, and transparent account of the study being reported; that no important aspects of the study have been omitted; and that any discrepancies from the study as originally planned (and, if relevant, registered) have been explained.

Data sharing

Data from the AHRI HDSS are publicly available upon request to the AHRI research repository which can be made here: https://data.africacentre.ac.za/index.php/auth/login/?destination=

Competing interests and conflicts of interest

All authors have completed the ICMJE uniform disclosure form and declare no financial relationships with any organisations that might have an interest in the submitted work in the previous three years and no other relationships or activities that could appear to have influenced the submitted work.

Funding information

Wellcome Trust. 082384/Z/07/Z/. Willem Hanekom

For peer review only - http://bmjopen.bmj.com/site/about/guidelines.xhtml

Wellcome Trust and Royal Society. 210479/Z/18/Z. Guy Harling
Shahmanesh is supported by National Institutes of Health under award number 5R01MH114560-03, Bill & Melinda Gates Foundation, Grant Number OPP1136774 and OPP1171600.
National Human Genome Research Institute of the National Institutes of Health (NIH) under Award Number U24HG006941, South African Medical Research Council (MRC-RFA-UFSP-01-2013/UKZN HIVEPI) and the South African Department of Science and Innovation (DSI).
Mark J. Siedner is supported by the National Institutes of Health (R01 AI124718, R01 AG059504).

Acknowledgements

We thank the outstanding field staff at the Africa Health Research Institute for their work in conducting the demographic health survey field work, as well as the community and the study participants for their time and efforts. The population HIV surveillance is a node of the South African Population Research Infrastructure Network (SAPRIN) and funded by the South African Department of Science and Innovation and hosted by the South African Medical Research Council. Additional thanks to Zahra Reynolds for her assistance in organizing elements of the manuscript for submission.

License
The Corresponding Author has the right to grant on behalf of all authors and does grant on behalf of all authors, an exclusive license (or non exclusive for government employees) on a worldwide basis to the BMJ Publishing Group Ltd to permit this article (if accepted) to be published in BMJ editions and any other BMJPGL products and sublicences such use and exploit all subsidiary rights, as set out in our license.

For peer review only

References

1. World Health Organization. Coronavirus disease (COVID-2019) situation reports. Available at: https://www.who.int/emergencies/diseases/novel-coronavirus-2019/situation-reports Accessed 2 May 2020.
2. Johnstone-Robertson SP, Mark D, Morrow C, et al. Social mixing patterns within a South African township community: implications for respiratory disease transmission and control. *Am J Epidemiol* 2011;174(11):1246-55. doi: 10.1093/aje/kwr251 [published Online First: 2011/11/11]
3. le Polain de Waroux O, Cohuet S, Ndazima D, et al. Characteristics of human encounters and social mixing patterns relevant to infectious diseases spread by close contact: a survey in Southwest Uganda. *BMC Infect Dis* 2018;18(1):172. doi: 10.1186/s12879-018-3073-1 [published Online First: 2018/04/13]
4. House T, Keeling MJ. Household structure and infectious disease transmission. *Epidemiol Infect* 2009;137(5):654-61. doi: 10.1017/S0950268808001416 [published Online First: 2008/10/09]
5. Nkengasong JN, Mankoula W. Looming threat of COVID-19 infection in Africa: act collectively, and fast. *Lancet* 2020;395(10227):841-42. doi: 10.1016/S0140-6736(20)30464-5 [published Online First: 2020/03/03]
6. Gilbert M, Pullano G, Pinotti F, et al. Preparedness and vulnerability of African countries against importations of COVID-19: a modelling study. *Lancet* 2020;395(10227):871-77. doi: 10.1016/S0140-6736(20)30411-6 [published Online First: 2020/02/24]
7. Kapata N, Ihekweazu C, Ntoumi F, et al. Is Africa prepared for tackling the COVID-19 (SARS-CoV-2) epidemic. Lessons from past outbreaks, ongoing pan-African public health efforts, and implications for the future. *Int J Infect Dis* 2020;93:233-36. doi: 10.1016/j.ijid.2020.02.049 [published Online First: 2020/03/03]
8. Adhikari NK, Fowler RA, Bhagwanjee S, et al. Critical care and the global burden of critical illness in adults. *Lancet* 2010;376(9749):1339-46. doi: 10.1016/S0140-6736(10)60446-1 [published Online First: 2010/10/12]
9. Sands P, Mundaca-Shah C, Dzau VJ. The Neglected Dimension of Global Security--A Framework for Countering Infectious-Disease Crises. *N Engl J Med* 2016;374(13):1281-7. doi: 10.1056/NEJMsr1600236 [published Online First: 2016/01/14]
10. Fong MW, Gao H, Wong JY, et al. Nonpharmaceutical Measures for Pandemic Influenza in Nonhealthcare Settings-Social Distancing Measures. *Emerg Infect Dis* 2020;26(5):976-84. doi: 10.3201/eid2605.190995 [published Online First: 2020/02/07]
11. Markel H, Lipman HB, Navarro JA, et al. Nonpharmaceutical interventions implemented by US cities during the 1918-1919 influenza pandemic. *Jama* 2007;298(6):644-54. doi: 10.1001/jama.298.6.644 [published Online First: 2007/08/09]
12. Peak CM, Kahn R, Grad YH, et al. 2020 doi: 10.1101/2020.03.05.20031088
13. Ferguson NM, Cummings DA, Cauchemez S, et al. Strategies for containing an emerging influenza pandemic in Southeast Asia. *Nature* 2005;437(7056):209-14. doi: 10.1038/nature04017 [published Online First: 2005/08/05]
14. Bayham J, Fenichel EP. Impact of school closures for COVID-19 on the US health-care workforce and net mortality: a modelling study. *Lancet Public Health* 2020 doi: 10.1016/S2468-2667(20)30082-7 [published Online First: 2020/04/07]
15. Keogh-Brown MR, Wren-Lewis S, Edmunds WJ, et al. The possible macroeconomic impact on the UK of an influenza pandemic. *Health Economics* 2010;19(11):1345-60. doi: 10.1002/hec.1554
16. Smith RD, Keogh-Brown MR, Barnett T, et al. The economy-wide impact of pandemic influenza on the UK: a computable general equilibrium modelling experiment. *BMJ* 2009;339:b4571. doi: 10.1136/bmj.b4571 [published Online First: 2009/11/21]

For peer review only - http://bmjopen.bmj.com/site/about/guidelines.xhtml

17. Nicola M, Alsafi Z, Sohrabi C, et al. The socio-economic implications of the coronavirus pandemic (COVID-19): A review. *International Journal of Surgery* 2020;78:185-93. doi: 10.1016/j.ijsu.2020.04.018
18. Mongey S, Pilossoph L, Weinberg A. 2020 doi: 10.3386/w27085
19. Quaife M, van Zandvoort K, Gimma A, et al. 2020 doi: 10.1101/2020.06.06.20122689
20. COVID-19 in Africa: Protecting Lives and Economies. Economic Commission for Africa., 2020.
21. Dahab M, van Zandvoort K, Flasche S, et al. COVID-19 control in low-income settings and displaced populations: what can realistically be done? Pre-print. Available at: https://www.lshtm.ac.uk/newsevents/news/2020/covid-19-control-low-income-settings-and-displaced-populations-what-can#.XnpKCXF93Fc.email. Accessed: 1 May 2020. 2020
22. Greenstone M, Nigam V. Does Social Distancing Matter? *SSRN Electronic Journal* 2020 doi: 10.2139/ssrn.3561244
23. Roberton T, Carter ED, Chou VB, et al. Early Estimates of the Indirect Effects of the Coronavirus Pandemic on Maternal and Child Mortality in Low- and Middle-Income Countries. *SSRN Electronic Journal* 2020 doi: 10.2139/ssrn.3576549
24. The unanticipated costs of COVID-19 to South Africa's quadruple disease burden. Academy of Science of South Africa. 2020 [Available from: https://www.assaf.org.za/files/2020/ASSAf%20Health%20Committee%20Statement%20-%202%20July%202020.pdf accessed 15 July 2020.
25. Abdool Karim Q, Abdool Karim SS. COVID-19 affects HIV and tuberculosis care. *Science* 2020;369(6502):366-68. doi: 10.1126/science.abd1072 [published Online First: 2020/07/25]
26. UNAIDS. The impact of the COVID-19 response on the supply chain, availability and cost of generic antiretroviral medicines for HIV in low- and middle-income countries. Available at: https://www.unaids.org/en/resources/documents/2020/covid19-supply-chain-availability-cost-generic-arv; Accessed 25 July 2020. 2020
27. Jewell BL, Smith JA, Hallett TB. 2020 doi: 10.1101/2020.04.22.20075861
28. Madhi SA, Gray GE, Ismail N, et al. COVID-19 lockdowns in low- and middle-income countries: Success against COVID-19 at the price of greater costs. *South African Medical Jounral* 2020;110(8):724-26.
29. Mortality and causees of death in South Africa: Findings from death notification. Statistics South Africa. Available at: http://www.statssa.gov.za/publications/P03093/P030932017.pdf. Accessed 15th July 2020. 2020
30. Delamou A, Ayadi AME, Sidibe S, et al. Effect of Ebola virus disease on maternal and child health services in Guinea: a retrospective observational cohort study. *The Lancet Global Health* 2017;5(4):e448-e57. doi: 10.1016/s2214-109x(17)30078-5
31. Ly J, Sathananthan V, Griffiths T, et al. Facility-Based Delivery during the Ebola Virus Disease Epidemic in Rural Liberia: Analysis from a Cross-Sectional, Population-Based Household Survey. *PLOS Medicine* 2016;13(8) doi: 10.1371/journal.pmed.1002096
32. Republic of South Africa. Disaster Management Act: Regulations to address, prevent and combat the spread of Coronavirus-19 (COVID-19): Amendment. Available at: https://www.gov.za/documents/disaster-management-act-regulations-address-prevent-and-combat-spread-coronavirus-covid-19. Accessed 1 May 2020.
33. Regulations and Guidelines - Coronavirus COVID-19. South African Government. [Available from: https://www.gov.za/coronavirus/guidelines accessed 10th July 2020.
34. Tanser F, Hosegood V, Barnighausen T, et al. Cohort Profile: Africa Centre Demographic Information System (ACDIS) and population-based HIV survey. *Int J Epidemiol* 2008;37(5):956-62. doi: 10.1093/ije/dym211 [published Online First: 2007/11/14]
35. Vandormael A, Akullian A, Siedner M, et al. Declines in HIV incidence among men and women in a South African population-based cohort. *Nat Commun* 2019;10(1):5482. doi: 10.1038/s41467-019-13473-y [published Online First: 2019/12/04]

For peer review only - http://bmjopen.bmj.com/site/about/guidelines.xhtml

36. Massyn N, Peer N, English R, et al. District Health Barometer 2015/2016. Health Systems Trust. Available at: https://www.hst.org.za/publications/Pages/-District-Health-Barometer-201516.aspx; Accessed 29 April 2020. 2016
37. Kontopantelis E, Doran T, Springate DA, et al. Regression based quasi-experimental approach when randomisation is not an option: interrupted time series analysis. *Bmj* 2015;350(jun09 5):h2750-h50. doi: 10.1136/bmj.h2750
38. Dorward J, Lessells R, Drain PK, et al. Dolutegravir for first-line antiretroviral therapy in low-income and middle-income countries: uncertainties and opportunities for implementation and research. *The Lancet HIV* 2018;5(7):e400-e04. doi: 10.1016/s2352-3018(18)30093-6
39. Li H, Yu G, Duan H, et al. Changes in Children's Healthcare Visits During Coronavirus Disease-2019 Pandemic in Hangzhou, China. *The Journal of Pediatrics* 2020 doi: 10.1016/j.jpeds.2020.05.013
40. Santoli JM, Lindley MC, DeSilva MB, et al. Effects of the COVID-19 Pandemic on Routine Pediatric Vaccine Ordering and Administration — United States, 2020. *MMWR Morbidity and Mortality Weekly Report* 2020;69(19) doi: 10.15585/mmwr.mm6919e2
41. World Health Organization. The potential impact of health service disruptions on the burden of malaria: a modelling analysis for countries in sub-Saharan Africa. Available at: https://apps.who.int/iris/bitstream/handle/10665/331845/9789240004641-eng.pdf Accessed 4 May 2020.
42. Burger R, Nknonki L, Rensburg R, et al. Examining the unintended health consequences of the COVID-19 pandemic in South Africa. Wave 1 National Income Dynamics Study (NIDS) - Coronavirus Rapid Mobile Survey (CRAM). Available at: https://cramsurvey.org/wp-content/uploads/2020/07/Burger-examining-the-unintended-health-consequences.pdf; Accessed 31 July 2020. 2020
43. Takahashi S, Metcalf CJ, Ferrari MJ, et al. Reduced vaccination and the risk of measles and other childhood infections post-Ebola. *Science* 2015;347(6227):1240-2. doi: 10.1126/science.aaa3438 [published Online First: 2015/03/15]
44. Elston JWT, Moosa AJ, Moses F, et al. Impact of the Ebola outbreak on health systems and population health in Sierra Leone. *Journal of Public Health* 2015 doi: 10.1093/pubmed/fdv158
45. Nelson R. COVID-19 disrupts vaccine delivery. *Lancet Infect Dis* 2020 doi: 10.1016/S1473-3099(20)30304-2 [published Online First: 2020/04/21]
46. Tam CF, Cheung KS, Lam S, et al. Impact of Coronavirus Disease 2019 (COVID-19) Outbreak on ST-Segment-Elevation Myocardial Infarction Care in Hong Kong, China. *Circ Cardiovasc Qual Outcomes* 2020;13(4):e006631. doi: 10.1161/CIRCOUTCOMES.120.006631 [published Online First: 2020/03/18]
47. Moroni F, Gramegna M, Ajello S, et al. Collateral damage: medical care avoidance behavior among patients with acute coronary syndrome during the COVID-19 pandemic. *JACC: Case Reports* 2020 doi: 10.1016/j.jaccas.2020.04.010
48. Lazzerini M, Barbi E, Apicella A, et al. Delayed access or provision of care in Italy resulting from fear of COVID-19. *The Lancet Child & Adolescent Health* 2020;4(5):e10-e11. doi: 10.1016/s2352-4642(20)30108-5
49. Premier Sihle Zikalala's Media Briefing & Update on Covid19 in the Province Since the start of Level 4 Lockdown. Available at http://www.kznonline.gov.za/index.php/component/k2/item/1207-premier-sihle-zikalala-s-media-briefing-update-on-covid19-in-the-province-since-the-start-of-level-4-lockdown. Accessed 11 May 2020.
50. Wilkinson L, Grimsrud A. The time is now: expedited HIV differentiated service delivery during the COVID-19 pandemic. *Journal of the International AIDS Society* 2020;23(5) doi: 10.1002/jia2.25503

For peer review only - http://bmjopen.bmj.com/site/about/guidelines.xhtml

51. International Labour Organization. The The impact of the COVID-19 on the informal economy in Africa and the related policy responses. Available at: https://www.ilo.org/wcmsp5/groups/public/---africa/---ro-abidjan/documents/briefingnote/wcms_741864.pdf Accessed 3 May 2020.
52. Mogotsi B, Bearak M. South Africa is hunting down coronavirus with thousands of health workers. April 21, 2020. *Washington Post* 2020.

For peer review only

For peer review only - http://bmjopen.bmj.com/site/about/guidelines.xhtml

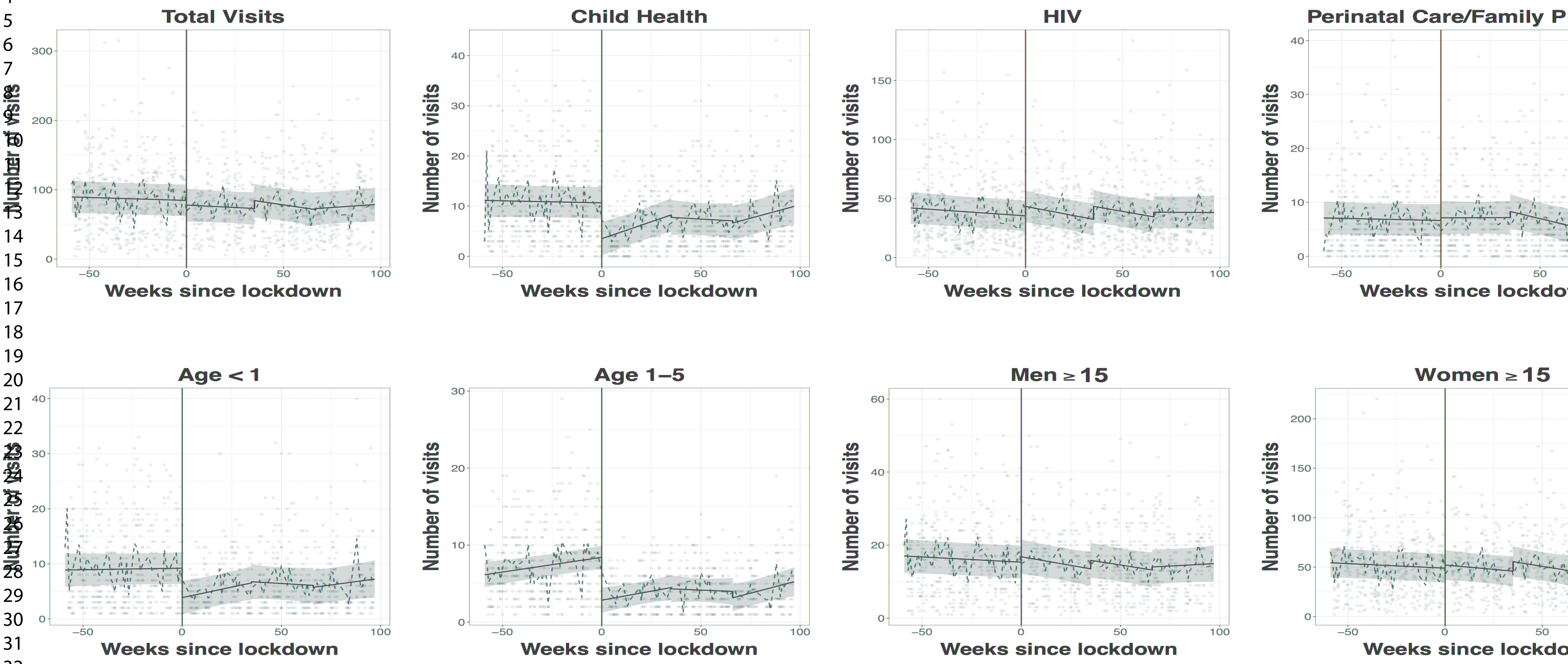
Total Visits
Child Health
HIV
Perinatal Care/Family P
Age < 1
Age 1–5
Men ≥ 15
Women ≥ 15
Number of visits
Weeks since lockdown

Supplemental Table 1. Mixed effects regression model results demonstrating changes in mean clinic visits by week, by visit type and demographic strata, in the pre- and post-lockdown period in uMkhanyakude District, KwaZulu-Natal South Africa.

Model	Weekly change in clinic visits/week during pre-lockdown period	P-value	Change in weekly change in clinic visits/week during level 5 lockdown period	*P*-value	Change in weekly change in clinic visits/week during level 4 lockdown period	*P*-value	Change in weekly change in clinic visits/week during level 3 lockdown period)	*P*-value
Total visits	-0.6 (-1.8, 0.6)	0.33	-0.4 (-3.3, 2.5)	0.80	-1.9 (-6.2, 2.4)	0.39	4.2 (-0.3, 8.7)	0.07
Child health[a]	-0.1 (-0.3, 0.2)	0.64	1.0 (0.5, 1.5)	<0.001	-1.1 (-1.9, -0.3)	0.01	0.9 (0.1, 1.7)	0.04
PNC and FP[b]	-0.1 (-0.2, 0.1)	0.54	0.1 (-0.4, 0.5)	0.78	-0.6 (-1.3, 0.1)	0.07	0.7 (0.0, 1.4)	0.05
HIV visits[c]	-0.8 (-1.6, 0.0)	0.04	-1.4 (-3.1, 0.3)	0.11	0.2 (-2.4, 2.7)	0.90	1.9 (-0.7, 4.6)	0.15
Chronic care[d]	-0.1 (-0.3, 0.1)	0.44	0.2 (-0.3, 0.6)	0.52	-0.3 (-1.0, 0.4)	0.37	0.3 (-0.5, 1.0)	0.47
Men ≥ 15	-0.2 (-0.5, 0.1)	0.15	-0.5 (-1.1, 0.2)	0.16	0.1 (-0.9, 1.0)	0.89	0.8 (-0.2, 1.8)	0.12
Women ≥ 15	-0.6 (-1.4, 0.1)	0.10	-0.6 (-2.5, 1.3)	0.55	-1.0 (-3.9, 1.9)	0.50	2.4 (-0.6, 5.4)	0.12
Age <1	0.0 (-0.2, 0.2)	0.71	0.5 (0.0, 1.0)	0.04	-0.7 (-1.4, 0.0)	0.06	0.5 (-0.2, 1.2)	0.19
Age 1-5	0.3 (0.1, 0.4)	<0.001	0.0 (-0.3, 0.4)	0.72	-0.4 (-0.9, 0.1)	0.11	0.5 (0.0, 1.1)	0.04
Age 6-19	0.1 (-0.1, 0.2)	0.27	-0.2 (-0.6, 0.1)	0.23	-0.2 (-0.8, 0.3)	0.45	0.7 (0.1, 1.2)	0.03
Age 20-45	-0.7 (-1.3, -0.1)	0.03	-0.4 (-1.9, 1.1)	0.59	-0.7 (-3.0, 1.5)	0.52	2.0 (-0.4, 4.4)	0.10
Age >45	-0.2 (-0.6, 0.2)	0.36	-0.6 (-1.6, 0.4)	0.25	0.3 (-1.2, 1.8)	0.70	0.5 (-1.0, 2.1)	0.50

[a]Child health: visits for immunizations and growth monitoring
[b]PNC and FP: perinatal care and family planning; visits for, antenatal care, prenatal care, and/or family planning
[c]HIV visits: visits for HIV testing, antiretroviral therapy initiation, antiretroviral therapy continuation, or pharmacy pick-up
[d]Chronic care: clinical visits for hypertension and/or diabetes

For peer review only

Supplemental Figure 1. Plots of residuals around estimated mean from linear mixed effects regression models of clinic visitation for total visits (A), child health visits (B), and HIV visits (C)

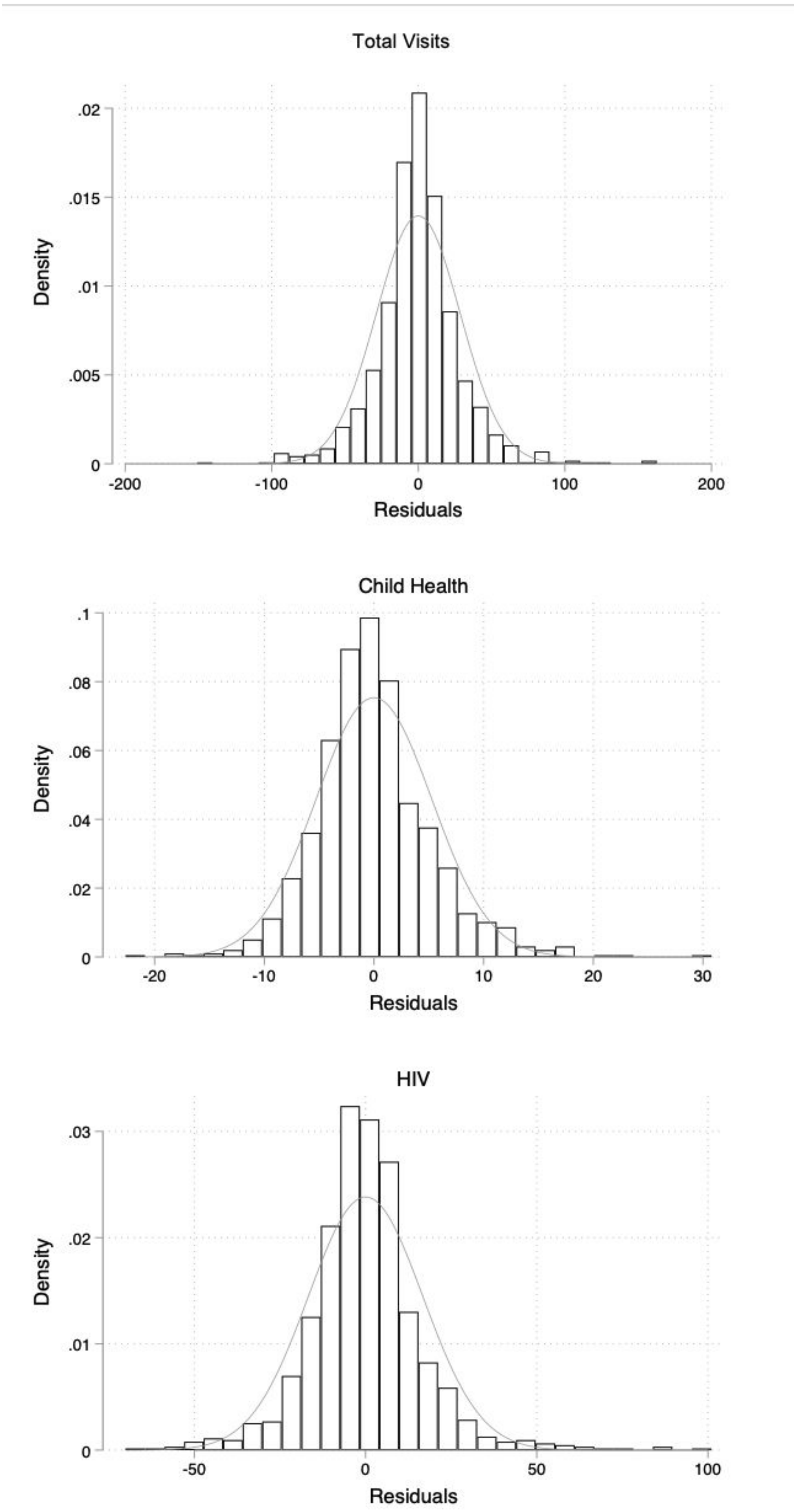

Supplemental Figure 2. Scatter and LOWESS plots with smoothed regression functions demonstrating clinic visits per day in the study observation period during 2019 and 2020 for all visits (A), child health visits (B), and HIV adults (C).

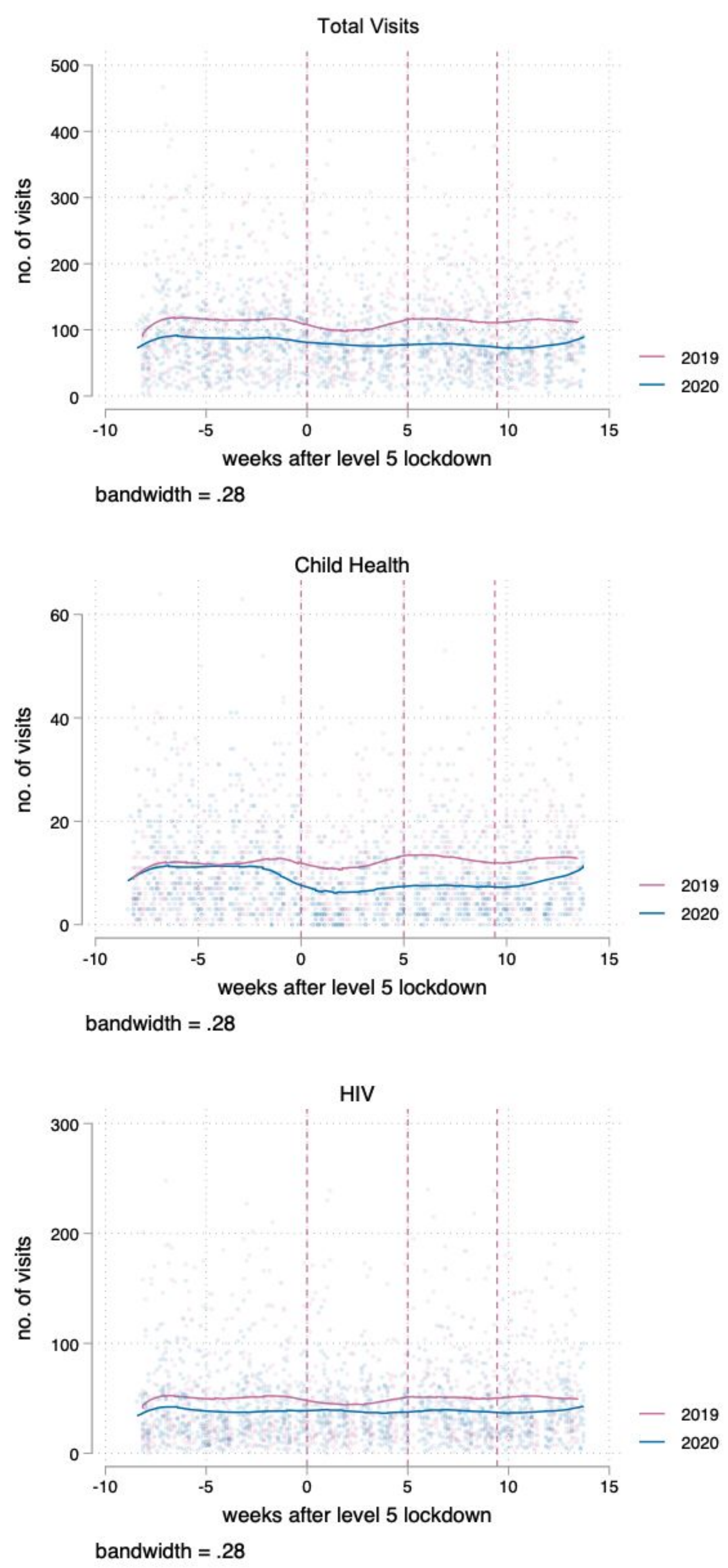

STROBE Statement—checklist of items that should be included in reports of observational studies

	Item No.	Recommendation	Page No.	Relevant text from manuscript
**Title and abstract**	1	(*a*) Indicate the study's design with a commonly used term in the title or the abstract	1	**Access to primary healthcare during lockdown measures for COVID-19 in rural South Africa: an interrupted time series analysis**
		(*b*) Provide in the abstract an informative and balanced summary of what was done and what was found	2	**Objectives**<br>The COVID-19 epidemic and public health response measures could have deleterious impacts on primary healthcare. We evaluated whether implementation of the nationwide shelter-in-place ("lockdown") order in South Africa affected ambulatory clinic visitation in rural Kwa-Zulu Natal (KZN).<br><br>**Design**<br>Prospective, longitudinal cohort study.<br><br>**Setting**<br>Data were analyzed from the Africa Health Research Institute Health and Demographic Surveillance System, which includes prospective data capture at eleven primary healthcare clinics in northern KwaZulu-Natal.<br><br>**Participants**<br>A total of 46,523 individuals made 89,476 clinic visits during our observation period.<br><br>**Exposure of Interest**<br>We conducted an interrupted time series analysis to estimate changes in outpatient clinic visitation with a primary focus on transitions from the pre-lockdown period to the level 5, 4, and 3 lockdown periods.<br><br>**Outcome Measures**<br>Daily clinic visitation at ambulatory clinics. In stratified analyses we assessed visitation for the following sub-categories: child health, perinatal care and family planning, HIV services, non-communicable diseases, and by age and sex strata.<br><br>**Results**<br>We found no change in total clinic visits/clinic/day at the time of implementation of the level 5 lockdown (change from 90.3 to 84.6 mean visits/clinic/day, 95%CI -16.5, 3.1), or at the transitions to less stringent level 4 and 3 lockdown levels. We did detect a greater than 50% reduction in child healthcare visits at the start of the level 5 lockdown from 11.9 to 4.7 visits/day (-7.1 visits/clinic/day, 95%CI -8.9, -5.3), both for children <1 and children 1-5, with a gradual return to pre-lockdown within three months after the first lockdown measure. In contrast, we found no drop in clinic visitation in adults at the start of the level 5 lockdown, or related to HIV care (from 37.5 to 45.6, 8.0 visits/clinic/day, 95%CI 2.1, 13.8).<br><br>**Conclusions**<br>In rural KZN, we identified a significant, albeit temporary, reduction in child healthcare visitation but general resilience of adult ambulatory care provision during the first four months of the lockdown. Future work should

For peer review only - http://bmjopen.bmj.com/site/about/guidelines.xhtml

				explore the impacts of the circulating epidemic on primary care provision and longer-term impacts of reduced child visitation on outcomes in the region.
**Introduction**				
Background/ rationale	2	Explain the scientific background and rationale for the investigation being reported	5	Of particular concern is how social fear and reduced access to basic public health services might impact morbidity and mortality for non-COVID health conditions. Modeling studies have suggested that even modest reductions in child healthcare access could result in 100,000s of additional deaths in low and middle-income countries.[23] Similar concerns have been raised by the Academy of Science of South Africa and others about provision of chronic disease care among adults. UNAIDS has warned that non-pharmaceutical interventions could challenge manufacturing and supply chains of HIV therapeutics, and modeling estimates suggest that such disruptions could cause as many if not more HIV-related deaths than COVID-19-related deaths. Although empiric data on health outcomes remain sparse, there have been significant reductions in tuberculosis testing in South Africa during the early phases of the lockdown, indicating an interruption in critical services for the most common cause of death in the country. There is also historical precedent from other recent communicable disease outbreaks. Primary healthcare access was significantly impacted during prior infectious disease epidemics, such as Ebola virus disease, resulting in increases in morbidity and mortality. Yet, whether and the extent to which similar effects will be seen during the COVID-19 epidemic is not known.
Objectives	3	State specific objectives, including any prespecified hypotheses	5	We sought to assess the impact of these lockdown orders in response to the COVID-19 epidemic in South Africa on access to basic healthcare services. We analysed data on clinic visitation at 11 ambulatory public health clinics in northern KwaZulu-Natal, collected routinely as part of a demographic health and surveillance system (HDSS) by the Africa Health Research Institute (AHRI). We hypothesized that there would be immediate and substantial reductions in clinic visitation after the institution of the lockdown measure, and that this would pertain to routine clinical care such as immunizations, perinatal care, and chronic disease management in adults.
**Methods**				
Study design	4	Present key elements of study design early in the paper	5	We conducted an interrupted time series analysis to estimate changes in clinic visitation in rural KwaZulu-Natal from before to after the national lockdown implementation on 27th March 2020. To do so we fitted linear mixed effects regression models by restricted maximum likelihood with daily clinic visits as the primary outcome of interest. Our primary exposure of interest was time period, divided into four periods: 1) the pre-lockdown period starting 60-days prior to the initial level 5 lockdown until 27th March 2020; 2) the level 5 lockdown period from 28th March through 30th April 2020; 3) the level 4 lockdown level from 1st of May through 31st of May; and 4) the level 3 lockdown level from 1st of June through our data abstraction date (30th of June). For our primary outcome, we estimated the stepwise change in mean visits per clinic on the date of implementation lockdown level.[37] We included a fixed effect for day of the week, a random clinic-specific intercepts, and random clinic-specific slopes on time in our models. We excluded weekends because the study clinics do not provide non-urgent ambulatory care services on weekends. We excluded dates from observation when AHRI staff members who perform data capture for the Clinic-link system were not working, including national holidays and staff trainings.
Setting	5	Describe the setting, locations, and relevant dates, including periods of recruitment, exposure, follow-up, and data collection	5-7	This analysis was conducted using data collected by the AHRI HDSS in the uMkhanyakude district of the KwaZulu-Natal Province. The HDSS comprises a complete census across a geographic area of approximately 850 km^2; it is a rural region with a single peri-urban centre, KwaMsane, a town of approximately 30,000 residents. The region ranks among the lowest nationwide in terms of health indicators and socioeconomic status.[34] Approximately 1 in 5 adult men and 2 in 5 adult women are living with HIV.[35] Tuberculosis incidence is among the highest in the world, and above the national average of 577 per 100,000 individuals when last measured in 2015.[36]<br>Since 2000 AHRI has collected data on births, deaths, migrations through thrice annual data collection encounters across a catchment area of 20,000 households (over 100,000 resident individuals).[34] In 2017, AHRI began placing

For peer review only - http://bmjopen.bmj.com/site/about/guidelines.xhtml

				clinic research assistants at each of the 11 government-run public health clinics in the area. These research staff operate in partnership with the Department of Health, but outside of the standard Health Management Information System (HMIS). For each person who presents to clinic, they collect demographic information and the self-reported reason(s) for the clinical visit. We link data between the HDSS and the clinic medical record system electronically using a unifying identification code for each resident of the catchment area. AHRI holds memoranda of understanding with the Provincial and District Department of Health that permit extraction of health record data from primary care and hospital sites for linkage to the household surveillance dataset.
Participants	6	(*a*) *Cohort study*—Give the eligibility criteria, and the sources and methods of selection of participants. Describe methods of follow-up<br>*Case-control study*—Give the eligibility criteria, and the sources and methods of case ascertainment and control selection. Give the rationale for the choice of cases and controls<br>*Cross-sectional study*—Give the eligibility criteria, and the sources and methods of selection of participants	5-6	The HDSS comprises a complete census across a geographic area of approximately 850 km^2; it is a rural region with a single peri-urban centre, KwaMsane, a town of approximately 30,000 residents.<br><br>We included all individuals who presented to ambulatory clinic in the 11 regional clinics in the study catchment area during our study observation period. There were no age or sex exclusions.
		(*b*) *Cohort study*—For matched studies, give matching criteria and number of exposed and unexposed<br>*Case-control study*—For matched studies, give matching criteria and the number of controls per case		
Variables	7	Clearly define all outcomes, exposures, predictors, potential confounders, and effect modifiers. Give diagnostic criteria, if applicable	7	We conducted an interrupted time series analysis to estimate changes in clinic visitation in rural KwaZulu-Natal from before to after the national lockdown implementation on 27th March 2020. To do so we fit mixed effects linear regression models by restricted maximum likelihood with daily clinic visits as the primary outcome of interest. Our primary exposure of interest was time period, divided into the pre-lockdown period 60-days prior to the lockdown date, and the lockdown-period, starting 27th March 2020 through 30th April 2020, the last day before South Africa transitioned to a Level 4 lockdown. We estimated the change in clinic mean visits per clinic at the date of the lockdown and the change in mean visits per clinic over time after the lockdown using regression discontinuity methods,[26] which allows us to estimate both the immediate impact of the lockdown and trend in visit daily after it went into place. We included a fixed effect for day of the week and a random effect for clinic. We excluded weekends because most of the ambulatory clinics observed do not operate on weekends. We excluded dates from observation when AHRI staff members who perform data capture for the Clinic-link system were not working, including national holidays and staff trainings.<br><br>Our primary outcome of interest was the number of clinic visits for any reason per facility per day. In secondary analyses, we stratified models by visit type restricted to: 1) child health visits (immunizations and growth monitoring); 2) antenatal care, postnatal care, and family planning; 3) HIV services (including antiretroviral therapy initiation, antiretroviral therapy continuation, and chronic care medical dispensing program visits); and 4) chronic care of non-communicable diseases (hypertension and diabetes). Clinic visit for more than one reason were treated as visits for both conditions. We also conducted stratified analyses by age category (<1, 1-5, 6-19, 20-45, and >45 years old) and by women and men aged 15 years or older.
Data sources/	8*	For each variable of interest, give sources of data and details of methods of assessment (measurement). Describe	*7*	In 2017, AHRI began placing clinic research assistants at each of the 11 government-run public health clinics in the area. These research staff operate in partnership with the Department of Health, but outside of the standard Health

For peer review only - http://bmjopen.bmj.com/site/about/guidelines.xhtml

measurement		comparability of assessment methods if there is more than one group		Management Information System (HMIS). For each person who presents to clinic, they collect demographic information and the self-reported reason(s) for the clinical visit. We link data between the HDSS and the clinic medical record system electronically using a unifying identification code for each resident of the catchment area. For this analysis, we included all individuals who presented to ambulatory clinic in the 11 regional clinics in the study catchment are during our observation period. There were no age or sex exclusions. AHRI holds memoranda of understanding with the Provincial and District Department of Health that permit extraction of health record data from primary care and hospital sites for linkage to the household surveillance dataset.
Bias	9	Describe any efforts to address potential sources of bias	8	We performed a number of secondary and sensitivity analyses to assess model validity and robustness of our findings. In addition to estimating stepwise changes after each lockdown order and change, we estimated trends in weekly visits during each period to determine whether immediate changes were sustained over each period. We graphically depicted the residuals in the model to assess the normality assumption of our linear model structure. To check the robustness of model assumptions about changes from the pre-lockdown to the level 5 lockdown periods, we conducted multiple sensitivity analyses: 1) we specified a Poisson mixed effects regression model in place of a linear model; 2) we fitted linear and Poisson generalized estimating equation (GEE) models clustered by facility; 3) we specified an auto-regressive covariance structure in place of an exchangeable structure in the GEE models; and 4) to assess for the possibility of seasonal changes over multiple years, we constructed LOWESS plots to visually inspect clinical visitation trends over the same observation periods in 2020 versus 2018 and 2019 and fitted a difference-in-differences model that included year (2019 versus 2020) and time (characterized as pre-lockdown versus level 5 lockdown) to assess whether changes before and after the level 5 lockdown differed by calendar year.
Study size	10	Explain how the study size was arrived at	6	For this analysis, we included all individuals who presented to ambulatory clinic in the 11 regional clinics in the study catchment are during our observation period. There were no age or sex exclusions.

Continued on next page

For peer review only - http://bmjopen.bmj.com/site/about/guidelines.xhtml

Quantitative variables	11	Explain how quantitative variables were handled in the analyses. If applicable, describe which groupings were chosen and why		N/A
Statistical methods	12	(*a*) Describe all statistical methods, including those used to control for confounding	8	We conducted an interrupted time series analysis to estimate changes in clinic visitation in rural KwaZulu-Natal from before to after the national lockdown implementation on 27th March 2020. To do so we fitted linear mixed effects regression models by restricted maximum likelihood with daily clinic visits as the primary outcome of interest. Our primary exposure of interest was time period, divided into four periods: 1) the pre-lockdown period starting 60-days prior to the initial level 5 lockdown until 27th March 2020; 2) the level 5 lockdown period from 28th March through 30th April 2020; 3) the level 4 lockdown level from 1st of May through 31st of May; and 4) the level 3 lockdown level from 1st of June through our data abstraction date (30th of June). For our primary outcome, we estimated the stepwise change in mean visits per clinic on the date of implementation lockdown level.[37] We included a fixed effect for day of the week, a random clinic-specific intercepts, and random clinic-specific slopes on time in our models. We excluded weekends because the study clinics do not provide non-urgent ambulatory care services on weekends. We excluded dates from observation when AHRI staff members who perform data capture for the Clinic-link system were not working, including national holidays and staff trainings.
		(*b*) Describe any methods used to examine subgroups and interactions	8	In secondary analyses, we stratified models by visit type<br><br>We also conducted stratified analyses by age category (<1, 1-5, 6-19, 20-45, and >45 years old) and by women and men aged 15 years or older.
		(*c*) Explain how missing data were addressed	8	We excluded weekends because the study clinics do not provide non-urgent ambulatory care services on weekends. We excluded dates from observation when AHRI staff members who perform data capture for the Clinic-link system were not working, including national holidays and staff trainings.
		(*d*) *Cohort study*—If applicable, explain how loss to follow-up was addressed<br>*Case-control study*—If applicable, explain how matching of cases and controls was addressed<br>*Cross-sectional study*—If applicable, describe analytical methods taking account of sampling strategy		
		(*e*) Describe any sensitivity analyses	7-8	We performed a number of secondary and sensitivity analyses to assess model validity and robustness of our findings. In addition to estimating stepwise changes after each lockdown order and change, we estimated trends in weekly visits during each period to determine whether immediate changes were sustained over each period. We graphically depicted the residuals in the model to assess the normality assumption of our linear model structure. To check the robustness of model assumptions about changes from the pre-lockdown to the level 5 lockdown periods, we conducted multiple sensitivity analyses: 1) we specified a Poisson mixed effects regression model in place of a linear model; 2) we fitted linear and Poisson generalized estimating equation (GEE) models clustered by facility; 3) we specified an auto-regressive covariance structure in place of an exchangeable structure in the GEE models; and 4) to assess for the possibility of seasonal changes over multiple years, we constructed LOWESS plots to visually inspect clinical visitation trends over the same observation periods in 2020 versus 2018 and 2019 and fitted a difference-in-differences model that included year (2019 versus 2020) and time (characterized as pre-lockdown versus level 5 lockdown) to assess whether changes before and after the level 5 lockdown differed by calendar year.

For peer review only - http://bmjopen.bmj.com/site/about/guidelines.xhtml
For peer review only

				Finally, we conducted an additional sensitivity analysis to assess for the possibility of in-migration into the HDSS catchment area during the lockdown period, which would potentially bias clinic visitation frequency upwards. To do so, we calculated annual visitation frequency at the 11 area clinics for each individual in the dataset for the year prior to the lockdown. We then compared the median number of annual visits per individual in the pre- and post-lockdown periods, and the number of individuals with exactly one visit in the past year in the two periods. If significant in-migration did occur during the lockdown period, we would expect that the median number of annual visits per individual would decrease during the lockdown, whereas the number of individuals with one visit in the past 12 months would increase.
**Results**				
Participants	13*	(a) Report numbers of individuals at each stage of study—eg numbers potentially eligible, examined for eligibility, confirmed eligible, included in the study, completing follow-up, and analysed	11	A total of 46,523 individuals made 89,476 clinic visits between 27th January – 30th June 2020 at the 11 area clinics (**Table 1**). Women and girls accounted for 67% (n=64,125) of visits. Approximately 9% of visits were made by individuals less than 1 year old (n=4,186), 1-5 year old (n=3,944), and 6-19 years old (n=4,460), respectively; whereas those 20-45 years accounted for 48% (n=22,231) and those over 46 the remaining 25% of visits (n=11,702). The most common reason for a clinic visit was ART follow-up care, comprising 43% of all visits (n=38,142), followed by visits for minor ailments (18%, n=16,204), child health (n=9,672, 11%) and hypertension (n=9,273, n=10%).
		(b) Give reasons for non-participation at each stage		N/A
		(c) Consider use of a flow diagram		
Descriptive data	14*	(a) Give characteristics of study participants (eg demographic, clinical, social) and information on exposures and potential confounders	11	A total of 46,523 individuals made 89,476 clinic visits between 27th January – 30th June 2020 at the 11 area clinics (**Table 1**). Women and girls accounted for 67% (n=64,125) of visits. Approximately 9% of visits were made by individuals less than 1 year old (n=4,186), 1-5 year old (n=3,944), and 6-19 years old (n=4,460), respectively; whereas those 20-45 years accounted for 48% (n=22,231) and those over 46 the remaining 25% of visits (n=11,702). The most common reason for a clinic visit was ART follow-up care, comprising 43% of all visits (n=38,142), followed by visits for minor ailments (18%, n=16,204), child health (n=9,672, 11%) and hypertension (n=9,273, n=10%).
		(b) Indicate number of participants with missing data for each variable of interest		N/A
		(c) *Cohort study*—Summarise follow-up time (eg, average and total amount)		N/A
Outcome data	15*	*Cohort study*—Report numbers of outcome events or summary measures over time		N/A
		Case-control study—Report numbers in each exposure category, or summary measures of exposure		
		Cross-sectional study—Report numbers of outcome events or summary measures		
Main results	16	(*a*) Give unadjusted estimates and, if applicable, confounder-adjusted estimates and their precision (eg, 95% confidence interval). Make clear which confounders were adjusted for and why they were included	9	There was an average of 90.3 (95%CI 67.5, 113.2) clinic visits per day per clinic in the pre-lockdown period. We identified a non-significant drop in visits immediately following the start of the level 5 lockdown (-6.7 visits/clinic/day, 95%CI -16.5, 3.1). The small reduction seen after the level 5 period was reversed by a non-significant stepwise increase between the level 5 and level 4 periods (increase of 11.2 visits/clinic/day, 95%CI -0.5, 23.0), and persistent clinic visitation between the end of level 4 and the start of the level 3 period (increase of 1.2

For peer review only

For peer review only - http://bmjopen.bmj.com/site/about/guidelines.xhtml

		visits/clinic/day, 95%CI -9.7, 12.1) (**Table 2, Figure 1**). There were no significant changes in trends over time in clinic visits/week in any of the pre- or post-implementation periods (**Supplemental Table 1**).
	(*b*) Report category boundaries when continuous variables were categorized	
	(*c*) If relevant, consider translating estimates of relative risk into absolute risk for a meaningful time period	

Continued on next page

For peer review only

Other analyses	17	Report other analyses done—eg analyses of subgroups and interactions, and sensitivity analyses	10	In contrast to child health visits, HIV-related clinical visits for adults did not decrease between the pre-lockdown and level 5 lockdown period from 37.6 visits/clinic/day to 45.5 visits/clinic/day (mean change of 7.9 visits/clinic/day, 95%CI 2.1, 13.8), between the level 5 lockdown and level 4 lockdown period (increase of 11.1 clinic/visits/day, 95%CI 4.1, 18.0), or between the transition from the level 4 to level 3 (increase of 4.0 visits/clinic/day, 95%CI -2.5, 10.5) (**Table 2, Figure 1**). We similarly identified resilience in family planning visits over the observation period, increasing from 7.3 visits/clinic/day in the pre-implementation period to 7.8 visits/clinic/day after transition to level 5 (+0.5 visits/clinic/day, 95%CI -1.0, 2.0) to 8.9 clinic visits/day after transition to level 4 (+1.1 visits/clinic/day, 95%CI -0.7, 3.0) and 11.0 visits/clinic/day after transition to level 3 (+2.0 visits/clinic/day, 95%CI 0.3, 3.7) for a 66% total increase from the pre-period. We did not detect changes in clinic visitation for chronic non-communicable diseases, or more broadly in clinic visitation by men or women 15 years or older.
**Discussion**				
Key results	18	Summarise key results with reference to study objectives	14	We found evidence of a significant drop in visits for childcare alongside sustained visitation in HIV and adult ambulatory clinic utilization in a rural area of South Africa during the national lockdown for the COVID-19 epidemic. Notably, visits for chronic disease, such as hypertension and diabetes, perinatal care and family planning remained reasonably constant or modestly increased. However, child health visits for immunizations and growth monitoring dropped immediately by over 50% after the start of the lockdown. With gradual increases over time during level 5 and level 3 lockdown, child visits largely returned to pre-lockdown levels in June, approximately 3 months after the lockdown began. We noted an estimated 20% increase in clinic visits for HIV immediately after the lockdown and suspect this might have reflected an urgency to collect medications prior to an anticipated interruption in clinic access or medication availability and/or national programmatic efforts to accelerate transitions to a new first-line regimen.[38] These results demonstrate concerning trends about reductions in preventative child care during the lockdown period. However, they also appeared to disprove our hypothesis about clinic visitation in adults, and potentially demonstrate a resilience in the healthcare sector during a period of concern for access to chronic and essential basic health services.
Limitations	19	Discuss limitations of the study, taking into account sources of potential bias or imprecision. Discuss both direction and magnitude of any potential bias	16	Our study should be interpreted within the context of the relatively short period (3 months) of the lockdown in South Africa. As a result, we are not yet able to assess longer-term repercussions from disruptions to income or from the epidemic itself, or longer-term effects of lapses in primary care and vaccination on health outcomes, and our results should not be generalized over longer time horizons. It is expected that economic barriers to healthcare utilization will increase as the epidemic's effects persist over time, including secondary effects from non-pharmaceutical interventions. These effects are likely to fall most heavily on those in the informal economy.[50] South Africa has taken steps to increase social support to counteract economic disruption from the epidemic and control measures.[51] Mitigating longer-term consequences may require governments and development partners to increase access to employment and other social support services during the epidemic.
Interpretation	20	Give a cautious overall interpretation of results considering objectives, limitations, multiplicity of analyses, results from similar studies, and other relevant evidence	17	In summary, we report a reduction in child wellcare visitation but resilience of the adult ambulatory health care system during the early COVID-19 epidemic and lockdown period in rural South Africa. Future work should establish if these trends are maintained, and particularly monitor access to childcare and immunizations as a result of the trends reported in this study. Finally, in rural South Africa and similar areas, efforts to balance ongoing provision of essential preventative and chronic healthcare services might be needed to ensure healthcare access remains intact while preventing nosocomial spread of COVID-19 among high-risk populations.

For peer review only - http://bmjopen.bmj.com/site/about/guidelines.xhtml
For peer review only

Generalisability	21	Discuss the generalisability (external validity) of the study results	17	Our analysis should also be interpreted within the context of our study area – one with approximately 200 reported cases of COVID-19 at the time of the analysis, but in a nation with a large epidemic (approximately 200,000 cases as of early July) with established local transmission in other areas.
**Other information**				
Funding	22	Give the source of funding and the role of the funders for the present study and, if applicable, for the original study on which the present article is based	21	Wellcome Trust. 082384/Z/07/Z/. Willem Hanekom<br>Wellcome Trust and Royal Society. 210479/Z/18/Z. Guy Harling<br>Shahmanesh is supported by National Institutes of Health under award number 5R01MH114560-03, Bill & Melinda Gates Foundation, Grant Number OPP1136774 and OPP1171600.<br>National Human Genome Research Institute of the National Institutes of Health (NIH) under Award Number U24HG006941, South African Medical Research Council (MRC-RFA-UFSP-01-2013/UKZN HIVEPI) and the South African Department of Science and Innovation (DSI). Mark J. Siedner is supported by the National Institutes of Health (R01 AI124718, R01 AG059504).

*Give information separately for cases and controls in case-control studies and, if applicable, for exposed and unexposed groups in cohort and cross-sectional studies.

Note: An Explanation and Elaboration article discusses each checklist item and gives methodological background and published examples of transparent reporting. The STROBE checklist is best used in conjunction with this article (freely available on the Web sites of PLoS Medicine at http://www.plosmedicine.org/, Annals of Internal Medicine at http://www.annals.org/, and Epidemiology at http://www.epidem.com/). Information on the STROBE Initiative is available at www.strobe-statement.org.

For peer review only

BMJ Open

Access to primary healthcare during lockdown measures for COVID-19 in rural South Africa: an interrupted time series analysis

Journal:	*BMJ Open*
Manuscript ID	bmjopen-2020-043763.R1
Article Type:	Original research
Date Submitted by the Author:	13-Sep-2020
Complete List of Authors:	Siedner, Mark; Africa Health Research Institute, Medicine; Harvard Medical School,<br>Kraemer, John; Georgetown University, Department of Health Systems Administratoin<br>Meyer, Mark; Georgetown University, Mathematics and Statistics<br>Harling, Guy; University College London, Institute for Global Health; Harvard University T H Chan School of Public Health, Department of Epidemiology<br>Mngomezulu, Thobeka; Africa Health Research Institute, Population research<br>Gabela, Patrick; Africa Health Research Institute, Population Research<br>Dlamini, Siphephelo; Africa Health Research Institute, Nursing<br>Gareta, Dickman; Africa Health Research Institute, Research Data Management<br>Majozi, Nomathamsanqa; Africa Health Research Institute, Public Engagement<br>Ngwenya, Nothando; Africa Health Research Institute, Social Sciences<br>Seeley, Janet; MRC/UVRI Uganda Research Unit On AIDS, ; London School of Hygiene and Tropical Medicine,<br>Wong, Emily; Africa Health Research Institute, Medicine<br>Iwuji, Collins; Brighton and Sussex Medical School, Sexual Health and HIV Medicine<br>Shahmanesh, Maryam; Africa Health Research Institute, Medicine<br>Hanekom, Willem; Africa Health Research Institute, Medicine<br>Herbst, Kobus; Africa Health Research Institute, Population Research
**Primary Subject Heading**:	Public health
Secondary Subject Heading:	Health services research, Public health, Infectious diseases
Keywords:	COVID-19, Public health < INFECTIOUS DISEASES, International health services < HEALTH SERVICES ADMINISTRATION & MANAGEMENT

I, the Submitting Author has the right to grant and does grant on behalf of all authors of the Work (as defined in the below author licence), an exclusive licence and/or a non-exclusive licence for contributions from authors who are: i) UK Crown employees; ii) where BMJ has agreed a CC-BY licence shall apply, and/or iii) in accordance with the terms applicable for US Federal Government officers or employees acting as part of their official duties; on a worldwide, perpetual, irrevocable, royalty-free basis to BMJ Publishing Group Ltd ("BMJ") its licensees and where the relevant Journal is co-owned by BMJ to the co-owners of the Journal, to publish the Work in this journal and any other BMJ products and to exploit all rights, as set out in our licence.

The Submitting Author accepts and understands that any supply made under these terms is made by BMJ to the Submitting Author unless you are acting as an employee on behalf of your employer or a postgraduate student of an affiliated institution which is paying any applicable article publishing charge ("APC") for Open Access articles. Where the Submitting Author wishes to make the Work available on an Open Access basis (and intends to pay the relevant APC), the terms of reuse of such Open Access shall be governed by a Creative Commons licence – details of these licences and which Creative Commons licence will apply to this Work are set out in our licence referred to above.

Other than as permitted in any relevant BMJ Author's Self Archiving Policies, I confirm this Work has not been accepted for publication elsewhere, is not being considered for publication elsewhere and does not duplicate material already published. I confirm all authors consent to publication of this Work and authorise the granting of this licence.

For peer review only

Access to primary healthcare during lockdown measures for COVID-19 in rural South Africa: an interrupted time series analysis

Authors

Mark J. Siedner*[1,2], John D. Kraemer[3], Mark J. Meyer[4], Guy Harling[1,5], Thobeka Mngomezulu[1], Patrick Gabela[1], Siphephelo Dlamini[1], Dickman Gareta[1], Nomathamsanqa Majozi[1], Nothando Ngwenya[6], Janet Seeley[6], Emily Wong[1], Collins Iwuji[1,7], Maryam Shahmanesh[1,5], Willem Hanekom[1], Kobus Herbst[1]

Affiliations:

[1]Department of Population Health, African Health Research Institute, KwaZulu-Natal, South Africa

[2]Medical Practice Evaluation Center, Massachusetts General Hospital, Boston, MA, USA

[3]Department of Health Systems Administration, Georgetown University, Washington DC, USA

[4]Department of Mathematics and Statistics, Georgetown University, Washington DC, USA

[5]Institute for Global Health, University College London, London, UK

[6]Department of Social Science, Africa Health Research Institute, KwaZulu-Natal, South Africa

[7]Department of Sexual Health and HIV Medicine, Brighton and Sussex Medical School, Brighton, UK

*Corresponding Author:

Mark J. Siedner

Africa Health Research Institute

KwaZulu-Natal, South Africa

mark.siedner@ahri.org

For peer review only

For peer review only

For peer review only - http://bmjopen.bmj.com/site/about/guidelines.xhtml

Abstract

Objectives

We evaluated whether implementation of lockdown orders in South Africa affected ambulatory clinic visitation in rural Kwa-Zulu Natal (KZN).

Design

Observational cohort

Setting

Data were analyzed from eleven primary healthcare clinics in northern KwaZulu-Natal.

Participants

A total of 46,523 individuals made 89,476 clinic visits during our observation period.

Exposure of Interest

We conducted an interrupted time series analysis to estimate changes in clinic visitation with a focus on transitions from the pre-lockdown to the level 5, 4, and 3 lockdown periods.

Outcome Measures

Daily clinic visitation at ambulatory clinics. In stratified analyses we assessed visitation for the following sub-categories: child health, perinatal care and family planning, HIV services, non-communicable diseases, and by age and sex strata.

Results

We found no change in total clinic visits/clinic/day at the time of implementation of the level 5 lockdown (change from 90.3 to 84.6 mean visits/clinic/day, 95%CI -16.5, 3.1), or at the transitions to less stringent level 4 and 3 lockdown levels. We did detect a greater than 50% reduction in child healthcare visits at the start of the level 5 lockdown from 11.9 to 4.7 visits/day (-7.1 visits/clinic/day, 95%CI -8.9, -5.3), both for children <1 and children 1-5, with a gradual return to pre-lockdown within three

For peer review only

months after the first lockdown measure. In contrast, we found no drop in clinic visitation in adults at the start of the level 5 lockdown, or related to HIV care (from 37.5 to 45.6, 8.0 visits/clinic/day, 95%CI 2.1, 13.8).

Conclusions

In rural KZN, we identified a significant, albeit temporary, reduction in child healthcare visitation but general resilience of adult ambulatory care provision during the first four months of the lockdown. Future work should explore the impacts of the circulating epidemic on primary care provision and longer-term impacts of reduced child visitation on outcomes in the region.

For peer review only

Strengths and Limitations of the Study

- This study is strengthened by a large number of observations per clinic, collection of data using research staff and methods external to the programmatic healthcare system (which can be interrupted during external shocks), and longitudinal observation allowing comparison of the pre- and post-period
- The study was conducted within the public health sector of South Africa, and is generalizable to similarly rural, resource-limited settings, and observation during the lockdown period prior to widespread local COVID-19 transmission
- Our exposure of interest (i.e. the lockdown measures), were not randomly allocated, so there is a risk, however small, that a simultaneous exogenous shock could be responsible for the changes in clinic visitation seen
- The dataset is purely quantitative and focused on clinic visitation events, which prevents deeper exploration of the root causes of trends noted

Keywords
COVID-19, South Africa, Primary Care, Health Systems Resilience, Health and Demographic Surveillance System

For peer review only

For peer review only - http://bmjopen.bmj.com/site/about/guidelines.xhtml

Introduction

COVID-19 was declared a global pandemic by the World Health Organization on 11th March 2020, and it has spared no region of the world. Early in the epidemic, the greatest numbers of cases have been reported in Asia, Europe and North America, with more recent dissemination within Latin America and Africa.[1] Although South Africa and a handful of other low-resource settings have reported widespread epidemics, limited testing and surveillance capabilities make it difficult to assess how widely the pandemic has spread in such settings. Such regions are believed to be at particular risk of severe epidemics, due to over-crowding, lower access to clean water and sanitation services, and inherent shortages in health system infrastructure for detection and management of disease.[2-9]

In response, most nations in sub-Saharan Africa have implemented non-pharmacologic interventions to attempt to prevent large scale epidemics. These measures, which include restrictions on large gatherings, work and school attendance, travel, and in their most stringent forms, shelter-in-place orders, are believed to reduce disease transmission.[10-13] However, instituting these measures is

For peer review only

also associated with deleterious economic and social, impacts, including large projected reductions in manufacturing, access to employment and basic necessities, and educational advancement; and these effects appear to be greatest among those in lower income and vulnerability categories.[14-19] Across the sub-Saharan African region, the Economic Commission for Africa projects an approximate 1.4% contraction in gross domestic product and that 25 million people are susceptible to entering extreme poverty.[20] Some have hypothesized that non-pharmaceutical interventions might be less effective in settings with large informal economies and limited ability to respond to increases in cases of severe disease,[21] and that their risks might outweigh their benefits.[22]

Of particular concern is how social fear and reduced access to basic public health services might impact morbidity and mortality for non-COVID health conditions. Modeling studies have suggested that even modest reductions in child healthcare access could result in 100,000s of additional deaths in low and middle-income countries.[23] Similar concerns have been raised by the Academy of Science of South Africa and others about provision of chronic disease care among adults.[24 25] UNAIDS

For peer review only - http://bmjopen.bmj.com/site/about/guidelines.xhtml

has warned that non-pharmaceutical interventions could challenge manufacturing and supply chains of HIV therapeutics,[26] and modeling estimates suggest that such disruptions could cause as many if not more HIV-related deaths than COVID-19-related deaths.[27] Although empiric data on health outcomes remain sparse, there have been significant reductions in tuberculosis testing in South Africa during the early phases of the lockdown,[28] indicating an interruption in critical services for the most common cause of death in the country.[29] There is also historical precedent from other recent communicable disease outbreaks. Primary healthcare access was significantly impacted during prior infectious disease epidemics, such as Ebola virus disease, resulting in increases in morbidity and mortality.[30 31] Yet, whether and the extent to which similar effects will be seen during the COVID-19 epidemic is not known.

On 27th March, 2020, South Africa instituted a nationwide shelter-in-place order, termed in South Africa as a Level 5 lockdown. The level 5 order included closure of schools and all non-essential business, restrictions on public transport, and restrictions on movement. Restrictions on movement during the level 5 lockdown

For peer review only - http://bmjopen.bmj.com/site/about/guidelines.xhtml

specifically required that individuals remain in their place of residence, with the exceptions of "performing an essential service, obtaining an essential good or service, collecting a social grant, pension, or seeking emergency, life-saving or chronic medical attention." Over the following months, the restrictions gradually eased from level 5 down to 4 at the end of April and level 3 at the end of May, which corresponded with lifting restrictions on intra-province movement, preinitiation of public transportation, and allowed for reopening of schools and many business. [32 33] Because the healthcare sector was deemed an essential service throughout the entire lockdown period, no restrictions were placed on access to or delivery of healthcare services.

We sought to assess the impact of these lockdown orders in response to the COVID-19 epidemic in South Africa on access to basic healthcare services. We analysed data on clinic visitation at 11 ambulatory public health clinics in northern KwaZulu-Natal, collected routinely as part of a demographic health and surveillance system (HDSS) by the Africa Health Research Institute (AHRI). We hypothesized that there would be immediate and substantial reductions in clinic visitation after the

For peer review only - http://bmjopen.bmj.com/site/about/guidelines.xhtml

institution of the lockdown measure, and that this would pertain to routine clinical care such as immunizations, perinatal care, and chronic disease management in adults.

Methods

Study Setting

This analysis was conducted using data collected by the AHRI HDSS in the uMkhanyakude district of the KwaZulu-Natal Province. The HDSS comprises a complete census across a geographic area of approximately 850 km^2; it is a rural region with a single peri-urban centre, KwaMsane, a town of approximately 30,000 residents. The region ranks among the lowest nationwide in terms of health indicators and socioeconomic status.[34] Approximately 1 in 5 adult men and 2 in 5 adult women are living with HIV.[35] Tuberculosis incidence is among the highest in the world, and above the national average of 577 per 100,000 individuals when last measured in 2015.[36]

Data Collection

For peer review only - http://bmjopen.bmj.com/site/about/guidelines.xhtml

Since 2000 AHRI has collected data on births, deaths, migrations through thrice annual data collection encounters across a catchment area of 20,000 households (over 100,000 resident individuals).[34] In 2017, AHRI began placing clinic research assistants at each of the 11 government-run public health clinics in the area. These research staff operate in partnership with the Department of Health, but outside of the standard Health Management Information System (HMIS). For each person who presents to clinic, they collect demographic information and the self-reported reason(s) for the clinical visit. We link data between the HDSS and the clinic medical record system electronically using a unifying identification code for each resident of the catchment area. For this analysis, we included all individuals who presented to ambulatory clinic in the 11 regional clinics in the study catchment area during our observation period. There were no age or sex exclusions. AHRI holds memoranda of understanding with the Provincial and District Department of Health that permit extraction of health record data from primary care and hospital sites for linkage to the household surveillance dataset.

For peer review only - http://bmjopen.bmj.com/site/about/guidelines.xhtml

Study Design

We conducted an interrupted time series analysis to estimate changes in clinic visitation in rural KwaZulu-Natal from before to after the national lockdown implementation on 27th March 2020. To do so we fitted linear mixed effects regression models by restricted maximum likelihood with daily clinic visits as the primary outcome of interest. Our primary exposure of interest was time period, divided into four periods: 1) the pre-lockdown period starting 60-days prior to the initial level 5 lockdown until 27th March 2020; 2) the level 5 lockdown period from 28th March through 30th April 2020; 3) the level 4 lockdown level from 1st of May through 31st of May; and 4) the level 3 lockdown level from 1st of June through our data abstraction date (30th of June). For our primary outcome, we estimated the stepwise change in mean visits per clinic on the date of implementation lockdown level.[37] We included a fixed effect for day of the week, a random clinic-specific intercepts, and random clinic-specific slopes on time in our models. We excluded weekends because the study clinics do not provide non-urgent ambulatory care services on weekends. We excluded dates from observation when AHRI staff members who

For peer review only

perform data capture for the Clinic-link system were not working, including national holidays and staff trainings.

Our primary outcome of interest was the number of clinic visits for any reason per clinic. In secondary analyses, we stratified models by visit type restricted to: 1) child health visits (immunizations and growth monitoring); 2) antenatal care, postnatal care, and family planning; 3) HIV services (including antiretroviral therapy initiation, antiretroviral therapy continuation, and chronic care medical dispensing program visits); and 4) chronic care of non-communicable diseases (hypertension and diabetes). Clinic visits for more than one reason were treated as visits for both conditions. We also conducted stratified analyses by age category (<1, 1-5, 6-19, 20-45, and >45 years old) and by women and men aged 15 years or older.

We performed a number of secondary and sensitivity analyses to assess model validity and robustness of our findings. In addition to estimating stepwise changes after each lockdown order and change, we estimated trends in weekly visits during each period to determine whether immediate changes were sustained over each

For peer review only - http://bmjopen.bmj.com/site/about/guidelines.xhtml

period. We graphically depicted the residuals in the model to assess the normality assumption of our linear model structure. To check the robustness of model assumptions about changes from the pre-lockdown to the level 5 lockdown periods, we conducted multiple sensitivity analyses: 1) we specified a Poisson mixed effects regression model in place of a linear model; 2) we fitted linear and Poisson generalized estimating equation (GEE) models clustered by facility; 3) we specified an auto-regressive covariance structure in place of an exchangeable structure in the GEE models; and 4) to assess for the possibility of seasonal changes over multiple years, we constructed LOWESS plots to visually inspect clinical visitation trends over the same observation periods in 2020 versus 2018 and 2019 and fitted a difference-in-differences model that included year (2019 versus 2020) and time (characterized as pre-lockdown versus level 5 lockdown) to assess whether changes before and after the level 5 lockdown differed by calendar year.

Finally, we conducted an additional sensitivity analysis to assess for the possibility of in-migration into the HDSS catchment area during the lockdown period, which would potentially bias clinic visitation frequency upwards. To do so, we calculated annual

For peer review only

visitation frequency at the 11 area clinics for each individual in the dataset for the year prior to the lockdown. We then compared the median number of annual visits per individual in the pre- and post-lockdown periods, and the number of individuals with exactly one visit in the past year in the two periods. If significant in-migration did occur during the lockdown period, we would expect that the median number of annual visits per individual would decrease during the lockdown, whereas the number of individuals with one visit in the past 12 months would increase.

Three study investigators designed a statistical plan prior to all analyses (MJS, JDK, MJM). The initial analysis plan included fitting mixed effects models with random effects by clinic and inspecting trends in clinic visitation changes from the pre- to level 5 lockdown period for the overall cohort, and by visit sub-type. We also initially included plans for sensitivity analyses, including fitting of generalized estimating equations and additions of random slopes to our models as robustness checks. In response to reviewer requests and with updates to the lockdown characteristics from levels 5 to 4 to 3 during the review process, we conducted a number of post-hoc analyses, including construction of LOWESS plots and fitting additional models to

For peer review only - http://bmjopen.bmj.com/site/about/guidelines.xhtml

assess for seasonal trends in visitation by year, and fitting mixed effects Poisson models. All statistical analyses were conducted using Stata and R.

Patient and Public Involvement

This protocol was reviewed and approved by the AHRI Community Advisory Board, who contributed to the study design and selection of collection measures. Results of studies from the HDSS project are routinely shared with the community through public communications and road shows conducted by the AHRI Public Engagement Department. Final, all study protocols are reviewed and approved by the District and Provincial Department of Health, and AHRI holds memoranda of understanding with the Provincial and District Departments of Health that outline methods of extraction of health record data from primary care sites for linkage to the household surveillance dataset.

For peer review only

For peer review only - http://bmjopen.bmj.com/site/about/guidelines.xhtml

Ethical Approval

187 The protocol was reviewed and approved by the University of KwaZulu-Natal Biomedical Research Ethics Committee under reference BE290/16 and the KwaZulu Department of Health Research Committee.

Results

A total of 46,523 individuals made 89,476 clinic visits between 27th January – 30th June 2020 at the 11 area clinics (**Table 1**). Women and girls accounted for 67% (n=64,125) of visits. Approximately 9% of visits were made by individuals less than 1 year old (n=4,186), 1-5 year old (n=3,944), and 6-19 years old (n=4,460), respectively; whereas those 20-45 years accounted for 48% (n=22,231) and those over 46 the remaining 25% of visits (n=11,702). The most common reason for a clinic visit was ART follow-up care, comprising 43% of all visits (n=38,142), followed by visits for minor ailments (18%, n=16,204), child health (n=9,672, 11%) and hypertension (n=9,273, n=10%).

For peer review only - http://bmjopen.bmj.com/site/about/guidelines.xhtml

There was an average of 90.3 (95%CI 67.5, 113.2) clinic visits per day per clinic in the pre-lockdown period. We identified a non-significant drop in visits immediately following the start of the level 5 lockdown (-6.7 visits/clinic/day, 95%CI -16.5, 3.1). The small reduction seen after the level 5 period was reversed by a non-significant stepwise increase between the level 5 and level 4 periods (increase of 11.2 visits/clinic/day, 95%CI -0.5, 23.0), and persistent clinic visitation between the end of level 4 and the start of the level 3 period (increase of 1.2 visits/clinic/day, 95%CI -9.7, 12.1) (**Table 2, Figure 1**). There were no significant changes in trends over time in clinic visits/week in any of the pre- or post-implementation periods (**Supplemental Table 1**).

In contrast, child health visits suffered a 60% stepwise drop at the initiation of the level 5 lockdown (from 11.9 to 4.6 visits/day/clinic, mean change of -7.1 visits, 95%CI -9.0, -5.3), but remained steady during the transition between the level 5 and level 4 (-0.5 visits/clinic/day, 95%CI -2.7, 1.7), and again between the level 4 and level 3 lockdowns (-0.4 visits/clinic/day, 95%CI -2.5, 1.6) (**Table 2, Figure 1**). The reduction in child visits at the time of the level 5 lockdown occurred both among

For peer review only - http://bmjopen.bmj.com/site/about/guidelines.xhtml

children under 1 (mean decrease of -5.4 visits, 95%CI -7.0, -3.7) and those 1-5 years old (mean decrease of -5.5 visits, 95%CI -6.7, -4.4). We did detect fluctuating trends in child visitation during the lockdown periods, with increases of approximately 1 visit/clinic/week in levels 5 and 3, resulting in a similar number of mean visits/clinic/day one month into to the level 3 lockdown on 30th June as compared to just prior to the level 5 lockdown (11.2 visits/clinic/day [95%CI 7.4, 14.8] vs 11.9 visits/clinic/day [95%CI 8.6, 15.1], **Supplemental Table 1**).

In contrast to child health visits, HIV-related clinical visits for adults did not decrease between the pre-lockdown and level 5 lockdown period from 37.6 visits/clinic/day to 45.5 visits/clinic/day (mean change of 7.9 visits/clinic/day, 95%CI 2.1, 13.8), between the level 5 lockdown and level 4 lockdown period (increase of 11.1 clinic/visits/day, 95%CI 4.1, 18.0), or between the transition from the level 4 to level 3 (increase of 4.0 visits/clinic/day, 95%CI -2.5, 10.5) (**Table 2, Figure 1**). We similarly identified resilience in family planning visits over the observation period, increasing from 7.3 visits/clinic/day in the pre-implementation period to 7.8 visits/clinic/day after transition to level 5 (+0.5 visits/clinic/day, 95%CI -1.0, 2.0) to 8.9 clinic visits/day

For peer review only - http://bmjopen.bmj.com/site/about/guidelines.xhtml

after transition to level 4 (+1.1 visits/clinic/day, 95%CI -0.7, 3.0) and 11.0 visits/clinic/day after transition to level 3 (+2.0 visits/clinic/day, 95%CI 0.3, 3.7) for a 66% total increase from the pre-period. We did not detect changes in clinic visitation for chronic non-communicable diseases, or more broadly in clinic visitation by men or women 15 years or older.

Graphical depictions of residuals from our linear models demonstrated normally distributed residuals for the total visit, HIV, and child visit models, supporting the specification of a linear model to estimate trends in clinic visitation (**Supplemental Figure 1**). We found no evidence of changes in clinic visitation during this same period in 2019 to suggest seasonal effects, either graphically in LOWESS plots, or in difference-in-differences in models including both 2019 and 2020 with pre and post-lockdown periods (**Table 3, Supplemental Figure 2**). Results were robust to modelling assumptions in the sensitivity analyses (**Table 3**).

Finally, we did not detect evidence of meaningful in-migration during the lockdown period. The median number of visits in the past year per individual attending the

For peer review only

clinic slightly increased from the pre-lockdown period to the lockdown period (mean 5.8 [SD 0.02] vs. 5.9 [SD 0.03], P<0.001). This pattern was similar among people attending clinic for HIV-specific visits (mean 6.5 [SD 0.02] vs mean 6.6 [SD 0.04], P=0.01). The number of people with exactly one visit in the past year also did not meaningfully increase during the observation period with 1,960 (February), 2,115 (March), 1,573 (April), 1,893 (May), and 1,986 (June) visits made by individuals with exactly one annual clinic visit over the prior 12 months.

Discussion

We found evidence of a significant drop in visits for childcare alongside sustained visitation in HIV and adult ambulatory clinic utilization in a rural area of South Africa during the national lockdown for the COVID-19 epidemic. Notably, visits for chronic disease, such as hypertension and diabetes, perinatal care and family planning remained reasonably constant or modestly increased. However, child health visits for immunizations and growth monitoring dropped immediately by over 50% after the start of the lockdown. With gradual increases over time during level 5 and level 3 lockdown, child visits largely returned to pre-lockdown levels in June, approximately

For peer review only

3 months after the lockdown began. We noted an estimated 20% increase in clinic visits for HIV immediately after the lockdown and suspect this might have reflected an urgency to collect medications prior to an anticipated interruption in clinic access or medication availability and/or national programmatic efforts to accelerate transitions to a new first-line regimen.[38] These results demonstrate concerning trends about reductions in preventative child care during the lockdown period. However, they also appeared to disprove our hypothesis about clinic visitation in adults, and potentially demonstrate a resilience in the healthcare sector during a period of concern for access to chronic and essential basic health services.

The key demographic population in our study that experienced significant drops in clinic visitation was children. Child health visits appeared to have modestly rebounded during the lockdown, and eventually return to their pre-lockdown state. Although these data do not suggest the cause of reduced visitation in children, multiple possible factors might be considered. We hypothesize that limited options for childcare for families with multiple children might prevent caregivers from being able to bring individual children to clinic. Moreover, in contrast to, for example, HIV

For peer review only - http://bmjopen.bmj.com/site/about/guidelines.xhtml

wellcare visits, well child visits rarely involve medication refills so might be prioritized lower for families. Whatever the cause, our findings are in keeping with data from elsewhere. In Hangzhou, China, pediatric healthcare visits dropped by nearly 75% during the peak of the epidemic and lockdown periods.[39] In the U.S. vaccination rates in children substantially declined after a national emergency was declared in response to the COVID-19 epidemic.[40] Modeling analyses using Lived Saves Tool (LiST) have suggested that a 15% reduction in maternal and child health coverage could result in over 250,000 additional deaths.[23] The World Health Organization has also projected significant increases in deaths due to malaria in children under 5 in endemic regions with disruptions in malaria care and insecticide treated bednet distribution.[41] Although empiric data on healthcare access in South Africa remain scarce, work to date has suggested significant reductions in tuberculosis testing in laboratory databases, and reports of interruptions in care in community-based surveys.[28 42] Previous disease epidemics in sub-Saharan Africa have also been associated with lapses in primary care access, and drops in facility based births and child healthcare access.[30 31 43 44] Consequently, future work should investigate the impacts of even modest drops in vaccination rates and child health outcomes, to

For peer review only

For peer review only - http://bmjopen.bmj.com/site/about/guidelines.xhtml

better assess whether the drop we identified resulted in longer term health effects, and whether catch-up vaccination campaigns might help limit the fallout of such interruptions.[45]

Maintaining healthcare access during the epidemic requires a careful balance of primary healthcare provision and protection of vulnerable populations from COVID-19 infection. In other settings, there have been multiple reports of late and severe presentations to care for non-COVID-19 conditions, putatively due to decreased access to care or fear of nosocomial infection at healthcare facilities.[46-48] At the time of our data abstraction at the end of June during the level 3 lockdown period, fewer than 200 cases of COVID-19 infection had been reported in uMkhanyakude District.[49] Thus, our data largely reflect impacts of lockdown measures prior to an epidemic with significant local transmission. Clinics in this district instituted symptom screening at the entryway to clinics, with referral of individuals meeting criteria for persons under investigation to regional COVID-19 testing centres. Future work should revisit the impacts of the epidemic itself on access to primary healthcare.

For peer review only - http://bmjopen.bmj.com/site/about/guidelines.xhtml

322 The COVID-19 epidemic has also led to calls for decentralized care to minimize exposure for high-risk populations,[50] including those with chronic non-communicable disease, HIV, a history of tuberculosis-related lung disease, and those of older-ages. The lockdown was instituted rapidly in South Africa, before substantial decentralized care systems could be put in place. However, an important unanswered question is how such programs will affect access to care and epidemic transmission in high-risk populations, including the elderly and those with immunosuppressing conditions.

Our study should be interpreted within the context of the relatively short period (3 months) of the lockdown in South Africa. As a result, we are not yet able to assess longer-term repercussions from disruptions to income or from the epidemic itself, or longer-term effects of lapses in primary care and vaccination on health outcomes, and our results should not be generalized over longer time horizons. It is expected that economic barriers to healthcare utilization will increase as the epidemic's effects persist over time, including secondary effects from non-pharmaceutical interventions. These effects are likely to fall most heavily on those in the informal economy.[51] South Africa has taken steps to increase social support to counteract economic

For peer review only - http://bmjopen.bmj.com/site/about/guidelines.xhtml

disruption from the epidemic and control measures.[52] Mitigating longer-term consequences may require governments and development partners to increase access to employment and other social support services during the epidemic.

Our study had multiple strengths. First, our data collection procedures are led by research staff who remained in place during the lockdown period, so these data are not affected by barriers to data collection (e.g., interruptions in staff transportation or workplace access). This is important, since many routine health information systems could be expected to suffer lapses during external shocks to the healthcare system. Second, our study was able to access data collected across 11 clinical centres within a large HDSS, which provided significant power to detect even small interruptions to health care access. A key potential limitation to our study is that it is predicated on the assumption that there were no other external factors that would have caused interruptions to the health care system on or after 27th March 2020 (e.g., power outage, inclement weather).We are unaware of any such shock and believe this to be a minor risk. We saw no evidence of a seasonal effect after comparing our results to similar time periods in 2019. Our analysis should also be interpreted within the

For peer review only - http://bmjopen.bmj.com/site/about/guidelines.xhtml

context of our study area – one with approximately 200 reported cases of COVID-19 at the time of the analysis, but in a nation with a large epidemic (approximately 200,000 cases as of early July) with established local transmission in other areas.

In summary, we report a reduction in child wellcare visitation but resilience of the adult ambulatory health care system during the early COVID-19 epidemic and lockdown period in rural South Africa. Future work should establish if these trends are maintained, and particularly monitor access to childcare and immunizations as a result of the trends reported in this study. Finally, in rural South Africa and similar areas, efforts to balance ongoing provision of essential preventative and chronic healthcare services might be needed to ensure healthcare access remains intact while preventing nosocomial spread of COVID-19 among high-risk populations.

For peer review only

For peer review only - http://bmjopen.bmj.com/site/about/guidelines.xhtml

Table 1. Ambulatory clinic visits at 11 region clinics in rural KwaZulu Natal during 27 January 2020 – 30 June 2020 by sex and age and clinic visit type

	**Total*** **n (%)**	**Male** **n (%)**	**Female** **n (%)**	**<1 year** **n (%)**	**1-5 years** **n (%)**	**6-19 years** **n (%)**	**20-45 years** **n (%)**	**>45 years** **n (%)**
Total visits	89,476 (100%)	25,318 (28.3%)	64,125 (71.7%)	7,734 (8.6%)	5,646 (6.3%)	7,429 (8.3%)	43,116 (48.2%)	25,551 (28.6%)
Child health	9,672 (10.8%)	4,808 (49.7%)	4,846 (50.1%)	6,712 (69.4%)	2,750 (28.4%)			
PNC and FP[a]	7,879 (8.8%)	13 (0.2%)	7,866 (99.8%)	9 (0.1%)	2 (0.0%)	1,266 (16.1%)	6,570 (83.4%)	32 (0.4%)
HIV visit[b]	42,585 (47.6%)	11,152 (26.2%)	31,425 (73.8%)	44 (0.1%)	215 (0.5%)	2,549 (6.0%)	27,218 (63.9%)	12,559 (29.5%)
Chronic care[c]	10,052 (11.2%)	2,143 (21.3%)	7,909 (78.7%)	1 (0.0%)	2 (0.0%)	11 (0.1%)	647 (6.4%)	9,391 (93.4%)
Minor ailment	2,738 (3.1%)	988 (36.1%)	1,750 (63.9%)	133 (4.9%)	471 (17.2%)	384 (14.0%)	1,084 (39.6%)	666 (24.3%)
All other visits	18,043 (20.2%)	6,378 (35.4%)	11,658 (64.6%)	842 (4.7%)	2,222 (12.3%)	3,112 (17.3%)	8,390 (46.5%)	3,477 (19.3%)

*Visit types are not mutually exclusive so column totals may exceed 100%

[a]PNC and FP: Perinatal care and family planning; visits for, antenatal care, prenatal care, and/or family planning

[b]HIV visits: visits for HIV testing, antiretroviral therapy initiation, antiretroviral therapy continuation, or pharmacy pick-up

[c]Chronic care: visits for hypertension and/or diabetes

For peer review only - http://bmjopen.bmj.com/site/about/guidelines.xhtml

Table 2. Mixed effects regression model results demonstrating changes in mean visits/clinic, by visit type and demographic strata, in the pre-lockdown period, and after each transition to level 5, level 4, and level 3 lockdowns in uMkhanyakude District, KwaZulu-Natal South Africa.

Model	Mean daily visits per clinic at time of lockdown period (intercept)	Stepwise change in visits/clinic/day at start of level 5 lockdown	*P*-value	Stepwise change in visits/clinic/day at start of level 4 lockdown	*P*-value	Stepwise change in clinic visits/clinic/day at start of level 3 lockdown	*P*-value
Total visits	90.3 (67.1, 113.5)	-6.7 (-16.4, 3.0)	0.18	11.3 (-0.3, 22.9)	0.06	1.2 (-9.6, 12.0)	0.83
Child health[a]	11.9 (8.6, 15.1)	-7.1 (-8.9, -5.3)	<0.001	-0.5 (-2.6, 1.6)	0.65	-0.4 (-2.4, 1.6)	0.67
PNC and FP[b]	7.3 (4.2, 10.3)	0.5 (-1.0, 2.0)	0.51	1.1 (-0.7, 2.9)	0.22	2.0 (0.3, 3.7)	0.02
HIV visits[c]	37.5 (24.4, 50.7)	8.0 (2.3, 13.7)	0.01	11.0 (4.2, 17.8)	0.001	4.0 (-2.3, 10.3)	0.22
Chronic care[d]	9.5 (7.1, 11.8)	-0.3 (-1.9, 1.3)	0.70	-0.15 (-2.1, 1.8)	0.88	0.4 (-1.4, 2.2)	0.65
Men ≥ 15	15.4 (10.8, 19.9)	1.5 (-0.7, 3.6)	0.17	2.3 (-0.2, 4.9)	0.07	0.8 (-1.5, 3.2)	0.50
Women ≥ 15	52.9 (38.1, 67.7)	3.2 (-3.3, 9.6)	0.33	9.6 (1.9, 17.3)	0.01	2.1 (-5.1, 9.3)	0.57
Age <1	10.6 (7.6, 13.5)	-5.3 (-6.9, -3.7)	<0.001	0.2 (-1.6, 2.1)	0.83	-0.4 (-2.1, 1.4)	0.67
Age 1-5	8.9 (7.4, 10.5)	-5.6 (-6.7, -4.4)	<0.001	-0.2 (-1.5, 1.1)	0.80	-0.8 (-2.1, 0.4)	0.18
Age 6-19	8.0 (6.2, 9.8)	-0.7 (-1.9, 0.6)	0.29	1.5 (0.0, 3.0)	0.05	-0.1 (-1.5, 1.3)	0.91
Age 20-45	39.3 (26.3, 52.2)	4.4 (-0.7, 9.5)	0.09	7.5 (1.4, 13.6)	0.02	2.2 (-3.5, 7.8)	0.45
Age >45	25.2 (19.2, 31.3)	0.4 (-3.0, 3.7)	0.83	2.9 (-1.1, 6.9)	0.15	0.5 (-3.2, 4.3)	0.78

[a]Child health: visits for immunizations and growth monitoring
[b]PNC and FP: perinatal care and family planning; visits for, antenatal care, prenatal care, and/or family planning
[c]HIV visits: visits for HIV testing, antiretroviral therapy initiation, antiretroviral therapy continuation, or pharmacy pick-up
[d]Chronic care: clinical visits for hypertension and/or diabetes

For peer review only - http://bmjopen.bmj.com/site/about/guidelines.xhtml

Table 3. Sensitivity analyses, demonstrating results of the main regression model and alternate models.

All Visits	Mean daily visits per clinic at time of lockdown period	Stepwise change in clinic visits/day at start of level 5 lockdown	*P*-value
Primary model	90.3 (67.1, 113.5)	-6.7 (-16.4, 3.0)	0.18
Poisson[a] mixed effects model	92.0 (59.3, 124.7)	-6.9 (-11.0, -2.8)	0.001
Linear GEE (exchangeable correlation matrix)	89.2 (67.0, 111.4)	-6.4 (-16.8, 4.08)	0.23
Poisson GEE[a] (exchangeable correlation matrix)	89.2 (84.7, 93.6)	-6.6 (-8.7, -4.5)	<0.001
Linear GEE (autoregressive correlation matrix)	90.2 (73.2, 107.2)	-5.4 (-27.4, 16.6)	0.63
Poisson GEE[a] (autoregressive correlation matrix)	88.4 (85.0, 91.9)	-5.0 (-9.4, -0.6)	0.03
Difference-in-Differences[b]	96.3 (63.6, 129.0)	3.4 (-5.5, 12.4)	0.45
**Childcare Visits**			
Primary model	11.9 (8.6, 15.1)	-7.1 (-8.9, -5.3)	<0.001
Poisson[a] mixed effects model	12.3 (7.1, 17.5)	-7.7 (-11.1, -4.4)	<0.001
Linear GEE (exchangeable correlation matrix)	11.8 (8.6, 15.0)	-7.1 (-9.0, -5.2)	<0.001
Poisson GEE[a] (exchangeable correlation matrix)	12.0 (10.5, 13.5)	-7.5 (-8.4, -6.6)	<0.001
Linear GEE (autoregressive correlation matrix)	11.9 (9.5, 14.4)	-6.4 (-9.9, -2.9)	<0.001
Poisson GEE[a] (autoregressive correlation matrix)	11.9 (10.7, 13.1)	-6.7 (-8.1, -5.4)	<0.001
Difference-in-Differences[b]	11.8 (8.0, 15.7)	-4.0 (-5.5, -2.5)	<0.001
**HIV Visits**			

For peer review only - http://bmjopen.bmj.com/site/about/guidelines.xhtml

Primary model	37.5 (24.4, 50.7)	8.0 (2.3, 13.7)	0.01
Poisson[a] mixed effects model	39.2 (22.7, 55.8)	9.0 (4.5, 13.5)	<0.001
Linear GEE (exchangeable correlation matrix)	37.7 (25.3, 50.1)	8.1 (2.2, 14.0)	0.007
Poisson GEE[a] (exchangeable correlation matrix)	37.7 (34.8, 40.6)	8.7 (7.2, 10.3)	<0.001
Linear GEE (autoregressive correlation matrix)	38.9 (29.3, 48.5)	6.1 (-6.2, 18.5)	0.33
Poisson GEE[a] (autoregressive correlation matrix)	37.9 (35.7, 40.1)	5.7 (2.6, 8.8)	0.002
Difference-in-Differences[b]	43.6 (25.1, 62.1)	4.8 (-0.5, 10.1)	0.08

GEE: generalized estimating equations

[a]Poisson GEE results are presented as predictive margins and marginal effects so they represent changes on the same additive scale as the linear models.

[b]Difference-in-differences estimates are estimated as the mean of the level 5 lockdown period minus the mean of the pre-lockdown period, comparing 2020 to 2019. Estimates are based on a period-by-year interaction term fit via linear mixed models.

For peer review only

Figure Legends

Figure 1. Ambulatory clinic visitation before and after the nationwide lockdown in South Africa at eleven outpatient clinics in rural uMkhanyakude District, KwaZulu-Natal South Africa. Scatterplots represent mean clinic visitation at each clinic on weekdays during the observation period. The black fit line represents the mean visitation across all clinics estimated by post-regression margins from a linear regression model, with a regression discontinuity coefficient at the date of the lockdown (27th March 2020, red line). Gray bars represent 95% confidence intervals. The dotted blue line represents the geometic mean of the number of visits across all clinics on each day.

Supplemental Figure 1. Plots of residuals around estimated mean from linear mixed effects regression models of clinic visitation for total visits (A), child health visits (B), and HIV visits (C)

Supplemental Figure 2. Scatter and LOWESS plots with smoothed regression functions demonstrating clinic visits per day in the study observation period during 2019 and 2020 for all visits (A), child health visits (B), and HIV adults (C).

For peer review only

For peer review only - http://bmjopen.bmj.com/site/about/guidelines.xhtml

Author contributions

Contributor Role	Role Definition	
Conceptualization	Ideas; formulation or evolution of overarching research goals and aims.	MJS
Data Curation	Management activities to annotate (produce metadata), scrub data and maintain research data (including software code, where it is necessary for interpreting the data itself) for initial use and later reuse.	DG, KH
Formal Analysis	Application of statistical, mathematical, computational, or other formal techniques to analyze or synthesize study data.	MJS, MJM, JDK, GH
Funding Acquisition	Acquisition of the financial support for the project leading to this publication.	KH, WH
Investigation	Conducting a research and investigation process, specifically performing the experiments, or data/evidence collection.	KH, MJS, CI MS
Methodology	Development or design of methodology; creation of models.	KH, MJS
Project Administration	Management and coordination responsibility for the research activity planning and execution.	KH, JS, MS, NN, NM
Resources	Provision of study materials, reagents, materials, patients, laboratory samples, animals, instrumentation, computing resources, or other analysis tools.	KH
Software	Programming, software development; designing computer programs; implementation of the computer code and supporting algorithms; testing of existing code components.	KH, DG
Supervision	Oversight and leadership responsibility for the research activity planning and execution, including mentorship external to the core team.	KH, MS, TM, PG, SD

For peer review only - http://bmjopen.bmj.com/site/about/guidelines.xhtml

Contributor Role	Role Definition	
Validation	Verification, whether as a part of the activity or separate, of the overall replication/reproducibility of results/experiments and other research outputs.	KH
Visualization	Preparation, creation and/or presentation of the published work, specifically visualization/data presentation.	KH, MJS
Writing – Original Draft Preparation	Creation and/or presentation of the published work, specifically writing the initial draft (including substantive translation).	MJS, JDK
Writing – Review & Editing	Preparation, creation and/or presentation of the published work by those from the original research group, specifically critical review, commentary or revision – including pre- or post-publication stages.	KH, MJS, MJM, JS, DG, GH, EW, JDK, CI, MS, NN, TM, PG, SD, WH, NM

Transparency declaration

MJS affirms the manuscript is an honest, accurate, and transparent account of the study being reported; that no important aspects of the study have been omitted; and that any discrepancies from the study as originally planned (and, if relevant, registered) have been explained.

Data sharing

Data from the AHRI HDSS are publicly available upon request to the AHRI research repository which can be made here: https://data.africacentre.ac.za/index.php/auth/login/?destination=

Competing interests and conflicts of interest

All authors have completed the ICMJE uniform disclosure form and declare no financial relationships with any organisations that might have an interest in the submitted work in the previous three years and no other relationships or activities that could appear to have influenced the submitted work.

Funding information

Wellcome Trust. 082384/Z/07/Z/. Willem Hanekom

For peer review only - http://bmjopen.bmj.com/site/about/guidelines.xhtml

Wellcome Trust and Royal Society. 210479/Z/18/Z. Guy Harling
Shahmanesh is supported by National Institutes of Health under award number 5R01MH114560-03, Bill & Melinda Gates Foundation, Grant Number OPP1136774 and OPP1171600.
National Human Genome Research Institute of the National Institutes of Health (NIH) under Award Number U24HG006941, South African Medical Research Council (MRC-RFA-UFSP-01-2013/UKZN HIVEPI) and the South African Department of Science and Innovation (DSI).
Mark J. Siedner is supported by the National Institutes of Health (R01 AI124718, R01 AG059504).

Acknowledgements

We thank the outstanding field staff at the Africa Health Research Institute for their work in conducting the demographic health survey field work, as well as the community and the study participants for their time and efforts. The population HIV surveillance is a node of the South African Population Research Infrastructure Network (SAPRIN) and funded by the South African Department of Science and Innovation and hosted by the South African Medical Research Council. Additional thanks to Zahra Reynolds for her assistance in organizing elements of the manuscript for submission.

License
The Corresponding Author has the right to grant on behalf of all authors and does grant on behalf of all authors, an exclusive license (or non exclusive for government employees) on a worldwide basis to the BMJ Publishing Group Ltd to permit this article (if accepted) to be published in BMJ editions and any other BMJPGL products and sublicences such use and exploit all subsidiary rights, as set out in our license.

For peer review only

References

1. World Health Organization. Coronavirus disease (COVID-2019) situation reports. Available at: https://www.who.int/emergencies/diseases/novel-coronavirus-2019/situation-reports Accessed 2 May 2020.
2. Johnstone-Robertson SP, Mark D, Morrow C, et al. Social mixing patterns within a South African township community: implications for respiratory disease transmission and control. *Am J Epidemiol* 2011;174(11):1246-55. doi: 10.1093/aje/kwr251 [published Online First: 2011/11/11]
3. le Polain de Waroux O, Cohuet S, Ndazima D, et al. Characteristics of human encounters and social mixing patterns relevant to infectious diseases spread by close contact: a survey in Southwest Uganda. *BMC Infect Dis* 2018;18(1):172. doi: 10.1186/s12879-018-3073-1 [published Online First: 2018/04/13]
4. House T, Keeling MJ. Household structure and infectious disease transmission. *Epidemiol Infect* 2009;137(5):654-61. doi: 10.1017/S0950268808001416 [published Online First: 2008/10/09]
5. Nkengasong JN, Mankoula W. Looming threat of COVID-19 infection in Africa: act collectively, and fast. *Lancet* 2020;395(10227):841-42. doi: 10.1016/S0140-6736(20)30464-5 [published Online First: 2020/03/03]
6. Gilbert M, Pullano G, Pinotti F, et al. Preparedness and vulnerability of African countries against importations of COVID-19: a modelling study. *Lancet* 2020;395(10227):871-77. doi: 10.1016/S0140-6736(20)30411-6 [published Online First: 2020/02/24]
7. Kapata N, Ihekweazu C, Ntoumi F, et al. Is Africa prepared for tackling the COVID-19 (SARS-CoV-2) epidemic. Lessons from past outbreaks, ongoing pan-African public health efforts, and implications for the future. *Int J Infect Dis* 2020;93:233-36. doi: 10.1016/j.ijid.2020.02.049 [published Online First: 2020/03/03]
8. Adhikari NK, Fowler RA, Bhagwanjee S, et al. Critical care and the global burden of critical illness in adults. *Lancet* 2010;376(9749):1339-46. doi: 10.1016/S0140-6736(10)60446-1 [published Online First: 2010/10/12]
9. Sands P, Mundaca-Shah C, Dzau VJ. The Neglected Dimension of Global Security--A Framework for Countering Infectious-Disease Crises. *N Engl J Med* 2016;374(13):1281-7. doi: 10.1056/NEJMsr1600236 [published Online First: 2016/01/14]
10. Fong MW, Gao H, Wong JY, et al. Nonpharmaceutical Measures for Pandemic Influenza in Nonhealthcare Settings-Social Distancing Measures. *Emerg Infect Dis* 2020;26(5):976-84. doi: 10.3201/eid2605.190995 [published Online First: 2020/02/07]
11. Markel H, Lipman HB, Navarro JA, et al. Nonpharmaceutical interventions implemented by US cities during the 1918-1919 influenza pandemic. *Jama* 2007;298(6):644-54. doi: 10.1001/jama.298.6.644 [published Online First: 2007/08/09]
12. Ferguson NM, Cummings DA, Cauchemez S, et al. Strategies for containing an emerging influenza pandemic in Southeast Asia. *Nature* 2005;437(7056):209-14. doi: 10.1038/nature04017 [published Online First: 2005/08/05]
13. Peak CM, Childs LM, Grad YH, et al. Comparing nonpharmaceutical interventions for containing emerging epidemics. *Proceedings of the National Academy of Sciences* 2017;114(15):4023-28. doi: 10.1073/pnas.1616438114
14. Bayham J, Fenichel EP. Impact of school closures for COVID-19 on the US health-care workforce and net mortality: a modelling study. *Lancet Public Health* 2020 doi: 10.1016/S2468-2667(20)30082-7 [published Online First: 2020/04/07]
15. Keogh-Brown MR, Wren-Lewis S, Edmunds WJ, et al. The possible macroeconomic impact on the UK of an influenza pandemic. *Health Economics* 2010;19(11):1345-60. doi: 10.1002/hec.1554
16. Smith RD, Keogh-Brown MR, Barnett T, et al. The economy-wide impact of pandemic influenza on the UK: a computable general equilibrium modelling experiment. *BMJ* 2009;339:b4571. doi: 10.1136/bmj.b4571 [published Online First: 2009/11/21]

For peer review only - http://bmjopen.bmj.com/site/about/guidelines.xhtml

17. Nicola M, Alsafi Z, Sohrabi C, et al. The socio-economic implications of the coronavirus pandemic (COVID-19): A review. *International Journal of Surgery* 2020;78:185-93. doi: 10.1016/j.ijsu.2020.04.018
18. Mongey S, Pilossoph L, Weinberg A. Which workers bear the burden of social distancing policies? National Bureau of Economic Research Working Paper No. 27085. 2020 doi: 10.3386/w27085
19. Quaife M, van Zandvoort K, Gimma A, et al. The impact of COVID-19 control measures on social contacts and transmission in Kenyan informal settlements. *medRxiv* 2020 doi: 10.1101/2020.06.06.20122689 [published Online First: 7 June 2020]
20. United Nations Economic Commission for Africa. COVID-19 in Africa: Protecting Lives and Economies. Available at: https://www.uneca.org/publications/covid-19-africa-protecting-lives-and-economies; Accessed 5 August, 2020.
21. Dahab M, van Zandvoort K, Flasche S, et al. COVID-19 control in low-income settings and displaced populations: what can realistically be done? Pre-print. Available at: https://www.lshtm.ac.uk/newsevents/news/2020/covid-19-control-low-income-settings-and-displaced-populations-what-can#.XnpKCXF93Fc.email. Accessed: 1 May 2020. 2020
22. Greenstone M, Nigam V. Does Social Distancing Matter? *SSRN Electronic Journal* 2020 doi: 10.2139/ssrn.3561244
23. Roberton T, Carter ED, Chou VB, et al. Early Estimates of the Indirect Effects of the Coronavirus Pandemic on Maternal and Child Mortality in Low- and Middle-Income Countries. *SSRN Electronic Journal* 2020 doi: 10.2139/ssrn.3576549
24. The unanticipated costs of COVID-19 to South Africa's quadruple disease burden. Academy of Science of South Africa. 2020 [Available from: https://www.assaf.org.za/files/2020/ASSAf%20Health%20Committee%20Statement%20-%202%20July%202020.pdf accessed 15 July 2020.
25. Abdool Karim Q, Abdool Karim SS. COVID-19 affects HIV and tuberculosis care. *Science* 2020;369(6502):366-68. doi: 10.1126/science.abd1072 [published Online First: 2020/07/25]
26. UNAIDS. The impact of the COVID-19 response on the supply chain, availability and cost of generic antiretroviral medicines for HIV in low- and middle-income countries. Available at: https://www.unaids.org/en/resources/documents/2020/covid19-supply-chain-availability-cost-generic-arv; Accessed 25 July 2020. 2020
27. Jewell BL, Smith JA, Hallett TB. The potential impact of interruptions to HIV services: A modellign case study for South Africa. *medRxiv* 2020 doi: 10.1101/2020.04.22.20075861 [published Online First: 27 April 2020]
28. Madhi SA, Gray GE, Ismail N, et al. COVID-19 lockdowns in low- and middle-income countries: Success against COVID-19 at the price of greater costs. *South African Medical Jounral* 2020;110(8):724-26.
29. Mortality and causees of death in South Africa: Findings from death notification. Statistics South Africa. Available at: http://www.statssa.gov.za/publications/P03093/P030932017.pdf. Accessed 15th July 2020. 2020
30. Delamou A, Ayadi AME, Sidibe S, et al. Effect of Ebola virus disease on maternal and child health services in Guinea: a retrospective observational cohort study. *The Lancet Global Health* 2017;5(4):e448-e57. doi: 10.1016/s2214-109x(17)30078-5
31. Ly J, Sathananthan V, Griffiths T, et al. Facility-Based Delivery during the Ebola Virus Disease Epidemic in Rural Liberia: Analysis from a Cross-Sectional, Population-Based Household Survey. *PLOS Medicine* 2016;13(8) doi: 10.1371/journal.pmed.1002096
32. Republic of South Africa. Disaster Management Act: Regulations to address, prevent and combat the spread of Coronavirus-19 (COVID-19): Amendment. Available at: https://www.gov.za/documents/disaster-management-act-regulations-address-prevent-and-combat-spread-coronavirus-covid-19. Accessed 1 May 2020.

For peer review only - http://bmjopen.bmj.com/site/about/guidelines.xhtml

33. Regulations and Guidelines - Coronavirus COVID-19. South African Government. [Available from: https://www.gov.za/coronavirus/guidelines accessed 10th July 2020.
34. Tanser F, Hosegood V, Barnighausen T, et al. Cohort Profile: Africa Centre Demographic Information System (ACDIS) and population-based HIV survey. *Int J Epidemiol* 2008;37(5):956-62. doi: 10.1093/ije/dym211 [published Online First: 2007/11/14]
35. Vandormael A, Akullian A, Siedner M, et al. Declines in HIV incidence among men and women in a South African population-based cohort. *Nat Commun* 2019;10(1):5482. doi: 10.1038/s41467-019-13473-y [published Online First: 2019/12/04]
36. Massyn N, Peer N, English R, et al. District Health Barometer 2015/2016. Health Systems Trust. Available at: https://www.hst.org.za/publications/Pages/-District-Health-Barometer-201516.aspx; Accessed 29 April 2020. 2016
37. Kontopantelis E, Doran T, Springate DA, et al. Regression based quasi-experimental approach when randomisation is not an option: interrupted time series analysis. *Bmj* 2015;350(jun09 5):h2750-h50. doi: 10.1136/bmj.h2750
38. Dorward J, Lessells R, Drain PK, et al. Dolutegravir for first-line antiretroviral therapy in low-income and middle-income countries: uncertainties and opportunities for implementation and research. *The Lancet HIV* 2018;5(7):e400-e04. doi: 10.1016/s2352-3018(18)30093-6
39. Li H, Yu G, Duan H, et al. Changes in Children's Healthcare Visits During Coronavirus Disease-2019 Pandemic in Hangzhou, China. *The Journal of Pediatrics* 2020 doi: 10.1016/j.jpeds.2020.05.013
40. Santoli JM, Lindley MC, DeSilva MB, et al. Effects of the COVID-19 Pandemic on Routine Pediatric Vaccine Ordering and Administration — United States, 2020. *MMWR Morbidity and Mortality Weekly Report* 2020;69(19) doi: 10.15585/mmwr.mm6919e2
41. World Health Organization. The potential impact of health service disruptions on the burden of malaria: a modelling analysis for countries in sub-Saharan Africa. Available at: https://apps.who.int/iris/bitstream/handle/10665/331845/9789240004641-eng.pdf Accessed 4 May 2020.
42. Burger R, Nknonki L, Rensburg R, et al. Examining the unintended health consequences of the COVID-19 pandemic in South Africa. Wave 1 National Income Dynamics Study (NIDS) - Coronavirus Rapid Mobile Survey (CRAM). Available at: https://cramsurvey.org/wp-content/uploads/2020/07/Burger-examining-the-unintended-health-consequences.pdf; Accessed 31 July 2020. 2020
43. Takahashi S, Metcalf CJ, Ferrari MJ, et al. Reduced vaccination and the risk of measles and other childhood infections post-Ebola. *Science* 2015;347(6227):1240-2. doi: 10.1126/science.aaa3438 [published Online First: 2015/03/15]
44. Elston JWT, Moosa AJ, Moses F, et al. Impact of the Ebola outbreak on health systems and population health in Sierra Leone. *Journal of Public Health* 2015 doi: 10.1093/pubmed/fdv158
45. Nelson R. COVID-19 disrupts vaccine delivery. *Lancet Infect Dis* 2020 doi: 10.1016/S1473-3099(20)30304-2 [published Online First: 2020/04/21]
46. Tam CF, Cheung KS, Lam S, et al. Impact of Coronavirus Disease 2019 (COVID-19) Outbreak on ST-Segment-Elevation Myocardial Infarction Care in Hong Kong, China. *Circ Cardiovasc Qual Outcomes* 2020;13(4):e006631. doi: 10.1161/CIRCOUTCOMES.120.006631 [published Online First: 2020/03/18]
47. Moroni F, Gramegna M, Ajello S, et al. Collateral damage: medical care avoidance behavior among patients with acute coronary syndrome during the COVID-19 pandemic. *JACC: Case Reports* 2020 doi: 10.1016/j.jaccas.2020.04.010
48. Lazzerini M, Barbi E, Apicella A, et al. Delayed access or provision of care in Italy resulting from fear of COVID-19. *The Lancet Child & Adolescent Health* 2020;4(5):e10-e11. doi: 10.1016/s2352-4642(20)30108-5

For peer review only - http://bmjopen.bmj.com/site/about/guidelines.xhtml

49. Premier Sihle Zikalala's Media Briefing & Update on Covid19 in the Province Since the start of Level 4 Lockdown. Available at http://www.kznonline.gov.za/index.php/component/k2/item/1207-premier-sihle-zikalala-s-media-briefing-update-on-covid19-in-the-province-since-the-start-of-level-4-lockdown. Accessed 11 May 2020.
50. Wilkinson L, Grimsrud A. The time is now: expedited HIV differentiated service delivery during the COVID-19 pandemic. *Journal of the International AIDS Society* 2020;23(5) doi: 10.1002/jia2.25503
51. International Labour Organization. The The impact of the COVID-19 on the informal economy in Africa and the related policy responses. Available at: https://www.ilo.org/wcmsp5/groups/public/---africa/---ro-abidjan/documents/briefingnote/wcms_741864.pdf Accessed 3 May 2020.
52. Mogotsi B, Bearak M. South Africa is hunting down coronavirus with thousands of health workers. April 21, 2020. *Washington Post* 2020.

For peer review only

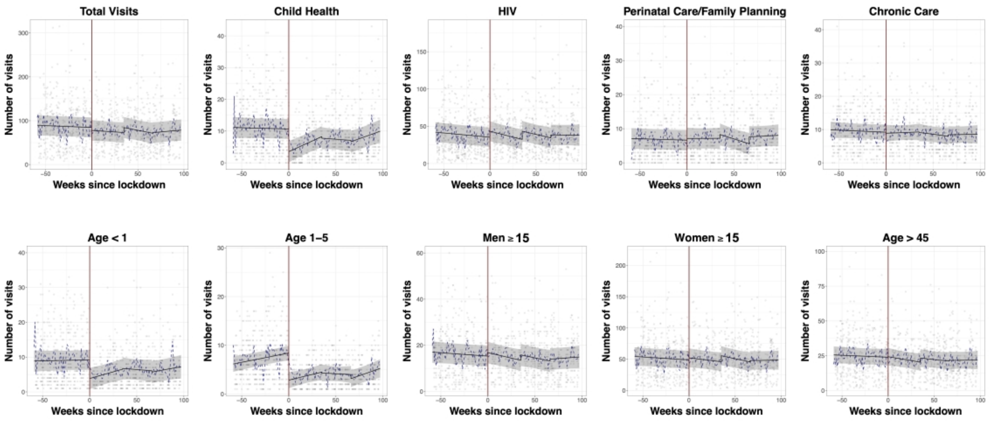

Caption : Caption : Caption : Figure 1. Ambulatory clinic visitation before and after the nationwide lockdown in South Africa at eleven outpatient clinics in rural uMkhanyakude District, KwaZulu-Natal South Africa. Scatterplots represent mean clinic visitation at each clinic on weekdays during the observation period. The black fit line represents the mean visitation across all clinics estimated by post-regression margins from a linear regression model, with a regression discontinuity coefficient at the date of the lockdown (27th March 2020, red line). Gray bars represent 95% confidence intervals. The dotted blue line represents the geometic mean of the number of visits across all clinics on each day.

2351x1322mm (72 x 72 DPI)

Supplemental Table 1. Mixed effects regression model results demonstrating changes in mean clinic visits by week, by visit type and demographic strata, in the pre- and post-lockdown period in uMkhanyakude District, KwaZulu-Natal South Africa.

Model	Weekly change in clinic visits/week during pre-lockdown period	P-value	Change in weekly change in clinic visits/week during level 5 lockdown period	*P*-value	Change in weekly change in clinic visits/week during level 4 lockdown period	*P*-value	Change in weekly change in clinic visits/week during level 3 lockdown period)	*P*-value
Total visits	-0.6 (-1.8, 0.6)	0.33	-0.4 (-3.3, 2.5)	0.80	-1.9 (-6.2, 2.4)	0.39	4.2 (-0.3, 8.7)	0.07
Child health[a]	-0.1 (-0.3, 0.2)	0.64	1.0 (0.5, 1.5)	<0.001	-1.1 (-1.9, -0.3)	0.01	0.9 (0.1, 1.7)	0.04
PNC and FP[b]	-0.1 (-0.2, 0.1)	0.54	0.1 (-0.4, 0.5)	0.78	-0.6 (-1.3, 0.1)	0.07	0.7 (0.0, 1.4)	0.05
HIV visits[c]	-0.8 (-1.6, 0.0)	0.04	-1.4 (-3.1, 0.3)	0.11	0.2 (-2.4, 2.7)	0.90	1.9 (-0.7, 4.6)	0.15
Chronic care[d]	-0.1 (-0.3, 0.1)	0.44	0.2 (-0.3, 0.6)	0.52	-0.3 (-1.0, 0.4)	0.37	0.3 (-0.5, 1.0)	0.47
Men ≥ 15	-0.2 (-0.5, 0.1)	0.15	-0.5 (-1.1, 0.2)	0.16	0.1 (-0.9, 1.0)	0.89	0.8 (-0.2, 1.8)	0.12
Women ≥ 15	-0.6 (-1.4, 0.1)	0.10	-0.6 (-2.5, 1.3)	0.55	-1.0 (-3.9, 1.9)	0.50	2.4 (-0.6, 5.4)	0.12
Age <1	0.0 (-0.2, 0.2)	0.71	0.5 (0.0, 1.0)	0.04	-0.7 (-1.4, 0.0)	0.06	0.5 (-0.2, 1.2)	0.19
Age 1-5	0.3 (0.1, 0.4)	<0.001	0.0 (-0.3, 0.4)	0.72	-0.4 (-0.9, 0.1)	0.11	0.5 (0.0, 1.1)	0.04
Age 6-19	0.1 (-0.1, 0.2)	0.27	-0.2 (-0.6, 0.1)	0.23	-0.2 (-0.8, 0.3)	0.45	0.7 (0.1, 1.2)	0.03
Age 20-45	-0.7 (-1.3, -0.1)	0.03	-0.4 (-1.9, 1.1)	0.59	-0.7 (-3.0, 1.5)	0.52	2.0 (-0.4, 4.4)	0.10
Age >45	-0.2 (-0.6, 0.2)	0.36	-0.6 (-1.6, 0.4)	0.25	0.3 (-1.2, 1.8)	0.70	0.5 (-1.0, 2.1)	0.50

[a]Child health: visits for immunizations and growth monitoring

[b]PNC and FP: perinatal care and family planning; visits for, antenatal care, prenatal care, and/or family planning

[c]HIV visits: visits for HIV testing, antiretroviral therapy initiation, antiretroviral therapy continuation, or pharmacy pick-up

[d]Chronic care: clinical visits for hypertension and/or diabetes

For peer review only

Supplemental Figure 1. Plots of residuals around estimated mean from linear mixed effects regression models of clinic visitation for total visits (A), child health visits (B), and HIV visits (C)

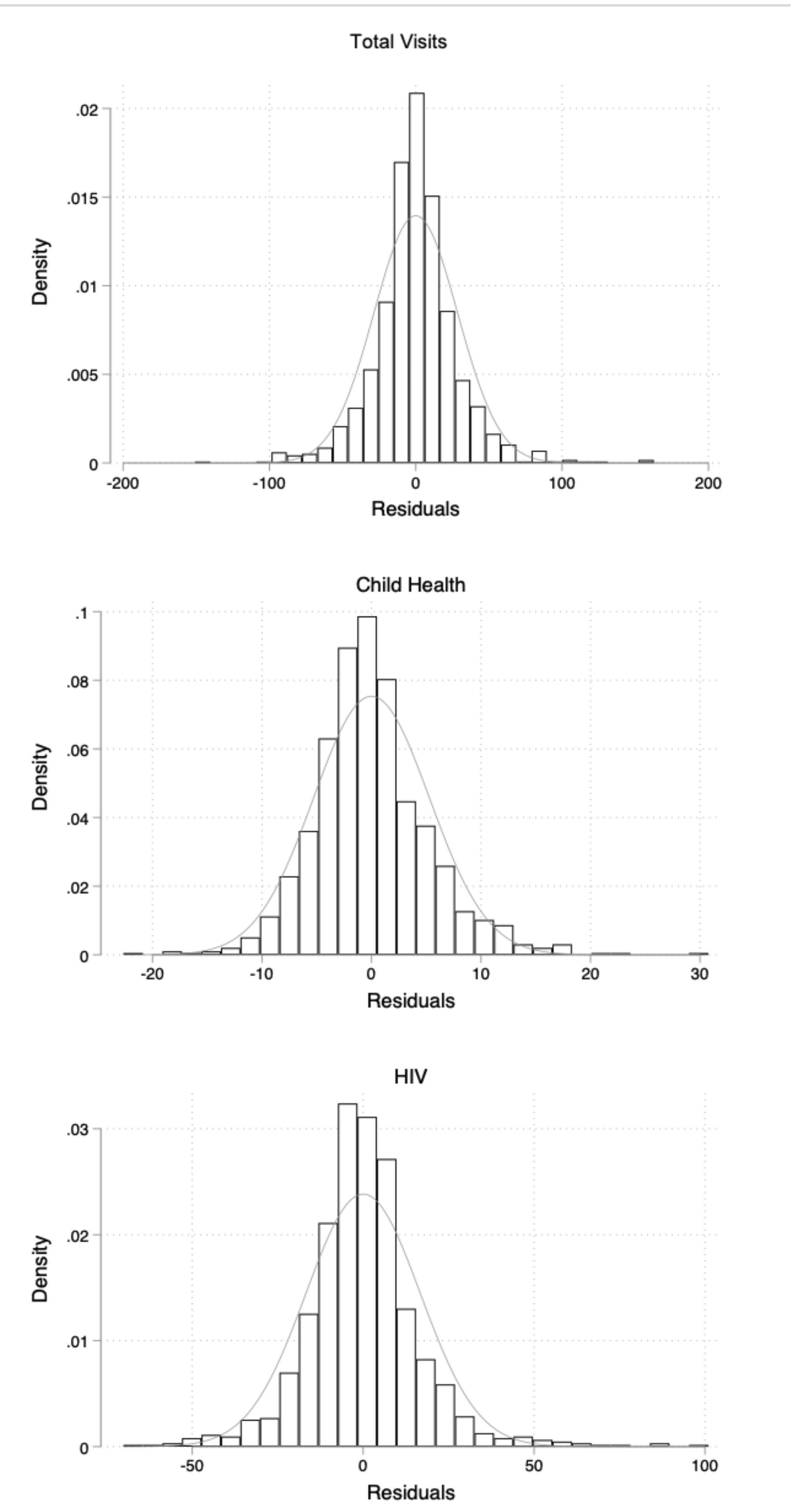

Supplemental Figure 2. Scatter and LOWESS plots with smoothed regression functions demonstrating clinic visits per day in the study observation period during 2019 and 2020 for all visits (A), child health visits (B), and HIV adults (C).

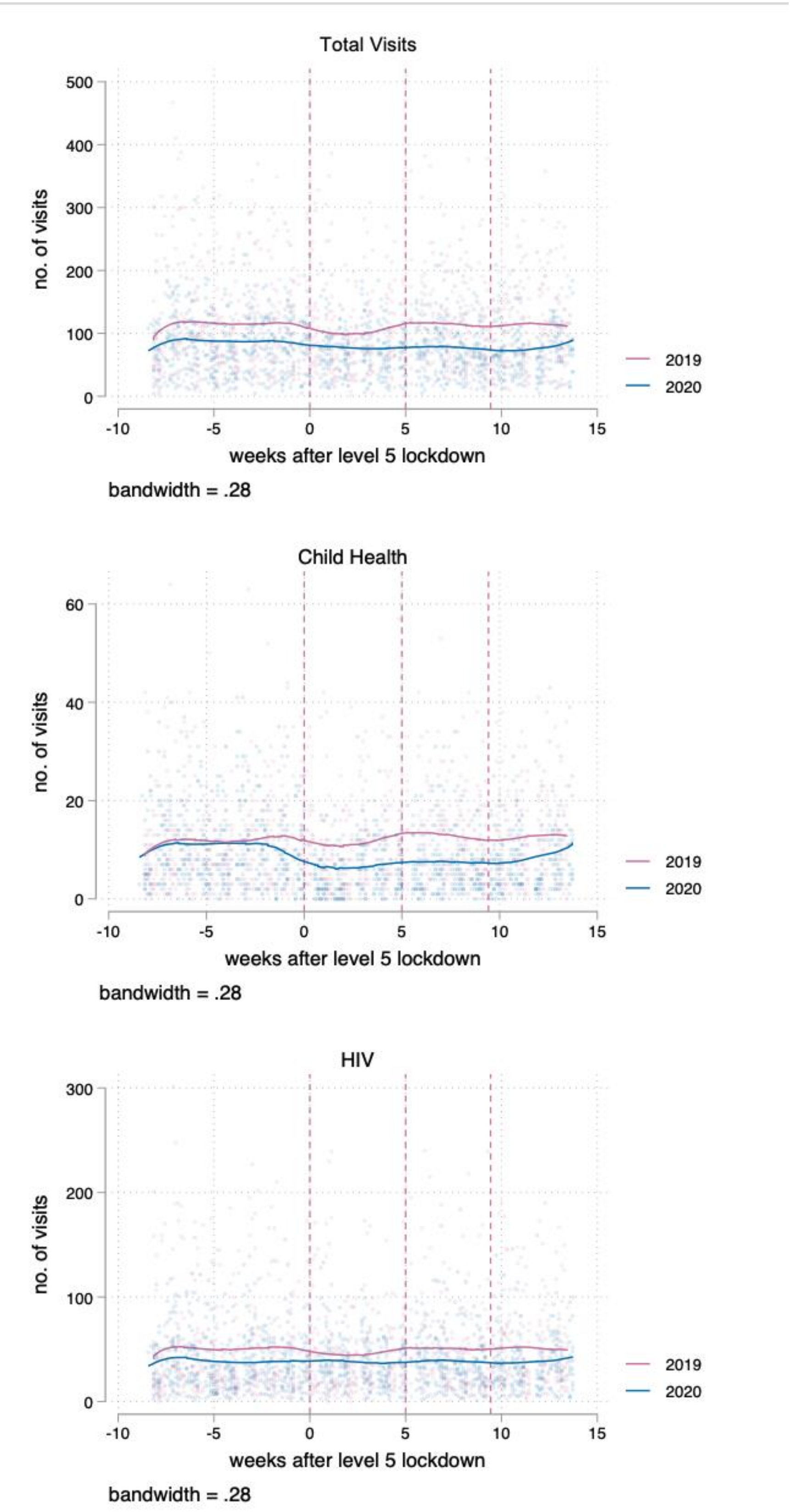

STROBE Statement—checklist of items that should be included in reports of observational studies

	Item No.	Recommendation	Page No.	Relevant text from manuscript
**Title and abstract**	1	(*a*) Indicate the study's design with a commonly used term in the title or the abstract	1	**Access to primary healthcare during lockdown measures for COVID-19 in rural South Africa: an interrupted time series analysis**
		(*b*) Provide in the abstract an informative and balanced summary of what was done and what was found	2	**Objectives**<br>The COVID-19 epidemic and public health response measures could have deleterious impacts on primary healthcare. We evaluated whether implementation of the nationwide shelter-in-place ("lockdown") order in South Africa affected ambulatory clinic visitation in rural Kwa-Zulu Natal (KZN).<br><br>**Design**<br>Prospective, longitudinal cohort study.<br><br>**Setting**<br>Data were analyzed from the Africa Health Research Institute Health and Demographic Surveillance System, which includes prospective data capture at eleven primary healthcare clinics in northern KwaZulu-Natal.<br><br>**Participants**<br>A total of 46,523 individuals made 89,476 clinic visits during our observation period.<br><br>**Exposure of Interest**<br>We conducted an interrupted time series analysis to estimate changes in outpatient clinic visitation with a primary focus on transitions from the pre-lockdown period to the level 5, 4, and 3 lockdown periods.<br><br>**Outcome Measures**<br>Daily clinic visitation at ambulatory clinics. In stratified analyses we assessed visitation for the following sub-categories: child health, perinatal care and family planning, HIV services, non-communicable diseases, and by age and sex strata.<br><br>**Results**<br>We found no change in total clinic visits/clinic/day at the time of implementation of the level 5 lockdown (change from 90.3 to 84.6 mean visits/clinic/day, 95%CI -16.5, 3.1), or at the transitions to less stringent level 4 and 3 lockdown levels. We did detect a greater than 50% reduction in child healthcare visits at the start of the level 5 lockdown from 11.9 to 4.7 visits/day (-7.1 visits/clinic/day, 95%CI -8.9, -5.3), both for children <1 and children 1-5, with a gradual return to pre-lockdown within three months after the first lockdown measure. In contrast, we found no drop in clinic visitation in adults at the start of the level 5 lockdown, or related to HIV care (from 37.5 to 45.6, 8.0 visits/clinic/day, 95%CI 2.1, 13.8).<br><br>**Conclusions**<br>In rural KZN, we identified a significant, albeit temporary, reduction in child healthcare visitation but general resilience of adult ambulatory care provision during the first four months of the lockdown. Future work should

For peer review only - http://bmjopen.bmj.com/site/about/guidelines.xhtml

				explore the impacts of the circulating epidemic on primary care provision and longer-term impacts of reduced child visitation on outcomes in the region.
**Introduction**				
Background/ rationale	2	Explain the scientific background and rationale for the investigation being reported	5	Of particular concern is how social fear and reduced access to basic public health services might impact morbidity and mortality for non-COVID health conditions. Modeling studies have suggested that even modest reductions in child healthcare access could result in 100,000s of additional deaths in low and middle-income countries.[23] Similar concerns have been raised by the Academy of Science of South Africa and others about provision of chronic disease care among adults. UNAIDS has warned that non-pharmaceutical interventions could challenge manufacturing and supply chains of HIV therapeutics, and modeling estimates suggest that such disruptions could cause as many if not more HIV-related deaths than COVID-19-related deaths. Although empiric data on health outcomes remain sparse, there have been significant reductions in tuberculosis testing in South Africa during the early phases of the lockdown, indicating an interruption in critical services for the most common cause of death in the country. There is also historical precedent from other recent communicable disease outbreaks. Primary healthcare access was significantly impacted during prior infectious disease epidemics, such as Ebola virus disease, resulting in increases in morbidity and mortality. Yet, whether and the extent to which similar effects will be seen during the COVID-19 epidemic is not known.
Objectives	3	State specific objectives, including any prespecified hypotheses	5	We sought to assess the impact of these lockdown orders in response to the COVID-19 epidemic in South Africa on access to basic healthcare services. We analysed data on clinic visitation at 11 ambulatory public health clinics in northern KwaZulu-Natal, collected routinely as part of a demographic health and surveillance system (HDSS) by the Africa Health Research Institute (AHRI). We hypothesized that there would be immediate and substantial reductions in clinic visitation after the institution of the lockdown measure, and that this would pertain to routine clinical care such as immunizations, perinatal care, and chronic disease management in adults.
**Methods**				
Study design	4	Present key elements of study design early in the paper	5	We conducted an interrupted time series analysis to estimate changes in clinic visitation in rural KwaZulu-Natal from before to after the national lockdown implementation on 27th March 2020. To do so we fitted linear mixed effects regression models by restricted maximum likelihood with daily clinic visits as the primary outcome of interest. Our primary exposure of interest was time period, divided into four periods: 1) the pre-lockdown period starting 60-days prior to the initial level 5 lockdown until 27th March 2020; 2) the level 5 lockdown period from 28th March through 30th April 2020; 3) the level 4 lockdown level from 1st of May through 31st of May; and 4) the level 3 lockdown level from 1st of June through our data abstraction date (30th of June). For our primary outcome, we estimated the stepwise change in mean visits per clinic on the date of implementation lockdown level.[37] We included a fixed effect for day of the week, a random clinic-specific intercepts, and random clinic-specific slopes on time in our models. We excluded weekends because the study clinics do not provide non-urgent ambulatory care services on weekends. We excluded dates from observation when AHRI staff members who perform data capture for the Clinic-link system were not working, including national holidays and staff trainings.
Setting	5	Describe the setting, locations, and relevant dates, including periods of recruitment, exposure, follow-up, and data collection	5-7	This analysis was conducted using data collected by the AHRI HDSS in the uMkhanyakude district of the KwaZulu-Natal Province. The HDSS comprises a complete census across a geographic area of approximately 850 km^2; it is a rural region with a single peri-urban centre, KwaMsane, a town of approximately 30,000 residents. The region ranks among the lowest nationwide in terms of health indicators and socioeconomic status.[34] Approximately 1 in 5 adult men and 2 in 5 adult women are living with HIV.[35] Tuberculosis incidence is among the highest in the world, and above the national average of 577 per 100,000 individuals when last measured in 2015.[36]<br>Since 2000 AHRI has collected data on births, deaths, migrations through thrice annual data collection encounters across a catchment area of 20,000 households (over 100,000 resident individuals).[34] In 2017, AHRI began placing

For peer review only - http://bmjopen.bmj.com/site/about/guidelines.xhtml

				clinic research assistants at each of the 11 government-run public health clinics in the area. These research staff operate in partnership with the Department of Health, but outside of the standard Health Management Information System (HMIS). For each person who presents to clinic, they collect demographic information and the self-reported reason(s) for the clinical visit. We link data between the HDSS and the clinic medical record system electronically using a unifying identification code for each resident of the catchment area. AHRI holds memoranda of understanding with the Provincial and District Department of Health that permit extraction of health record data from primary care and hospital sites for linkage to the household surveillance dataset.
Participants	6	(*a*) *Cohort study*—Give the eligibility criteria, and the sources and methods of selection of participants. Describe methods of follow-up<br>*Case-control study*—Give the eligibility criteria, and the sources and methods of case ascertainment and control selection. Give the rationale for the choice of cases and controls<br>*Cross-sectional study*—Give the eligibility criteria, and the sources and methods of selection of participants	5-6	The HDSS comprises a complete census across a geographic area of approximately 850 km^2; it is a rural region with a single peri-urban centre, KwaMsane, a town of approximately 30,000 residents.<br><br>We included all individuals who presented to ambulatory clinic in the 11 regional clinics in the study catchment area during our study observation period. There were no age or sex exclusions.
		(*b*) *Cohort study*—For matched studies, give matching criteria and number of exposed and unexposed<br>*Case-control study*—For matched studies, give matching criteria and the number of controls per case		
Variables	7	Clearly define all outcomes, exposures, predictors, potential confounders, and effect modifiers. Give diagnostic criteria, if applicable	7	We conducted an interrupted time series analysis to estimate changes in clinic visitation in rural KwaZulu-Natal from before to after the national lockdown implementation on 27th March 2020. To do so we fit mixed effects linear regression models by restricted maximum likelihood with daily clinic visits as the primary outcome of interest. Our primary exposure of interest was time period, divided into the pre-lockdown period 60-days prior to the lockdown date, and the lockdown-period, starting 27th March 2020 through 30th April 2020, the last day before South Africa transitioned to a Level 4 lockdown. We estimated the change in clinic mean visits per clinic at the date of the lockdown and the change in mean visits per clinic over time after the lockdown using regression discontinuity methods,[26] which allows us to estimate both the immediate impact of the lockdown and trend in visit daily after it went into place. We included a fixed effect for day of the week and a random effect for clinic. We excluded weekends because most of the ambulatory clinics observed do not operate on weekends. We excluded dates from observation when AHRI staff members who perform data capture for the Clinic-link system were not working, including national holidays and staff trainings.<br><br>Our primary outcome of interest was the number of clinic visits for any reason per facility per day. In secondary analyses, we stratified models by visit type restricted to: 1) child health visits (immunizations and growth monitoring); 2) antenatal care, postnatal care, and family planning; 3) HIV services (including antiretroviral therapy initiation, antiretroviral therapy continuation, and chronic care medical dispensing program visits); and 4) chronic care of non-communicable diseases (hypertension and diabetes). Clinic visit for more than one reason were treated as visits for both conditions. We also conducted stratified analyses by age category (<1, 1-5, 6-19, 20-45, and >45 years old) and by women and men aged 15 years or older.
Data sources/	8*	For each variable of interest, give sources of data and details of methods of assessment (measurement). Describe	*7*	In 2017, AHRI began placing clinic research assistants at each of the 11 government-run public health clinics in the area. These research staff operate in partnership with the Department of Health, but outside of the standard Health

For peer review only - http://bmjopen.bmj.com/site/about/guidelines.xhtml

measurement		comparability of assessment methods if there is more than one group		Management Information System (HMIS). For each person who presents to clinic, they collect demographic information and the self-reported reason(s) for the clinical visit. We link data between the HDSS and the clinic medical record system electronically using a unifying identification code for each resident of the catchment area. For this analysis, we included all individuals who presented to ambulatory clinic in the 11 regional clinics in the study catchment are during our observation period. There were no age or sex exclusions. AHRI holds memoranda of understanding with the Provincial and District Department of Health that permit extraction of health record data from primary care and hospital sites for linkage to the household surveillance dataset.
Bias	9	Describe any efforts to address potential sources of bias	8	We performed a number of secondary and sensitivity analyses to assess model validity and robustness of our findings. In addition to estimating stepwise changes after each lockdown order and change, we estimated trends in weekly visits during each period to determine whether immediate changes were sustained over each period. We graphically depicted the residuals in the model to assess the normality assumption of our linear model structure. To check the robustness of model assumptions about changes from the pre-lockdown to the level 5 lockdown periods, we conducted multiple sensitivity analyses: 1) we specified a Poisson mixed effects regression model in place of a linear model; 2) we fitted linear and Poisson generalized estimating equation (GEE) models clustered by facility; 3) we specified an auto-regressive covariance structure in place of an exchangeable structure in the GEE models; and 4) to assess for the possibility of seasonal changes over multiple years, we constructed LOWESS plots to visually inspect clinical visitation trends over the same observation periods in 2020 versus 2018 and 2019 and fitted a difference-in-differences model that included year (2019 versus 2020) and time (characterized as pre-lockdown versus level 5 lockdown) to assess whether changes before and after the level 5 lockdown differed by calendar year.
Study size	10	Explain how the study size was arrived at	6	For this analysis, we included all individuals who presented to ambulatory clinic in the 11 regional clinics in the study catchment are during our observation period. There were no age or sex exclusions.

Continued on next page

For peer review only

Quantitative variables	11	Explain how quantitative variables were handled in the analyses. If applicable, describe which groupings were chosen and why		N/A
Statistical methods	12	(*a*) Describe all statistical methods, including those used to control for confounding	8	We conducted an interrupted time series analysis to estimate changes in clinic visitation in rural KwaZulu-Natal from before to after the national lockdown implementation on 27th March 2020. To do so we fitted linear mixed effects regression models by restricted maximum likelihood with daily clinic visits as the primary outcome of interest. Our primary exposure of interest was time period, divided into four periods: 1) the pre-lockdown period starting 60-days prior to the initial level 5 lockdown until 27th March 2020; 2) the level 5 lockdown period from 28th March through 30th April 2020; 3) the level 4 lockdown level from 1st of May through 31st of May; and 4) the level 3 lockdown level from 1st of June through our data abstraction date (30th of June). For our primary outcome, we estimated the stepwise change in mean visits per clinic on the date of implementation lockdown level.[37] We included a fixed effect for day of the week, a random clinic-specific intercepts, and random clinic-specific slopes on time in our models. We excluded weekends because the study clinics do not provide non-urgent ambulatory care services on weekends. We excluded dates from observation when AHRI staff members who perform data capture for the Clinic-link system were not working, including national holidays and staff trainings.
		(*b*) Describe any methods used to examine subgroups and interactions	8	In secondary analyses, we stratified models by visit type<br><br>We also conducted stratified analyses by age category (<1, 1-5, 6-19, 20-45, and >45 years old) and by women and men aged 15 years or older.
		(*c*) Explain how missing data were addressed	8	We excluded weekends because the study clinics do not provide non-urgent ambulatory care services on weekends. We excluded dates from observation when AHRI staff members who perform data capture for the Clinic-link system were not working, including national holidays and staff trainings.
		(*d*) *Cohort study*—If applicable, explain how loss to follow-up was addressed<br>*Case-control study*—If applicable, explain how matching of cases and controls was addressed<br>*Cross-sectional study*—If applicable, describe analytical methods taking account of sampling strategy		
		(*e*) Describe any sensitivity analyses	7-8	We performed a number of secondary and sensitivity analyses to assess model validity and robustness of our findings. In addition to estimating stepwise changes after each lockdown order and change, we estimated trends in weekly visits during each period to determine whether immediate changes were sustained over each period. We graphically depicted the residuals in the model to assess the normality assumption of our linear model structure. To check the robustness of model assumptions about changes from the pre-lockdown to the level 5 lockdown periods, we conducted multiple sensitivity analyses: 1) we specified a Poisson mixed effects regression model in place of a linear model; 2) we fitted linear and Poisson generalized estimating equation (GEE) models clustered by facility; 3) we specified an auto-regressive covariance structure in place of an exchangeable structure in the GEE models; and 4) to assess for the possibility of seasonal changes over multiple years, we constructed LOWESS plots to visually inspect clinical visitation trends over the same observation periods in 2020 versus 2018 and 2019 and fitted a difference-in-differences model that included year (2019 versus 2020) and time (characterized as pre-lockdown versus level 5 lockdown) to assess whether changes before and after the level 5 lockdown differed by calendar year.

For peer review only - http://bmjopen.bmj.com/site/about/guidelines.xhtml

				Finally, we conducted an additional sensitivity analysis to assess for the possibility of in-migration into the HDSS catchment area during the lockdown period, which would potentially bias clinic visitation frequency upwards. To do so, we calculated annual visitation frequency at the 11 area clinics for each individual in the dataset for the year prior to the lockdown. We then compared the median number of annual visits per individual in the pre- and post-lockdown periods, and the number of individuals with exactly one visit in the past year in the two periods. If significant in-migration did occur during the lockdown period, we would expect that the median number of annual visits per individual would decrease during the lockdown, whereas the number of individuals with one visit in the past 12 months would increase.
**Results**				
Participants	13*	(a) Report numbers of individuals at each stage of study—eg numbers potentially eligible, examined for eligibility, confirmed eligible, included in the study, completing follow-up, and analysed	11	A total of 46,523 individuals made 89,476 clinic visits between 27th January – 30th June 2020 at the 11 area clinics (**Table 1**). Women and girls accounted for 67% (n=64,125) of visits. Approximately 9% of visits were made by individuals less than 1 year old (n=4,186), 1-5 year old (n=3,944), and 6-19 years old (n=4,460), respectively; whereas those 20-45 years accounted for 48% (n=22,231) and those over 46 the remaining 25% of visits (n=11,702). The most common reason for a clinic visit was ART follow-up care, comprising 43% of all visits (n=38,142), followed by visits for minor ailments (18%, n=16,204), child health (n=9,672, 11%) and hypertension (n=9,273, n=10%).
		(b) Give reasons for non-participation at each stage		N/A
		(c) Consider use of a flow diagram		
Descriptive data	14*	(a) Give characteristics of study participants (eg demographic, clinical, social) and information on exposures and potential confounders	11	A total of 46,523 individuals made 89,476 clinic visits between 27th January – 30th June 2020 at the 11 area clinics (**Table 1**). Women and girls accounted for 67% (n=64,125) of visits. Approximately 9% of visits were made by individuals less than 1 year old (n=4,186), 1-5 year old (n=3,944), and 6-19 years old (n=4,460), respectively; whereas those 20-45 years accounted for 48% (n=22,231) and those over 46 the remaining 25% of visits (n=11,702). The most common reason for a clinic visit was ART follow-up care, comprising 43% of all visits (n=38,142), followed by visits for minor ailments (18%, n=16,204), child health (n=9,672, 11%) and hypertension (n=9,273, n=10%).
		(b) Indicate number of participants with missing data for each variable of interest		N/A
		(c) *Cohort study*—Summarise follow-up time (eg, average and total amount)		N/A
Outcome data	15*	*Cohort study*—Report numbers of outcome events or summary measures over time		N/A
		Case-control study—Report numbers in each exposure category, or summary measures of exposure		
		Cross-sectional study—Report numbers of outcome events or summary measures		
Main results	16	(*a*) Give unadjusted estimates and, if applicable, confounder-adjusted estimates and their precision (eg, 95% confidence interval). Make clear which confounders were adjusted for and why they were included	9	There was an average of 90.3 (95%CI 67.5, 113.2) clinic visits per day per clinic in the pre-lockdown period. We identified a non-significant drop in visits immediately following the start of the level 5 lockdown (-6.7 visits/clinic/day, 95%CI -16.5, 3.1). The small reduction seen after the level 5 period was reversed by a non-significant stepwise increase between the level 5 and level 4 periods (increase of 11.2 visits/clinic/day, 95%CI -0.5, 23.0), and persistent clinic visitation between the end of level 4 and the start of the level 3 period (increase of 1.2

For peer review only

			visits/clinic/day, 95%CI -9.7, 12.1) (**Table 2, Figure 1**). There were no significant changes in trends over time in clinic visits/week in any of the pre- or post-implementation periods (**Supplemental Table 1**).
	(*b*) Report category boundaries when continuous variables were categorized		
	(*c*) If relevant, consider translating estimates of relative risk into absolute risk for a meaningful time period		

Continued on next page

For peer review only

Other analyses	17	Report other analyses done—eg analyses of subgroups and interactions, and sensitivity analyses	10	In contrast to child health visits, HIV-related clinical visits for adults did not decrease between the pre-lockdown and level 5 lockdown period from 37.6 visits/clinic/day to 45.5 visits/clinic/day (mean change of 7.9 visits/clinic/day, 95%CI 2.1, 13.8), between the level 5 lockdown and level 4 lockdown period (increase of 11.1 clinic/visits/day, 95%CI 4.1, 18.0), or between the transition from the level 4 to level 3 (increase of 4.0 visits/clinic/day, 95%CI -2.5, 10.5) (**Table 2, Figure 1**). We similarly identified resilience in family planning visits over the observation period, increasing from 7.3 visits/clinic/day in the pre-implementation period to 7.8 visits/clinic/day after transition to level 5 (+0.5 visits/clinic/day, 95%CI -1.0, 2.0) to 8.9 clinic visits/day after transition to level 4 (+1.1 visits/clinic/day, 95%CI -0.7, 3.0) and 11.0 visits/clinic/day after transition to level 3 (+2.0 visits/clinic/day, 95%CI 0.3, 3.7) for a 66% total increase from the pre-period. We did not detect changes in clinic visitation for chronic non-communicable diseases, or more broadly in clinic visitation by men or women 15 years or older.
**Discussion**				
Key results	18	Summarise key results with reference to study objectives	14	We found evidence of a significant drop in visits for childcare alongside sustained visitation in HIV and adult ambulatory clinic utilization in a rural area of South Africa during the national lockdown for the COVID-19 epidemic. Notably, visits for chronic disease, such as hypertension and diabetes, perinatal care and family planning remained reasonably constant or modestly increased. However, child health visits for immunizations and growth monitoring dropped immediately by over 50% after the start of the lockdown. With gradual increases over time during level 5 and level 3 lockdown, child visits largely returned to pre-lockdown levels in June, approximately 3 months after the lockdown began. We noted an estimated 20% increase in clinic visits for HIV immediately after the lockdown and suspect this might have reflected an urgency to collect medications prior to an anticipated interruption in clinic access or medication availability and/or national programmatic efforts to accelerate transitions to a new first-line regimen.[38] These results demonstrate concerning trends about reductions in preventative child care during the lockdown period. However, they also appeared to disprove our hypothesis about clinic visitation in adults, and potentially demonstrate a resilience in the healthcare sector during a period of concern for access to chronic and essential basic health services.
Limitations	19	Discuss limitations of the study, taking into account sources of potential bias or imprecision. Discuss both direction and magnitude of any potential bias	16	Our study should be interpreted within the context of the relatively short period (3 months) of the lockdown in South Africa. As a result, we are not yet able to assess longer-term repercussions from disruptions to income or from the epidemic itself, or longer-term effects of lapses in primary care and vaccination on health outcomes, and our results should not be generalized over longer time horizons. It is expected that economic barriers to healthcare utilization will increase as the epidemic's effects persist over time, including secondary effects from non-pharmaceutical interventions. These effects are likely to fall most heavily on those in the informal economy.[50] South Africa has taken steps to increase social support to counteract economic disruption from the epidemic and control measures.[51] Mitigating longer-term consequences may require governments and development partners to increase access to employment and other social support services during the epidemic.
Interpretation	20	Give a cautious overall interpretation of results considering objectives, limitations, multiplicity of analyses, results from similar studies, and other relevant evidence	17	In summary, we report a reduction in child wellcare visitation but resilience of the adult ambulatory health care system during the early COVID-19 epidemic and lockdown period in rural South Africa. Future work should establish if these trends are maintained, and particularly monitor access to childcare and immunizations as a result of the trends reported in this study. Finally, in rural South Africa and similar areas, efforts to balance ongoing provision of essential preventative and chronic healthcare services might be needed to ensure healthcare access remains intact while preventing nosocomial spread of COVID-19 among high-risk populations.

For peer review only - http://bmjopen.bmj.com/site/about/guidelines.xhtml
For peer review only

Generalisability	21	Discuss the generalisability (external validity) of the study results	17	Our analysis should also be interpreted within the context of our study area – one with approximately 200 reported cases of COVID-19 at the time of the analysis, but in a nation with a large epidemic (approximately 200,000 cases as of early July) with established local transmission in other areas.
**Other information**				
Funding	22	Give the source of funding and the role of the funders for the present study and, if applicable, for the original study on which the present article is based	21	Wellcome Trust. 082384/Z/07/Z/. Willem Hanekom<br>Wellcome Trust and Royal Society. 210479/Z/18/Z. Guy Harling<br>Shahmanesh is supported by National Institutes of Health under award number 5R01MH114560-03, Bill & Melinda Gates Foundation, Grant Number OPP1136774 and OPP1171600.<br>National Human Genome Research Institute of the National Institutes of Health (NIH) under Award Number U24HG006941, South African Medical Research Council (MRC-RFA-UFSP-01-2013/UKZN HIVEPI) and the South African Department of Science and Innovation (DSI). Mark J. Siedner is supported by the National Institutes of Health (R01 AI124718, R01 AG059504).

*Give information separately for cases and controls in case-control studies and, if applicable, for exposed and unexposed groups in cohort and cross-sectional studies.

Note: An Explanation and Elaboration article discusses each checklist item and gives methodological background and published examples of transparent reporting. The STROBE checklist is best used in conjunction with this article (freely available on the Web sites of PLoS Medicine at http://www.plosmedicine.org/, Annals of Internal Medicine at http://www.annals.org/, and Epidemiology at http://www.epidem.com/). Information on the STROBE Initiative is available at www.strobe-statement.org.

For peer review only - http://bmjopen.bmj.com/site/about/guidelines.xhtml
